# Chiral transcription in self-assembled tetrahedral Eu_4_L_6_ chiral cages displaying sizable circularly polarized luminescence

**DOI:** 10.1038/s41467-017-01025-1

**Published:** 2017-10-24

**Authors:** Chi-Tung Yeung, King-Him Yim, Ho-Yin Wong, Robert Pal, Wai-Sum Lo, Siu-Cheong Yan, Melody Yee-Man Wong, Dmitry Yufit, Danil E. Smiles, Laura J. McCormick, Simon J. Teat, David K. Shuh, Wing-Tak Wong, Ga-Lai Law

**Affiliations:** 1 State Key Laboratory of Chirosciences, Department of Applied Biology and Chemical Technology, The Hong Kong Polytechnic University, Hung Hom, Kowloon Hong Kong; 2 Department of Chemistry, Durham University, South Road, Durham, DH1 3LE UK; 3 Chemical Sciences Division, Lawrence Berkeley National Laboratory, One Cyclotron Road, Berkeley, CA 94720 USA; 4 Advanced Light Source, Lawrence Berkeley National Laboratory, One Cyclotron Road, Berkeley, CA 94720 USA

## Abstract

Predictable stereoselective formation of supramolecular assembly is generally believed to be an important but complicated process. Here, we show that point chirality of a ligand decisively influences its supramolecular assembly behavior. We designed three closely related chiral ligands with different point chiralities, and observe their self-assembly into europium (Eu) tetrametallic tetrahedral cages. One ligand exhibits a highly diastereoselective assembly into homochiral (either ΔΔΔΔ or ΛΛΛΛ) Eu tetrahedral cages whereas the two other ligands, with two different approaches of loosened point chirality, lead to a significant breakdown of the diastereoselectivity to generate a mixture of (ΔΔΔΔ and ΛΛΛΛ) isomers. The cages are highly emissive (luminescence quantum yields of 16(1) to 18(1)%) and exhibit impressive circularly polarized luminescence properties (|*g*_lum_|: up to 0.16). With in-depth studies, we present an example that correlates the nonlinear enhancement of the chiroptical response to the nonlinearity dependence on point chirality.

## Abstract (web summary)

Controlling the chirality of self-assembled polyhedra is a synthetic challenge. Here, the authors stereoselectively form emissive lanthanide tetrahedral cages from a series of chiral ligands, and use their circularly polarized luminescence to explore the effect of ligand point chirality on supramolecular architecture.

## Introduction

The self-assembly formation of lanthanide supramolecular architectures, particularly the relatively simple structures of mononuclear bundles (mononuclear) and dinuclear helicates (dinuclear), has received considerable attention because of their beautiful construction and peculiar spectroscopic and magnetic features^1─6^. Many pioneering and elegant examples with beautiful architectural formations have been developed by Bünzli, Piguet, and other researchers. However, higher order and sophisticated polynuclear architectures such as tetranuclear tetrahedral edifices^7─14^ and heptanuclear clusters^15, 16^ with cavities for potential encapsulation of guests are much less explored due to the difficulty in controlling the topology and coordination geometry. Similar to linear helicates, tetrahedral cages are intrinsically chiral with either a Δ or Λ configuration at each of the four vertices. However, due to the different constraints in metal coordination geometry, tetrahedral cages may result in different combinations of configurations in either (a) a homoconfigurational ΔΔΔΔ- or ΛΛΛΛ-twisted conformation or (b) a blend of heteroconfigurational ΛΔΔΔ-, ΛΛΔΔ-, ΛΛΛΔ-twisted conformers in more complex situations. Manipulation of the formation of either one of these stereoisomers in a predictable manner is key to assessing the properties of these tetrahedral cages for utilization in functional applications such as chiral guest recognition, sensing molecular cargo carriers, and asymmetric catalysis.

Great effort has been devoted to investigating the long-range linker effect^17, 18^ and chiral nonlinear effect^19^ in order to achieve stereoselective control of tetrahedral cages, but these studies mainly focused on transition metals (M)-related polyhedra, M_4_:L_6_ or M_4_:L_4_ (L is either a *C*_2_- or *C*_3_-symmetric ligand), in which the metals usually have well-defined and predictable coordination geometries^20^. In retrospect, studies on lanthanide-based systems are scarce as lanthanide ions are known to exhibit variable coordination numbers (from 3 to 12), are kinetically labile and have weak stereochemical preferences. Hence the rational design of lanthanide tetrahedral cages that possess targeted supramolecular topology with well-defined chirotopic cavities is challenging.

Introducing a predisposed point chiral moiety (point chirality) onto the ligand is a general approach to relay chiral information to an overall stereochemistry of self-assembled tetrahedral cages^21–23^, but the challenge lies in designing a suitable point chirality to selectively favor formation of one stereoisomeric structure without any depreciation of chiral information. The driving force and formation mechanism behind achieving prominent stereoselectivity for higher order coordination geometry edifices—such as in lanthanide tetrahedral cages—remain unclear and more studies are therefore necessary.

On the other hand, another way of elucidating chiral information is the use of circularly polarized luminescence (CPL), a highly sensitive and powerful tool for evaluating chiral conformational and three-dimensional structural information of the luminescent compound in the excited state, and a complementary technique to that of the ground-state-based circular dichroism (CD)^24^. However, luminescence from high-order chiral architectures are scarce. CPL spectroscopy studies of mononuclear systems has received considerable attention in recent years, particularly in sensing chemistry^25–27^; however, only a few studies have been documented regarding supramolecular polynuclear systems^15, 28–30^ and none are known regarding lanthanide tetrahedral cages. The latter is believed to be a result of both weak luminescence properties and instrumental inaccessibility^7–14^. As a result, we set off to provide some of the missing pieces of this puzzle in the chiroptical picture in order to bridge the gap between fundamental research and applications.

In the following, we utilize three pairs of ligands to form Eu tetrametallic tetrahedral cages. The desired cages are obtained by self-assembly and give rise to unexpectedly strong luminescence that in hand resulted in detectable CPL signals, which has not been observed in tetrahedral cages previously. More importantly we show that the control of helical stereoselectivity in our supramolecular assemblies is governed by the point chirality of the selected ligands **L1**–**L3**. The relationship between steric factors and the degree of stereoselective formation of our tetrahedral cages, which has been rarely explored, is examined in detail. In addition, distinct non-linear effects with these ligands in the formation of lanthanide tetrahedral cages are also reported, demonstrating that the differences in the formation behavior of **L1**–**L3** are dependent on the absolute configurations of the ligands.

## Results

### Ligand synthesis

**Fig. 1:**
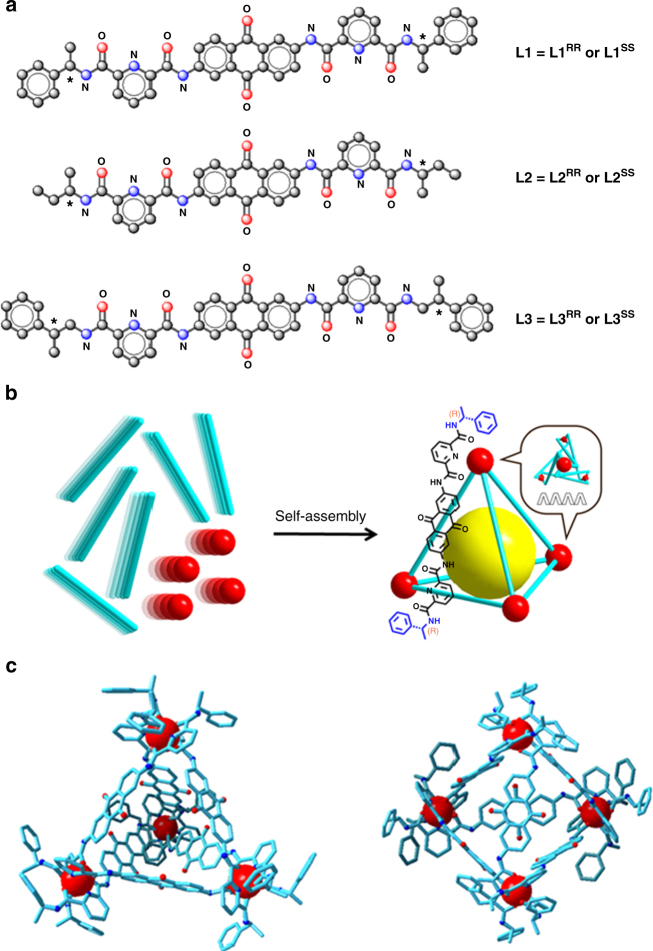
Self-assembly of tetrahedral cages. **a** Ligands (**L** = **L1**–**L3**) used for studying the stereoselective formation behavior of the tetrahedral cages. **b** A projecture of the stereoselective formation of the tetrahedral cage Λ,Λ,Λ,Λ-[Eu_4_(**L1** ^**RR**^)_6_](OTf)_12_]. Cavity for potential encapsulation of guest molecules is highlighted as a yellow spherical space. **c** Energy-minimized schematic representation of the tetrahedral cage in two different orientations

Previously, we have developed a system of stereoselective formation of lanthanide bimetallic triple helicates with chiral ligands in which chiral moieties of the ligands are connected with a linear rod-like benzidine-based biphenyl linker^31^. Based on these designs, we conceived different ligand building blocks to engineer supramolecular assemblies of a higher hierarchical order. The three pairs of chiral bis-tridentate ligands **L1**–**L3** are based on the diagonal 2,6-diaminoanthraquinone unit, (Fig. 1a), in which **L2** has a smaller point chirality relative to **L1**, and **L3** has an extended point chirality from the coordinating unit. Utilization of this type of linker for potential guest encapsulated-tetrahedral cage formation was first demonstrated with transition metals a few years ago;^32^ here we aim to engineer a similar topology of luminescent supramolecular assemblies with lanthanide ions: Ln_4_L_6_ tetrahedral cages (Fig. 1b, c).

The three ligands (**L1**–**L3**) were synthesized in two steps using general HATU peptide coupling reactions^33^ sourced from commercially available pyridyl dicarboxylic acid, chiral amines, and 2,6-diaminoanthraquinone. ^13^C NMR spectra of the ligands show a total of 19 aromatic and carbonyl carbon signals for **L1** and **L3**, and 14 signals for **L2**, indicating a dynamically average *C*_2_-symmetric nature of these ligands in solution.

### Synthesis and ESI-MS characterization of tetrahedral cages

The corresponding europium complexes, [Eu_4_(**L**)_6_](OTf)_12_ (**L** = **L1**–**L3**), were synthesized by reacting four equivalents of Eu(OTf)_3_ with six equivalents of ligands. Single crystals of [Eu_4_(**L1** ^**RR**^)_6_](OTf)_12_ was obtained by slow diffusion of ether into acetonitrile solution of the complex. Due to lack of high-angle diffraction, the structure cannot be completely solved. However, crystallographic results show a tetrahedral connection between the four europium ions, suggesting a tetrahedral cage topology (Supplementary Fig. 1 and Supplementary Table 1). All of the four europium ions are arranged in the same Λ-configurations, which are same as other reported supramolecular complexes with the same *R*-chiral moieties and similar pcam coordinating units^14, 34^. In our cage, each of the europium can be described to be distorted tricapped trigonal prismatic geometry. At each europium vertex, three pyridyl atoms, which located equatorial plane, are sandwiched by two trigonal faces which consisting of six oxygen atoms of amide units.

The tetrahedral stoichiometry of the species was further supported by high resolution ESI-MS analysis (Supplementary Figs. 2–7). Interestingly, these kind of tetrahedral cages possess a number of characteristics. For instance, [Eu_4_(**L1** ^**SS**^)_6_](OTf)_12_ exhibits three clusters of peaks which correspond to three different species with 4+, 5+, and 6+ charge states (Supplementary Fig. 2a). In each of the clusters, the peaks were found to correspond to the species with a consecutive loss of one triflate anion and one proton cation to account for the overall charge state. For example, in the group of 4+ charge, the peaks with *m*/*z* = 1564.2017, 1526.7125, 1489.2229, 1451.4837, and 1414.2432 can be assigned to molecular species of [{[Eu_4_(**L1** ^**SS**^)_6_](OTf)_*m*_}–(H)_*n*_]^4+^ with *m* = 4, *n* = 0; *m* = 5, *n* = 1; *m* = 6, *n* = 2; *m* = 7, *n* = 3 and *m* = 8, *n* = 4, respectively (Supplementary Fig. 2b). The assignments were verified by comparing to the corresponding isotopic distributions of the simulated and experimental results. Similar phenomena of subsequent loss of triflates and protons were also observed for the groups with 5+ and 6+ charges. The corresponding yttrium complexes, [Y_4_(**L**)_6_](OTf)_12_ (**L** = **L1**–**L3**), were also analyzed for comparison. From Electrospray ionization mass spectrometers (ESI-MS) analysis, this also verified the formation of tetrahedral cages with similar triflate(s) and proton(s) subtraction patterns when compare to the corresponding isostructural Eu counterparts (Supplementary Figs. 5–7).

### NMR characterization of tetrahedral cages

^1^H and ^13^C NMR experiments were performed to characterize the supramolecular tetrahedral cages. For [Eu_4_(**L1** ^**RR**^)_6_](OTf)_12_, the integrations are equivalent to the corresponding ligand in *C*_2_-symmetric nature (Supplementary Fig. 8). As all resonances are affected by slight paramagnetic broadening, the diagnosis of the presence of the total number of species was not confidently concluded. Even though ^1^H NMR experiments were performed at a different range of temperatures, e.g., over a span of 108 K, the resonances were still too broad to confidently verify the number of species present (Supplementary Fig. 9a). However by further analysis, a plot of *δ* vs. 1/*T* shows good linearity and implies the consistent presence of a single species in each temperature regime (Supplementary Fig. 9b)^35^. No obvious coalescence nor decomposition was observed upon increasing the temperature to 346 K showing that the cage is relatively thermally stable^32^. This hypothesis of the existence of a single species is also further supported by diffusion ordered NMR spectroscopy (DOSY) measurement where dominantly, only one diffusion coefficient (*D*) corresponding to the resonances of the complex was observed (Supplementary Fig. 10). Luckily, a clearer understanding of the presence of single species could be verified by ^13^C NMR, where only one set of resonances was observed (Supplementary Fig. 11). A total of 20 resonances match the *C*_2_-symmetric nature of the ligand, a result that indicates a highly symmetric environment as expected for the supramolecular tetrahedral cage structure (Supplementary Fig. 12). A diamagnetic counterpart, [Y_4_(**L1** ^**SS**^)_6_](OTf)_12_, was prepared for a more extensive study. For these Y_4_(**L1** ^**SS**^)_6_ cages, the ^1^H NMR signal clearly indicates that there is only a single species present in solution (Supplementary Fig. 13). The absence of exchange between the unbound and bound **L1** in these complexes also reveal the stability and integrity of these complexes on the NMR time scale.

For complexes based on **L2** and **L3**, a very different NMR observation was obtained compared with [Eu_4_(**L1**)_6_](OTf)_12_. Their corresponding ^1^H and ^13^C NMR resonances resolved into two sets of resonances (Supplementary Figs. 14 and 16). The ratios of ~1:1.08(2) for **L2** (0.93 and 2.59 ppm based, (Supplementary Fig. 14a and 15)) and ~1:1.21(5) for **L3** (6.09 and 6.19 ppm based, (Supplementary Fig. 16a and 17)) were found. The NMR shows splitting into two sets of resonances, indicating a high probability of the formation of two supramolecular species where isomerization are extremely slow in the NMR timescale. However, we postulate that these two species should possess a very similar supramolecular structure as shown by one dominant *D* value found in the DOSY measurement (Supplementary Fig. 18). In addition, the two species seem to be in some extent of dynamic motion as verified with variable temperature ^1^H NMR experiments. For [Eu_4_(**L2**)_6_](OTf)_12_, the two sets of resonances for aromatic protons (a–c and e–g) show coalescence at 267 and 283 K. However, no obvious coalescence can be observed for the rest of the protons (i, j, and l) even upon increasing temperature to 346 K (Supplementary Fig. 19). For [Eu_4_(**L3**)_6_](OTf)_12_, only pyridyl protons (e–g) show coalescence at 325 and 346 K when increasing in temperature. For the other aromatic protons (b–c), two set of resonances still could not be resolved even at very low temperature (238 K). For the aliphatic protons (i and j), no obvious coalescence in this span of temperature was observed (Supplementary Fig. 20). Absence or presence of coalescence at different temperatures may indicate different extent of dynamic motion occuring for different type of protons of ligands in the same cage. For each ligand, the respective diamagnetic counterparts, yttrium cages ([Y_4_(**L2/L3**)_6_](OTf)_12_), were examined which also exhibited two sets of resonances (Supplementary Fig. 21), suggesting the existence of two supramolecular species. The ratios of ~1: 1.15(5) for **L2** (0.86 and 0.55 ppm based, (Supplementary Fig. 21a)) and ~1: 1.06(4) for **L3** (0.93 and 1.13 ppm based, (Supplementary Fig. 21b)) were found.

For the above tetrahedral cages, concentration and solvent effects were also investigated. The results suggest that tetrahedral to helicate conversion which was observed in other similar tetrahedral cages^36^ cannot be observed (Supplementary Figs. 22–33).

### Chiral optical measurements

**Fig. 2:**
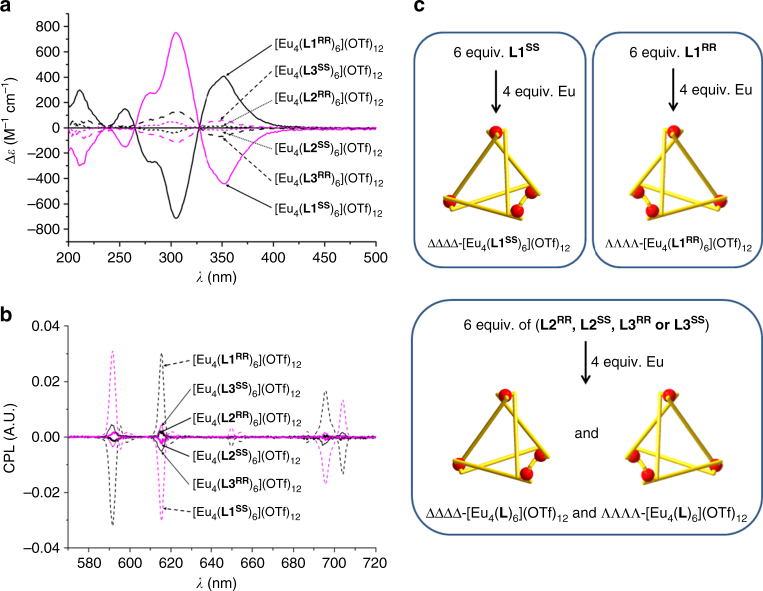
The chiral optical measurements. **a** Circular dichroism spectra of [Eu_4_(**L1**)_6_](OTf)_12_, [Eu_4_(**L2**)_6_](OTf)_12_, [Eu_4_(**L3**)_6_](OTf)_12_ in MeCN. The order of Cotton effect is [Eu_4_(**L1**)_6_](OTf)_12_>[Eu_4_(**L3**)_6_](OTf)_12_>[Eu_4_(**L2**)_6_](OTf)_12_. **b** Circular polarized emission spectra of [Eu_4_(**L1**)_6_](OTf)_12_ (1.07 × 10^−6^ M), [Eu_4_(**L2**)_6_](OTf)_12_ (1.16 × 10^−6^ M), [Eu_4_(**L3**)_6_](OTf)_12_ (1.11 × 10^−6^ M) in MeCN. The order of CPL intensity is [Eu_4_(**L1**)_6_](OTf)_12_>[Eu_4_(**L3**)_6_](OTf)_12_>[Eu_4_(**L2**)_6_](OTf)_12_. **c** Schematic representation of a plausible reason to explain the different extent of chiral optical intensity. **L1** leads to very stereoselective formation of either ΛΛΛΛ- or ΔΔΔΔ-isomer, whereas **L2** or **L3** form a mixture of ΛΛΛΛ- and ΔΔΔΔ-isomers with different ratios

Measurement of solution CD was performed for [Eu_4_(**L**)_6_](OTf)_12_ (**L** = **L1**–**L3)**. Similar to the NMR observation, the cage of **L1** also exhibited very different and distinct phenomena in the CD measurements when compared with the cages formed with **L2** and **L3**. In general, [Eu_4_(**L1** ^**SS**^)_6_](OTf)_12_ resulted in a very strong exciton coupling with peaks at 352, 306, 279 (shoulder), 255, and 211 nm (Fig. 2a). A predicted mirror image of the spectra was also observed for the opposite chiral isomer **L1** ^**RR**^, indicating their enantiomeric nature. In addition, the corresponding Y cages also resulted in CD spectra very similar to those of the Eu cages (Supplementary Fig. 34).

On the other hand, the other two cages, [Eu_4_(**L**)_6_](OTf)_12_ (**L** = **L2** or **L3**) resulted in significant attenuation of the Cotton effect when compared with [Eu_4_(**L1**)_6_](OTf)_12_ (Fig. 2a). Generally, the extent of signal depletion from **L2** [96(1)% (352 nm), 95(1)% (306 nm), 94(1)% (279 nm), and 91(5)% (211 nm)] was greater than that from **L3** [85(1)% (352 nm), 83(1)% (306 nm), 79(1)% (279 nm), and 84(4)% (211 nm)]. Signal attenuation was observed for the corresponding Y cages of **L2** or **L3** (Supplementary Fig. 34). The similarity in the CD phenomena between Eu and Y cages indicated that the supramolecular behavior of the tetrahedral cages was similar.

The enantiomeric nature of the supramolecular tetrahedral cages was also proved by the corresponding CPL spectra. CPL of supramolecular chiral f-tetrametallic tetrahedral cages has not been reported previously. For [Eu_4_(**L1**)_6_](OTf)_12_, mirroring CPL spectra were observed for the corresponding complexes of **L1** ^**RR**^ and **L1** ^**SS**^ isomers, confirming their enantiomeric property (Fig. 2b, Supplementary Fig. 35a). For the other two Eu cages with **L2** or **L3**, the CPL response decreased significantly with [Eu_4_(**L2**)_6_](OTf)_12_ exhibiting the weakest signals (Fig. 2b). This trend matches and supports the results derived from the CD studies. This observation can be attributed to the different overall screw sense of the different cages arising from the Eu ions contortion, which in turn is controlled by the stereoselective controlling ability of the ligand strands. This can be assumed as CPL reveals chiral information directly at the emitting Eu center and provides a better insight to the supramolecular chirality instead of CD data which only concerns the absorption of the chiral ligand. In terms of the degree of CPL response, [Eu_4_(**L1**)_6_](OTf)_12_ was found to have the two highest luminescent dissymmetry factors, *g*_lum_(591 nm) = –0.16(1) and +0.16(1) which is comparable in magnitude (–0.19 and +0.19) to the corresponding monomeric complexes with the same chiral group at the same ^5^D_0_ → ^7^F_1_ transition (Supplementary Fig. 35b(i); Supplementary Table 2)^37^. The *g*_lum_(704 nm) values for the ^5^D_0_ → ^7^F_4_ transition was found to be –0.16(2) and +0.16(1), which is lower than reported values (–0.24 and +0.24,) of the monomeric complexes. Very weak *g*_lum_(591.0 nm) was observed for [Eu_4_(**L3**)_6_](OTf)_12_ ( +0.04(2) and ─0.03(2)), whereas no reasonable dissymmetry factors were found for [Eu_4_(**L2**)_6_](OTf)_12_ (Supplementary Fig. 35b(ii–iii); Supplementary Table 2), affirming the extremely weak signals.

From these spectroscopic data, we can preliminarily conclude that only one diastereomer tetrahedral cage is formed from **L1** ^**RR/SS**^, whereas two diastereomers are formed with **L2** ^**RR/SS**^ or **L3** ^**RR/SS**^, resulting in the opposite CD and CPL signals and hence the weaker optical properties (Fig. 2c).

### Chiral amplification

**Fig. 3:**
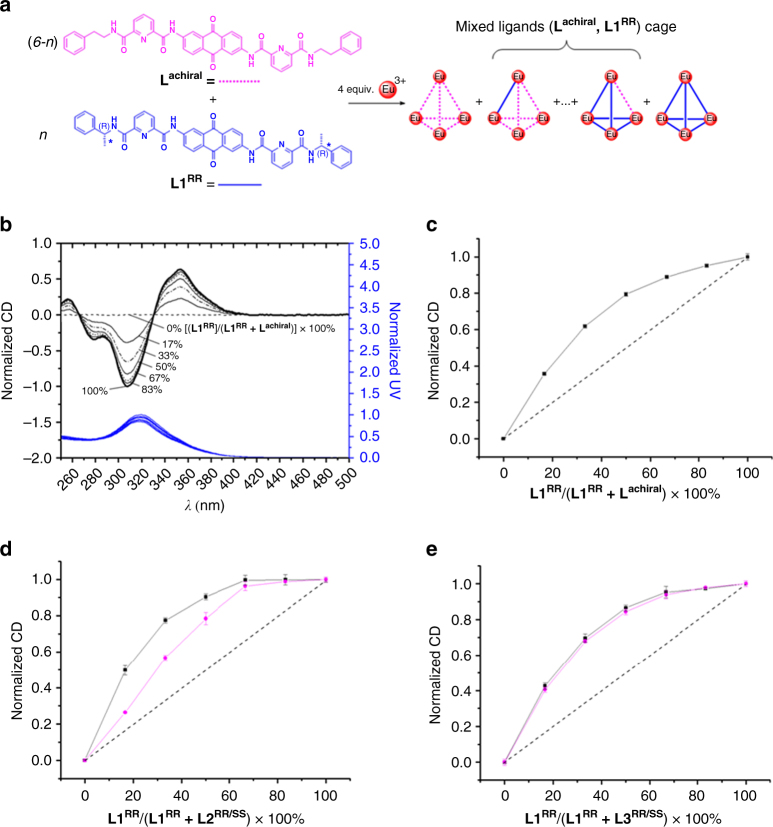
Chiral amplification experiments. The chiral amplification experiments of **L** ^**achiral**^, **L2** ^**RR/SS**^ or **L3** ^**RR/SS**^ (soldiers) were performed with **L1** ^**RR**^ (sergeant). **a** Schematic diagram of chiral amplification experiment of tetrahedral cage, [(**L1** ^**RR**^)_*n*_(**L** ^**achiral**^)_6─*n*_Eu_4_(OTf)_12_], (*n* = 0─6), using **L** ^**achiral**^ and **L1** ^**RR**^. **b** Normalized CD (top) and normalized UV–Vis absorption spectra (bottom) of the supramolecular tetrahedral cage [(**L1** ^**RR**^)_*n*_(**L** ^**achiral**^)_6─*n*_Eu_4_(OTf)_12_], (*n* = 0─6) in CH_2_Cl_2_/MeOH/MeCN (73:3:24, v/v/v) by maintaining 2.05 × 10^−5^ M of [(**L1** ^**RR**^)_*n*_(**L** ^**achiral**^)_6-*n*_]Eu_4_(OTf)_12_], (*n* = 0─6). **c** Plots of normalized CD intensities (354 nm) of the supramolecular cage [(**L1** ^**RR**^)_*n*_(**L** ^**achiral**^)_6─*n*_Eu_4_(OTf)_12_] (*n* = 0─6) as a function of % **L1** ^**RR**^. Error bars are standard errors of the means (s.e.m.). Performed in triplicate. **d** Plots of normalized CD intensities (354 nm) for [(**L1** ^**RR**^)_*n*_(**L2** ^**RR/SS**^)_6─*n*_Eu_4_(OTf)_12_] (*n* = 0─6), (square: **L2** ^**RR**^, circle: **L2** ^**SS**^) as a function of % **L1** ^**RR**^. Error bars are s.e.m. Performed in triplicate. **e** Plots of normalized CD intensities (354 nm) for [(**L1** ^**RR**^)_*n*_(**L3** ^**RR/SS**^)_6─*n*_Eu_4_(OTf)_12_] (*n* = 0─6), (square: **L3** ^**RR**^, circle: **L3** ^**SS**^) as a function of % **L1** ^**RR**^. Error bars are s.e.m. Performed in triplicate

Strong supramolecular stereoselective control of **L1** can be reflected in a series of nonlinear enhancing experiments (Fig. 3a, Supplementary Fig. 36). First of all, a pronounced deviation from linearity of CD intensity was observed for the supramolecular tetrahedral formation [(**L1** ^**RR**^)_*n*_(**L** ^**achiral**^)_6-*n*_Eu_4_(OTf)_12_], (*n* = 0–6) when there was a continuous subcomponent substitution of the achiral component(s) **L** ^**achiral**^, with the chiral component(s) **L1** ^**RR**^ (Fig. 3b, c). Due to the chiral inducing ability of **L1** ^**RR**^, an original racemic mixture of ΔΔΔΔ- and ΛΛΛΛ-[(**L1** ^**RR**^)_0_(**L** ^**achiral**^)_6_Eu_4_(OTf)_12_], probably swapping from ΔΔΔΔ- to ΔΔΔΛ-/ΔΔΛΛ-/ΔΛΛΛ-/ΛΛΛΛ- or ΛΛΛΛ- to ΛΛΛΔ-/ΛΛΔΔ-/ΛΔΔΔ-/ΔΔΔΔ-diastereomers cooperatively by substitution with an increasing amount of **L1** ^**RR**^, led to the enhancement of the nonlinear effect. The addition of around 17% of **L1** ^**RR**^ can induce ~40% of CD signal (354 nm) that arises from [(**L1** ^**RR**^)_6_(**L** ^**achiral**^)_0_Eu_4_(OTf)_12_].

For the tetrahedral cages of **L2** or **L3**, it is assumed that they behaved like achiral **L** ^**achiral**^ ligand as the CD and CPL observed were very weak. Due to these observed phenomena, it was therefore interesting to further explore whether **L1** can also facilitate prominent supramolecular inducing ability in the tetrahedral cages based on **L2** and **L3**. The following results showed that nonlinear enhancements were also observed for each of the supramolecular tetrahedral formation [(**L1** ^**RR**^)_*n*_(**L**)_6-*n*_Eu_4_(OTf)_12_] (**L** = **L2** ^**RR**^, **L2** ^**SS**^, **L3** ^**RR**^, or **L3** ^**SS**^; *n* = 0–6) between the strong supramolecular chiral inducing ligand **L1** ^**RR**^ and the weak chiral inducing ability of **L2** or **L3**. However, a slightly different extent of nonlinear effect was found in the formation of tetrahedral cages with the use of **L2** ^**RR**^ and **L2** ^**SS**^, with **L2** ^**SS**^ resulted in a lesser extent of nonlinear enhancement than **L2** ^**RR**^ (Fig. 3d, Supplementary Fig. 37a, b). On the other hand, **L3** ^**RR**^ and **L3** ^**SS**^ brought about a very similar extent of nonlinear effect (Fig. 3e, Supplementary Fig. 37c, d). We believe this is a unique way of correlating the extent of the chirality effect with the nonlinear enhancement using the soldier and sergeant model.

### Luminescence measurements

The luminescent nature of the newly synthesized Eu tetrahedral cages was investigated in solution states (Supplementary Figs. 38–43). Generally, all the Eu tetrahedral cages exhibited characteristic narrow line-like emission bands with peaks at 595, 616/619, 650, 688, and 696/705 nm corresponding to energy decay from the first excited state (^5^D_0_) to the ^7^F_*J*_ (*J* = 0, 1, 2, 3, 4) ground state multiplet–multiplet. The quantum yields (*ɸ*_rel_) of these supramolecular tetrahedral cages relative to quinine sulphate were determined to be 0.16(1)–0.17(1) for [Eu_4_(**L1**)_6_](OTf)_12_, 0.18(1) for [Eu_4_(**L2**)_6_](OTf)_12_ and [Eu_4_(**L3**)_6_](OTf)_12_ (Supplementary Table 3). Long excited state decays (*τ*  =~1.6 ms) in solution were measured at the ^5^D_0_→^7^F_2_ transition. Low temperature (77 K) emission of the [Gd_4_(**L1** ^**SS**^)_6_](OTf)_12_ complex was measured as well as the lifetime (0.3 μs), revealing the triplet state, which confirmed energy sensitization was via the “antenna” mechanism. (Supplementary Fig. 44).

### UV and NMR titrations

**Fig. 4:**
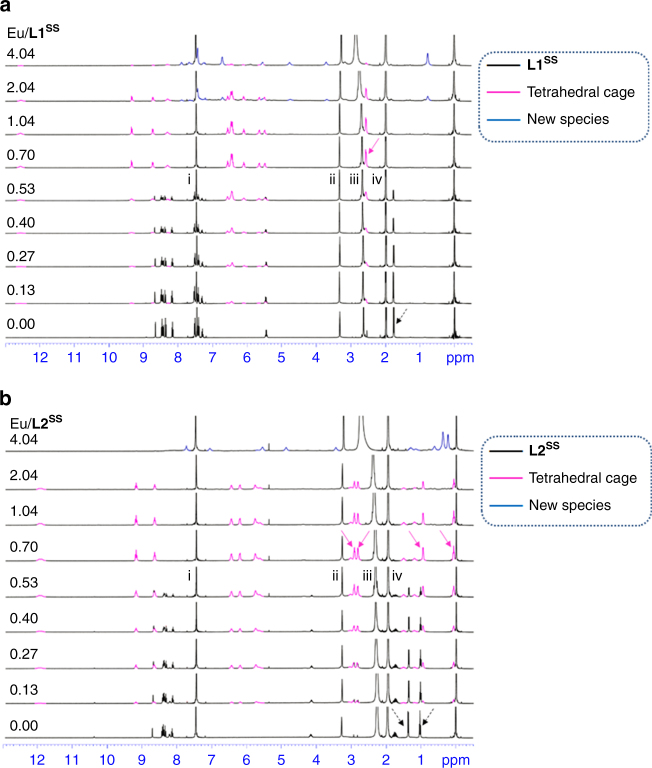
^1^H NMR titrations of L1 or L2 with Eu(OTf)_3_ to form tetrahedral cage. **a** Variation in ^1^H NMR spectra of titrating **L1** ^**SS**^ (1.23 × 10^−3^ M in 72:23:5, v/v/v, of CDCl_3_/CD_3_CN/CD_3_OD) with Eu(OTf)_3_ (0.0904 M in 67:33, v/v, of CD_3_CN/CD_3_OD) at 298 K. (Ligand is shown in black. Tetrahedral cage is shown in magenta. New species is shown in blue. Solid arrow and dash arrow indicate C*H*_*3*_- from the cage and ligand, respectively; peaks that are marked as i, ii, iii, iv are from the residual solvents of CHCl_3_, MeOH, H_2_O, and MeCN, respectively.) **b** Variation in ^1^H NMR spectra of titrating **L2** ^**SS**^ (1.37 × 10^−3^ M in 58:40:2, v/v/v, of CDCl_3_/CD_3_CN/CD_3_OD) with Eu(OTf)_3_ (0.136 M in CD_3_OD) at 298 K. (Ligand is shown in black. Tetrahedral cage is shown in magenta. New species is shown in blue. Solid arrow and dash arrow indicate C*H*_*3*_- from the cage and ligand, respectively; peaks that are marked as i, ii, iii, iv are from the residual solvents of CHCl_3_, MeOH, H_2_O, and MeCN, respectively)

The solution state formation behavior of these supramolecular tetrahedral cages was examined by titration experiments. First, UV–Vis titration of **L1** ^**RR**^ with Eu(OTf)_3_ in a solvent mixture of CHCl_3_/MeCN/MeOH (73:24:3, v/v/v) showed a smooth evolution of a new absorption band centering at 320 nm for the supramolecular complex and simultaneous progressive disappearance of absorption bands at 281, 315, and 353 nm for the ligand (Supplementary Fig. 45a). An end point ~0.69 was observed at four wavelengths in a plot showing the changes of molar absorptivity as a function of total equivalents of Eu(OTf)_3_ (Supplementary Fig. 45b), which is consistent to the formation of a [Eu_4_(**L1** ^**RR**^)_6_](OTf)_12_ species in solution. In the ^1^H NMR titration, upon addition of Eu(OTf)_3_, a new signal at 2.56 ppm in the aliphatic region progressively emerged at the expense of the signal at 1.75 ppm (C*H*_*3*_- of **L1** ^**SS**^) (Fig. 4a). After addition of 0.70 equiv. Eu(OTf)_3_, all the signals from the ligand disappeared, and one set of signals, which was most likely due to the stereoselective formation of one supramolecular species, was observed. Another new species, corresponding to a signal 0.76 ppm, evolved after further addition of Eu(OTf)_3_.

For the ligand **L2**, an end point of ~0.69 was observed with UV–Vis titrations (Supplementary Fig. 46). This projected the formation of a [Eu_4_(**L2** ^**SS**^)_6_](OTf)_12_ supramolecular species, a M_4_L_6_ cage was proposed. However, the species seemed to be more sensitive to a further addition of Eu(OTf)_3_ as observed with the absence of plateau immediately after the end point. ^1^H NMR titrations also showed a complete disappearance of signals from **L2** ^**SS**^ after addition of 0.70 equiv. Eu(OTf)_3_ (Fig. 4b). However, two sets of signals (0.07, 0.95, 2.84, and 2.94 ppm for C*H*_*3*_- of complex), in ~1:1.06 ratio, appeared at the expense of signals from **L2** ^**SS**^ (1.02 and 1.36 ppm for C*H*_*3*_- of **L2** ^**SS**^). Two closely related supramolecular species were likely to be formed stereoselectively in a little bias. With continuous addition of Eu(OTf)_3,_ a noticeable transformation to another new species was observed. For **L3**, no UV–Vis titration could be performed due to the limited solubility. However, some meaningful results were obtained from the ^1^H NMR titration of **L3** ^**SS**^ with Eu(OTf)_3_. Although no end point could be concluded, similar to **L2** ^**SS**^, the formation of two supramolecular species are proposed as shown by the presence of two sets of signals simultaneously (Supplementary Fig. 47). Another new complex was also observed when more Eu(OTf)_3_ was added, again formed at the expense of the original supramolecular species due to the decrease in the signals of the original species.

## Discussion

The significant differences in NMR, CD, and CPL observations arising from the tetrahedral cage formation with ligands **L1** vs. **L2** or **L3** can be associated to the different extent of stereoisomer formation. The resultant CD and CPL responses depend on the relative population of the two diastereomers, which in turn relies on the supramolecular stereoselective controlling ability of the ligand. Based on this hypothesis, a single tetrahedral isomer, either [Δ,Δ,Δ,Δ-Eu_4_(**L1** ^**RR/SS**^)_6_](OTf)_12_ or Λ,Λ,Λ,Λ-[Eu_4_(**L1** ^**RR/SS**^)_6_](OTf)_12_, is proposed to be the major form of **L1**; this is also justified by the single set of signals observed in ^13^C NMR and other NMR studies. The results also affirm the strong chiral inducing ability of this ligand is key to the formation of this pure stereoisomer supramolecular tetrahedral cage.

On the other hand, we have also shown that the tetrahedral cage formation process is extremely sensitive to any slight structural changes in the ligands. Significant deterioration effects on the diastereoselectivity were observed for **L2** and **L3**. When compared with **L1**, **L3** differs only in a slightly extended point chirality from the metal center, whereas **L2** incorporated a less sterically bulky chiral moiety and in both cases very weak CD and CPL responses were obtained. In each case, the two species observed based on NMR is proposed to be the co-existing diastereomeric mixtures of [Δ,Δ,Δ,Δ-Eu_4_(**L**)_6_](OTf)_12_ and Λ,Λ,Λ,Λ-[Eu_4_(**L**)_6_](OTf)_12_ (**L** = **L2** ^**RR/SS**^ or **L3** ^**RR/SS**^) with either a Λ or Δ helical wrapping of the binding strands at each europium ion. The two diastereomers induced opposite CD and CPL signals at each corresponding wavelength and resulted in a weaker overall intensity of the optical signals. Furthermore, we know that **L3** resulted in a slightly better supramolecular stereoselective control than **L2**, a phenomenon that was also reflected from the isomeric ratio in the NMR and a slightly stronger intensity in both CD and CPL for **L3**. Although preliminary, this suggests that steric effects have a greater influence on the diastereoselectivity for these europium tetrahedral cages than the location of point chirality.

Variable temperature ^1^H NMR studies also support the above hypothesis. Although different extents of dynamic motion for different protons such as antracenyl, pyridyl, aliphatic, and aromatic were found in [Eu_4_(**L2**)_6_](OTf)_12_ and [Eu_4_(**L3**)_6_](OTf)_12_, the dynamic motion for the pyridyl protons can be used to directly illustrate whether interconversion between Δ or Λ occurred at the Eu center. As a higher temperature was required for the pyridyl protons of [Eu_4_(**L3**)_6_](OTf)_12_ to achieve dynamic equilibrium/coalescence, this implies that **L3** possesses less rotational freedom to interconvert between Δ or Λ compared to **L2**.

Absolution configuration of the four europium ions can be inferred by CPL analysis. For [Eu_4_(**L**)_6_](OTf)_12_, a very similar CPL spectrum was observed when the corresponding monomeric complexes with the same chiral substituent were compared^37^. This implies that the absolution configurations (Δ/Λ) or helical twists (*P*/*M*) at the Eu ions of the supramolecular complexes are the same as those of the monomeric complexes^34, 38^. Hence, a Λ,Λ,Λ,Λ and Δ,Δ,Δ,Δ configuration is proposed for [Eu_4_(**L1** ^**RR**)^_6_](OTf)_12_ and [Eu_4_(**L1** ^**SS**^)_6_](OTf)_12_] respectively. A similar correlation of the CPL results associating to the absolute configuration has been successfully employed to Eu(III) triple stranded helicates in prior reports^28^.

For the chiral amplification experiment, a nonlinear effect of CD response with % of chiral ligand, **L1** ^**RR**^, was observed for the supramolecular tetrahedral formation [(**L1** ^**RR**^)_*n*_(**L** ^**achiral**^)_6─*n*_Eu_4_(OTf)_12_], (*n* = 0─6). A similar nonlinear enhancement of chiral response has been reported for subcomponent substitution in transitional metal-based M_4_L_6_ cage^19^ or hydrogen-bonded assembly^39^. However, this phenomenon is rarely known for lanthanide supramolecular complexes. This observation may imply that the presence of prominent cooperative stereochemical coupling between each stereogenic metal center may be the major factor leading to the nonlinear enhancement induced by **L1** ^**RR**^. For opposite isomers **L2** ^**RR**^ and **L2** ^**SS**^, this resulted in a different extent of nonlinearity, whereas **L3** ^**RR**^ and **L3** ^**SS**^ led to a similar nonlinear effect. It is postulated that these observations may be attributed to the different relative positions of asymmetric centers for **L2** and **L3**. In supramolecular cage formation with **L1** ^**RR**^ and **L2**, which possess the same relative position of asymmetric centers, their asymmetric methyl substituents may be located at a very close spatial arrangement from the nearby ligands at the same vertex, therefore leading to sensitivity to the (*R*)*-* and (*S*)-configuration for **L2**. On the contrary, the relative position of asymmetric centers for **L3** is one carbon away from the metal centers at each vertex compared with **L1** ^**RR**^ in the formation of the supramolecular cage. The difference in steric bulkiness from the chiral center may not be significant, hence resulting in similar nonlinear effect for **L3** ^**RR**^ or **L3** ^**SS**^.

Observations from UV–Vis absorption and NMR titrations suggest specific formation of one (for **L1**) or two very similar supramolecular species (for **L2**, **L3**) in solution state by titrating Eu(OTf)_3_ to a solution of each corresponding **L**. In UV–Vis titration, the simultaneous growth of a new absorption band and the decrease in the absorption for **L** suggest the existence of only the uncoordinated ligand and its supramolecular cage(s) (Supplementary Figs. 45 and 46), which corroborates with NMR titrations in which a single set of signals from **L1** (Fig. 4a) and two sets of chemical shifts from **L2** (Fig. 4b) and **L3** (Supplementary Figs. 47) were observed. The similarity of the two species observed for **L2** or **L3** is supported by the similar observation of one dominant diffusion coefficient from the DOSY analyses. In UV–Vis and NMR titrations, end-points of ~0.7 Eu/L were also observed, further supporting the topology of the tetrahedral cage, [Eu_4_(**L**)_6_](OTf)_12_.

The relative quantum yields (*ɸ*_rel_) of the supramolecular tetrahedral cages were found to be surprisingly high, ranging from 16(1) to 18(1)%. To the best of our knowledge, these values are the highest among the rarely reported *ɸ*_rel_ from supramolecular tetrahedral cages that comprise of a similar pyridyldicarbonyl antenna^9^. All the excited state decays are similar amongst themselves (*τ*=~1.6 ms) and to other supramolecular tetrahedron Eu compounds with a nine-coordinated geometry and a well-protected core^9^. The relatively long mono-exponential nature of the decays suggests the presence of a single non-polarized light emitting species in solution as well as efficient energy transfer for the europium complexes^40^.

In conclusion, we have successfully demonstrated the luminescent tetranuclear tetrahedral cages with different extent of CPL activities by employing several diagonal 2,6-diaminoanthraquinone linked chiral bis(pyridine-2,6-dicarboxamide) ligand building blocks (**L1**–**L3**) to assemble with europium ions. Interestingly, supramolecular diastereoselective formation behavior (ΔΔΔΔ-/ΛΛΛΛ- or ΔΔΔΔ- and ΛΛΛΛ-twisted conformations) was discovered to be extremely sensitive to any slight structural changes in the ligand. We also revealed that different isomers of the tetrahedral cage, from diastereoselective to non-diastereoselective formation, can be achieved by simply extending the point chirality slightly away from the metal centers or by decreasing the point chiral bulkiness to result in a significant deterioration of chiral information transfer. Moreover, cooperative stereochemical coupling between metal centers is observed to exhibit a prominent nonlinear enhancement effect. We demonstrated with our novel lanthanide cage that the position of point chirality plays a decisive role in this effect and opens up a window of possibilities for controlled topological formation and engineering of more elaborate supramolecular assemblies with functional properties. The results from this fundamental research provide insights for manipulating desired optical or magnetic properties for potential applications in materials, biological, sensing, and catalysis regimes.

## Methods

### General

Unless otherwise noted, all reagents were obtained commercially and without further purification. All moisture-sensitive compounds were manipulated using standard Schlenk line techniques. All moisture-sensitive reactions were conducted under a nitrogen atmosphere in glasswares that were oven-dried at 140 °C overnight prior to use. Anhydrous dimethylformamide and diisopropylamine were purchased from Acros and Aldrich, respectively. Other solvents were used as received without further purification. Merck silica gel 60 (70–230 mesh) was used for column chromatography. Compounds, (*R*)-6-(1-phenylethylcarbamoyl)picolinic acid (**1** ^**R**^), (*S*)-6-(1-phenylethylcarbamoyl) picolinic acid (**1** ^**S**^), (*R*)-6-(2-phenylpropylcarbamoyl)-picolinic acid (**3** ^**R**^), and (*S*)-6-(2-phenylpropyl carbamoyl)picolinic acid (**3** ^**S**^) are reported^31^. NMR spectra were recorded on a Bruker Ultrashield Advance Pro 400 or 600 MHz instrument and the chemical shifts were referenced internally to tetramethylsilane or the corresponding solvents residue in parts per million (ppm). UV–Visible absorption spectra were recorded with a HP UV-8453 spectrophotometer. The measurements were repeated three times with independent samples. HRMS was performed on an Agilent 6540 UHD Accurate-Mass Q-TOF LC/MS. HPLC analyses were performed on an Agilent 1200 series. Elemental analyses were performed on an Elementar Vario EL cube elemental analyzer. The optimized structure, by calculations using LUMPAC^41^ with a Sparkle/AM1 model, is shown in Fig. 1.

### Syntheses

All the new compounds were fully characterized. Experiment details and characterization are given in Supplementary Methods.

### Luminescence measurements

Single-photon luminescence spectra were recorded using an Edinburgh Instrument FLSP920 spectrophotometer that was equipped with a Xe900 continuous xenon lamp, a μF920 microsecond flashlamp anda single-photon counting Photomultiplier Tube. The excitation and emission spectra recorded on the FLSP920 were corrected with the correction file from the F900 software. Unless otherwise noted, all measurements were performed in triplicates.

### Quantum yield measurements

An ideal quantum yield standard should exhibit similar excitation and emission regions with the sample of interest, however, the Stokes shifts of commonly used organic fluorophores are smaller than the Richardson shifts^42^ of lanthanide complexes, therefore two standards matching the excitation and emission regions separately were used to cross-check the quantum yields of our complexes.

Standard 1:^43^ Quinine sulfate in 0.1 M sulfuric acid (*φ* = 0.577, *λ*_ex_ = 350 nm). Standard 2:^44^ Cs_3_[Eu(dpa)_3_] in 0.1 M Tris-HCl (*φ* = 0.240, *λ*_ex_ = 279 nm).

Absorption and emission spectra of the standard were measured at five or more absorbances within 0.1 in a 10 mm fluorescent quartz cuvette. A graph of integrated fluorescence intensity vs absorbance was plotted to give a straight line with gradient. The above procedure was repeated for the [Eu_4_(**L**)_6_](OTf)_12_. The related quantum yields were calculated according to the Eq. (1).

1 $${\Phi _{\rm{X}}} = {\Phi _{{\rm{ST}}}}\left( {{\rm{Gra}}{{\rm{d}}_{\rm{X}}}/{\rm{Gra}}{{\rm{d}}_{{\rm{ST}}}}} \right)\left( {\eta _{\rm{X}}^2/{\rm{ }}\eta _{{\rm{ST}}}^2} \right),$$

Where ST and X are standard sample and test sample, respectively. Grad is the gradient of the plots as stated above. *ƞ* is the refractive index of the solvent. Three independent samples were measured and the errors were then estimated.

### Circular dichroism

CD measurements were performed with a Jasco J-801 spectropolarimeter. Hellma quartz cuvettes (1 mm path length) were employed. All spectra were baselined subtracted with the blank solvent. Three independent samples of [Eu_4_(**L**)_6_](OTf)_12_ and [Y_4_(**L**)_6_](OTf)_12_ were measured.

### Circularly polarized luminescence

CPL measurements were conducted on a custombuilt spectrometer (in Durham University) with a laser light source (Energetiq EQ-99 LDLS with a spectral range 170–2100 nm) coupled to an Acton SP2150 monochromator (600 g/nm, 300 nm Blaze) that allows selection of the excitation wavelengths (6 nm FWHM band–pass). The collection of the emitted light was aided by a Lock–In Amplifier (Hinds Instruments Signaloc 2100) and Photoelastic Modulator (Hinds Instruments PEM-90 controller and Hinds Instruments Series II/FS2AA) with the use of a 90° angle set up and 1 cm path length quartz curette. The differentiated light was focused onto an Acton SP2150 monochromator (1200 g/nm, 500 nm Blaze) equipped with a high sensitivity cooled Photo Multiplier Tube [H10723-20 Extended red-multialkali PMT based photosensor (5 V, cooled to 4°C)]. Spectra were recorded in the range of 570–720 nm (0.5 nm spectral intervals) using a five spectral average sequence and 500 μs integration time. Three independent samples of [Eu_4_(**L**)_6_](OTf)_12_ were measured.

### Chiral amplification

The stock solutions of **L** ^**achiral**^ (0.000123 M) and **L1** ^**RR**^ (0.000123 M) (solution **B**) were prepared in a solvent mixture of CH_2_Cl_2_/MeOH/MeCN = 73:3:24. The stock solution of Eu(OTf)_3_ (0.00659 M) was prepared in MeOH. For each experiment, a total volume of 8 ml solution was mixed from solution **L** ^**achiral**^ and solution **L1** ^**RR**^ to contribute one data point. There were a total of seven data points according to the formula of [**L1** ^**RR**^/(**L1** ^**RR**^ + **L** ^**achiral**^) × 100%], where **L1** ^**RR**^ varied from 0 to 6 and **L1** ^**RR**^ + **L** ^**achiral**^ = 6, for each set of complete experiments. To these seven sets of solution, 0.1 mL of the stock Eu(OTf)_3_ was added and then mixed thoroughly. An aliquot of 1.5 mL of each solution was filled in an NMR tube. The tubes were sealed with septa and then allowed to stand at a 60 °C water bath for 24 h. After cooling at room temperature for 2 h, the solutions were checked by CD spectroscopy directly without any further dilution. In the cases of using **L2** ^**RR**^, **L2** ^**SS**^, **L3** ^**RR**^, or **L3** ^**SS**^ to replace the role of **L** ^**achiral**^, the same method was employed. For the enantiomers of **L2**, a longer reaction time (36 h) was required in order to obtain a more reliable result. **L3** was used as a suspension. The solution became clear after addition of Eu(OTf)_3_. For each set of complete experiments, triplicate measurements were performed to estimate the error.

### Single crystal X-ray diffractions

Data for [Eu_4_(**L1** ^**RR**^)_6_](OTf)_12_ were collected on beamline 11.3.1 at the Advanced Light Source, using a Bruker D8 diffractometer equipped with a PHOTONII CPAD detector operating in shutterless mode. The crystal was coated in protective oil prior to being mounted on a MiTeGen kapton micromount and placed under a nitrogen stream at 270(2)  K provided by an Oxford Cryostream 800 Plus low temperature apparatus. Diffraction data were collected using synchrotron radiation monochromated using silicon(111) to a wavelength of 0.7749(1) Å. An approximate full-sphere of data was collected using a combination of phi and omega scans. The structures were solved by intrinsic phasing (SHELXT)^45^ and refined by full-matrix least squares on *F* ^2^ (SHELXL-2014)^46^. Hydrogen atoms were geometrically calculated and refined as riding atoms. The thermal ellipsoids of the majority of the pendant ethylbenzene groups suggest that these groups are disordered, however only one of these groups in [Eu_4_(**L1** ^**RR**^)_6_](OTf)_12_ could be successfully modeled over multiple orientations, despite many attempts to resolve this disorder. Atoms belonging to three of the four pendant groups in each cluster have been given a common isotropic thermal displacement parameter. Due to the poorly diffracting nature of the crystals, the data to parameter ratio was too low to refine the carbon, nitrogen and oxygen atoms of the ligands with anisotropic thermal displacement parameters, so only the metal centers have been refined anisotropically. Some restraints have been placed on the bond lengths and angles of the bridging ligand. Although many attempts were made to model the triflate anions, these were found to be disordered within the crystals and could not be successfully modeled. The oxygen and sulfur atoms of these anions have been assigned, however, only one complete partial occupancy triflate anion could be modeled for each compound. An analysis of the electronic contribution of the disordered solvent and anions was performed using the SQUEEZE routine^47^ within PLATON^48^, and showed for both compounds that there was sufficient residual electron density to account for all necessary triflate anions plus some disordered solvent. The model was not refined against this ‘SQUEEZEd’ data. Crystal data for [Eu_4_(**L1** ^**RR**^)_6_](OTf)_12_: Space group *R*32, *a* = *b* = 29.2680(16) Å, *c* = 70.713(5) Å, *V* = 52458(7) Å^3^, *Z* = 6, *T* = 270(2). All non-metal atoms were refined with isotropic thermal displacement parameters on 7108 independent reflections (*R*_int_ = 0.0625) converged at residual *wR*_2_ = 0.3795 for all data. *R*_1_ = 0.1364 [*I* ≥ 2*σ*(*I*)] with goodness of fit on *F* ^2 = ^1.891.

### Data availability

The X-ray crystallographic data has been deposited at the Cambridge Crystallographic Data Centre with deposition number CCDC-1548073. This data can be obtained free of charge via www.ccdc.cam.ac.uk/conts/retrieving.html. All other data supporting the finding in this study are available within the article and its Supplementary Information, as well as from the authors upon reasonable request.

## Electronic supplementary material

Supplementary information
