# Supplementary material for: Chiral transcription in self-assembled tetrahedral Eu4L6 chiral cages displaying sizable circularly polarized luminescence
Source: Nat Commun. 2017 Oct 24;8:1128. doi: 10.1038/s41467-017-01025-1 (PMC5783948; doi:10.1038/s41467-017-01025-1)
Supplement: Supplementary file 1 — Supplementary information [file 41467_2017_1025_MOESM1_ESM.pdf]

Supplementary Figures and Supplementary Tables

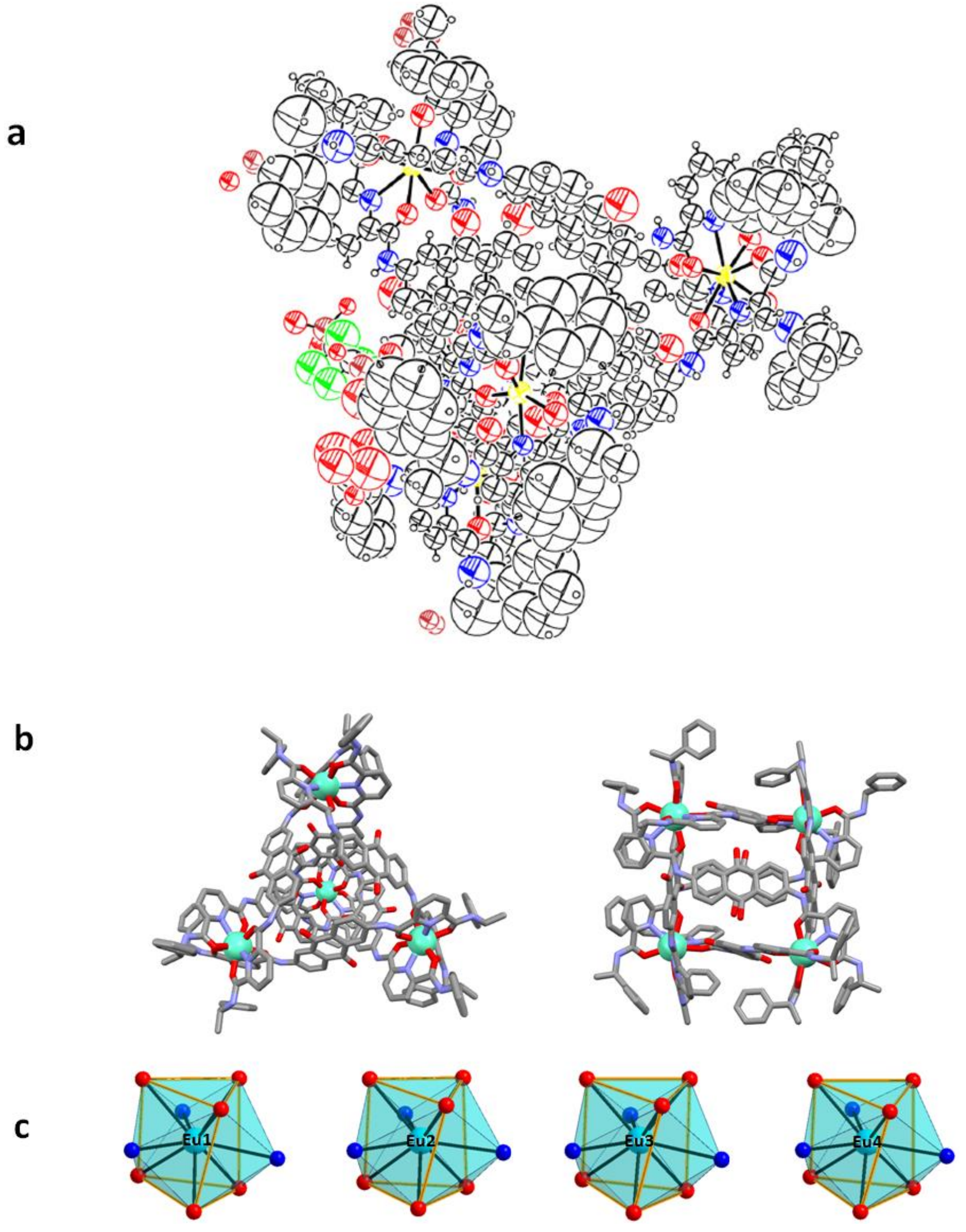

Supplementary Figure 1| Crystal structure of [Eu_4(L1^{RR})$_6$](OTf)$_{12}$. (a) Ortep-drawing for the [Eu_4(**L1**^{RR})$_6$](OTf)$_{12}$ at 50% probability level. (b) X-ray crystal structure of [Eu_4(**L1**^{RR})$_6$](OTf)$_{12}$ in two different orientation. Eu: cyan, C: grey, O: red, N: blue. (c) The four europium metal centers have same tricapped trigonal prism geometries.

They have same Λ absolute configuration.

Supplementary Table 1.

Crystal data and refinement of the complex **$[Eu_4(L1^{RR})_6](OTf)_{12}$**.

Compounds	$[Eu_4(\mathbf{L1}^{RR})_6](OTf)_{12}$
Empirical formula	$C_{276}H_{204}Eu_4F_{36}N_{36}O_72S_{12}$
Formula weight	6853.30
Temperature/K	270(2)
Crystal system	Trigonal
Space group	*R*32
a/Å	29.2680(16)
b/Å	29.2680(16)
c/Å	70.713(5)
α/°	90
β/°	90
γ/°	120
Volume/Å^3	52458(7)
Z	6
ρ_{calc}g/cm^3	1.302
μ/mm^{-1}	1.085
F(000)	20736
Crystal size/mm^3	0.120 x 0.110 x 0.005
Radiation	λ = 0.7749(1) Å
2Θ range for data collection/°	0.930 to 18.865
Index ranges	$-24 \le h \le 24, -24 \le k \le 24, -58 \le l \le 58$
Reflections collected	111319
Independent reflections	7108 [R(int) = 0.0625]
Data/restraints/parameters	7108 / 113 / 436
Goodness-of-fit on F^2	1.891
Final R indexes [I>=2σ (I)]	R_1 = 0.1364, wR_2 = 0.3642
Final R indexes [all data]	R_1 = 0.1434, wR_2 = 0.3795
Largest diff. peak/hole / e Å^{-3}	1.629/-0.892
Flack parameter	0.078(8)

a

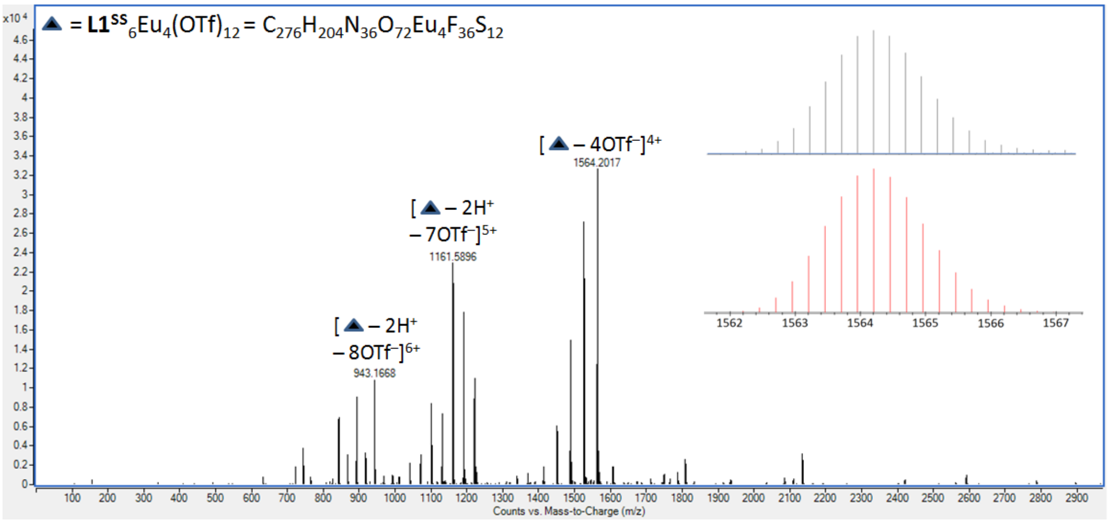

b

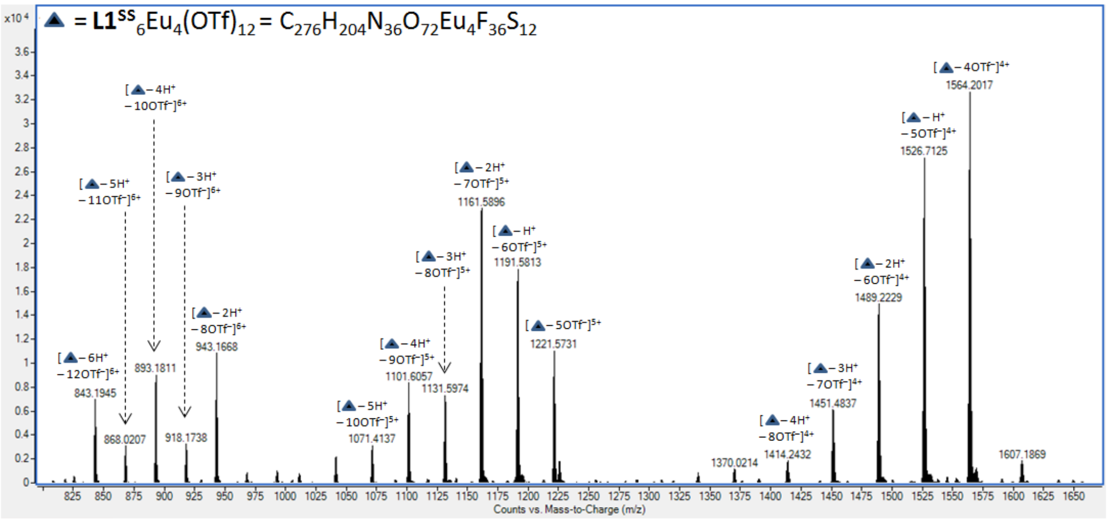

Supplementary Figure 2| ESI-MS of tetrahedral cages of [Eu_4(**L1**^{SS})$_6$]$(OTf)_{12}$. (**a**) The peak can be assigned to a tetracation of tetrahedral cage. Simulated m/z for [tetrahedron – 4OTf$^-$] is 1564.2071(100%), Experimental found m/z is 1564.2017(100%). Inset showing the experimental (upper) and calculated (lower) isotopic patterns. (**b**) Expanded region of the mass spectrum to show the possible assignments of the corresponding prominent peaks.

a

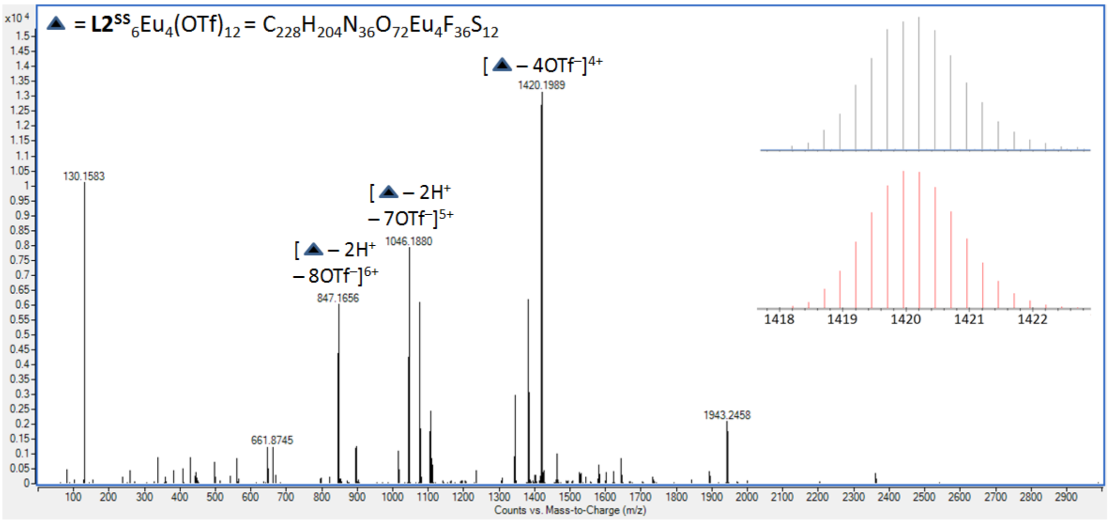

b

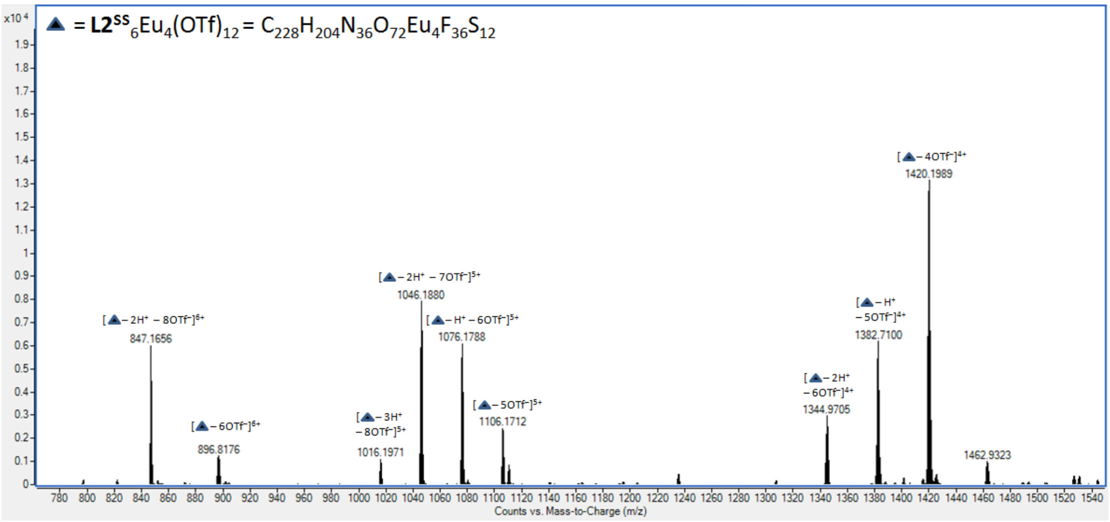

Supplementary Figure 3| ESI-MS of tetrahedral cages of [Eu$_4$(**L2**^{SS})$_6$](OTf)$_{12}$. (**a**) The peak can be assigned to a tetracation of tetrahedral cage. Simulated m/z for [tetrahedron – 4OTf$^-$] is 1420.2069(98.2%), Experimental found m/z is 1420.1989 (100%). Inset showing the experimental (upper) and calculated (lower) isotopic patterns. (b) Expanded region of the mass spectrum to show the possible assignment of the corresponding prominent peaks.

a

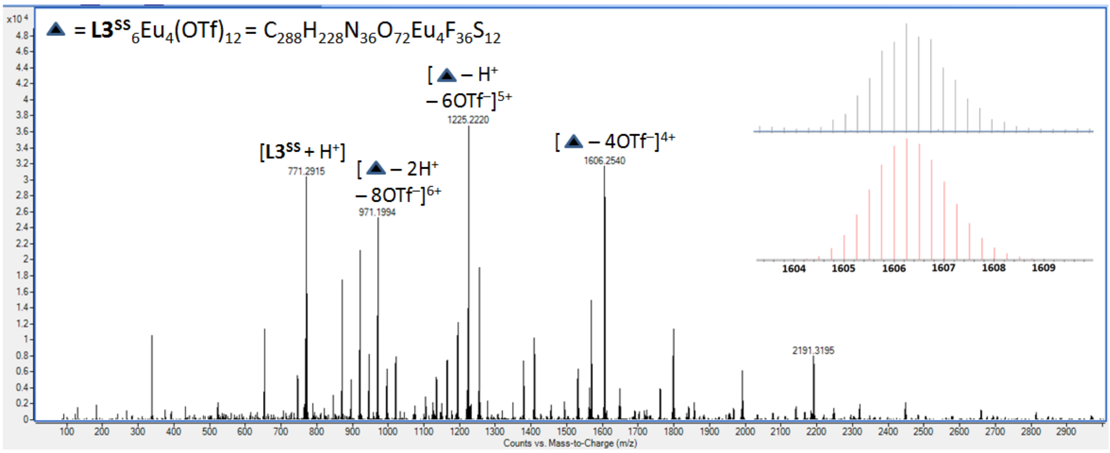

b

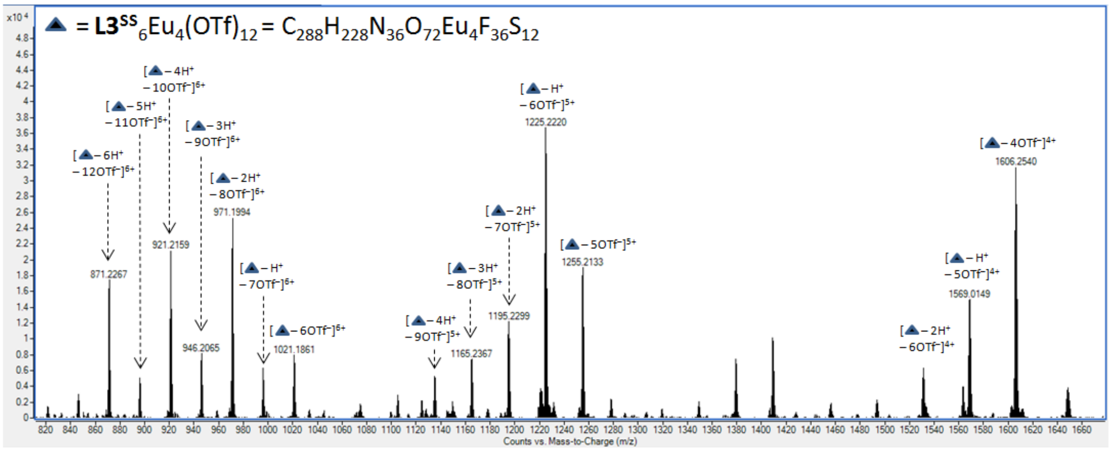

Supplementary Figure 4 | ESI-MS of tetrahedral cages of [Eu_4(**L3**^{SS})$_6$]$(OTf)_{12}$. (**a**) The peak can be assigned to a tetracation of tetrahedral cage. Simulated m/z for [tetrahedron – 4OTf$^-$] is 1606.2542(100%), Experimental found m/z is 1606.2540(100%). Inset showing the experimental (upper) and calculated (lower) isotopic patterns. (b) Expanded region of the mass spectrum to show the possible assignment of the corresponding prominent peaks.

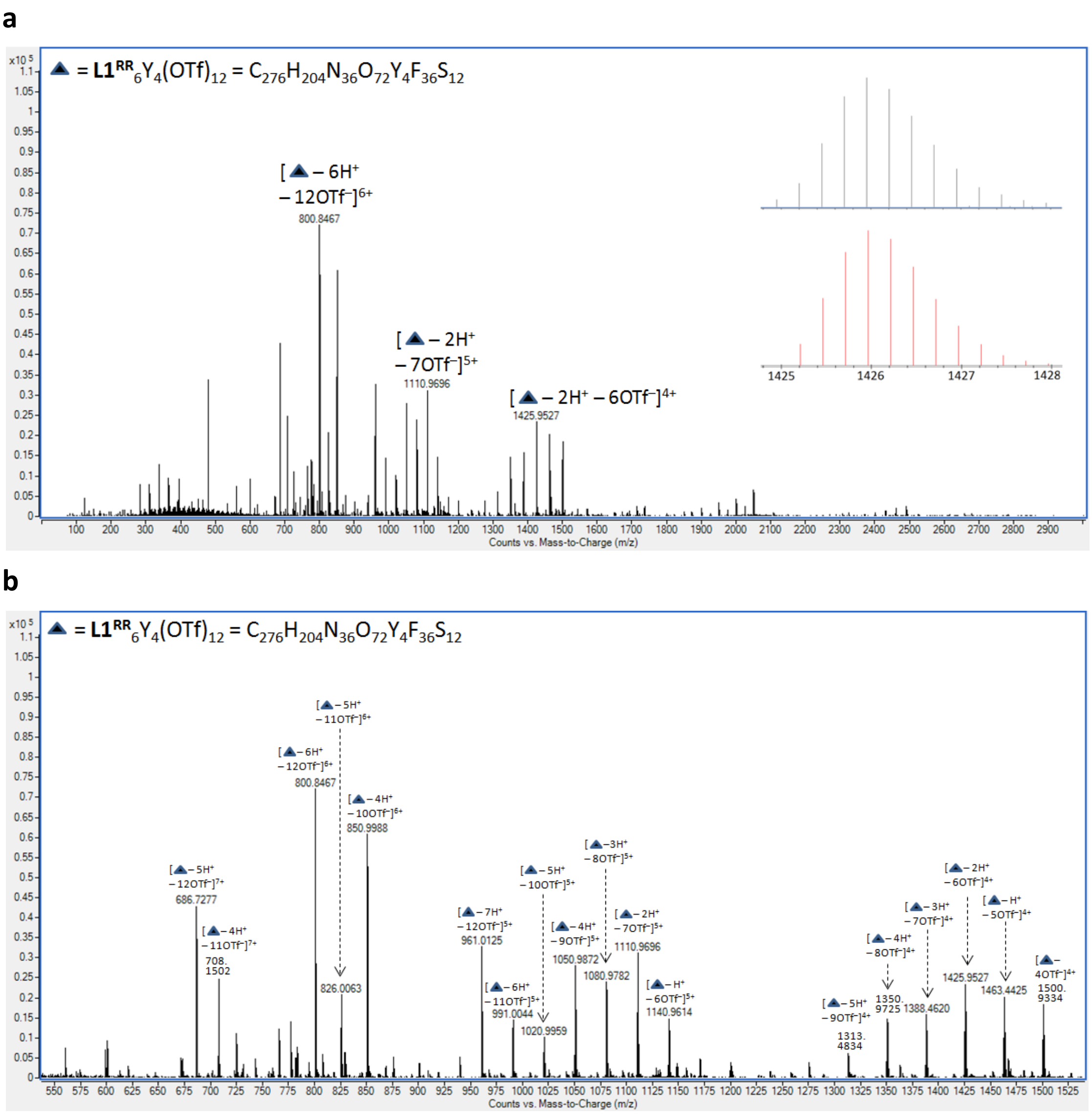

Supplementary Figure 5| ESI-MS of tetrahedral cages of $[Y_4(\mathbf{L1}^{RR})_6](OTf)_{12}$. (**a**) The peak can be assigned to a tetracation of tetrahedral cage. Simulated m/z for [tetrahedron – $2H^+$ – $6OTf^-$] is 1425.9623(100%), Experimental found m/z is 1425.9527(100%). Inset showing the experimental (upper) and calculated (lower) isotopic patterns. (**b**) Expanded region of the mass spectrum to show the possible assignments of the corresponding prominent peaks.

a

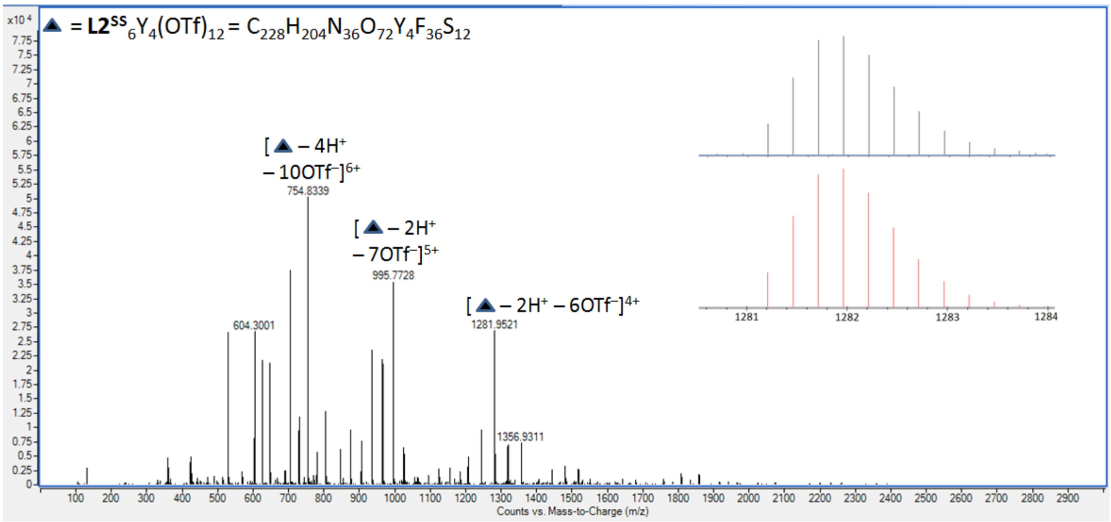

b

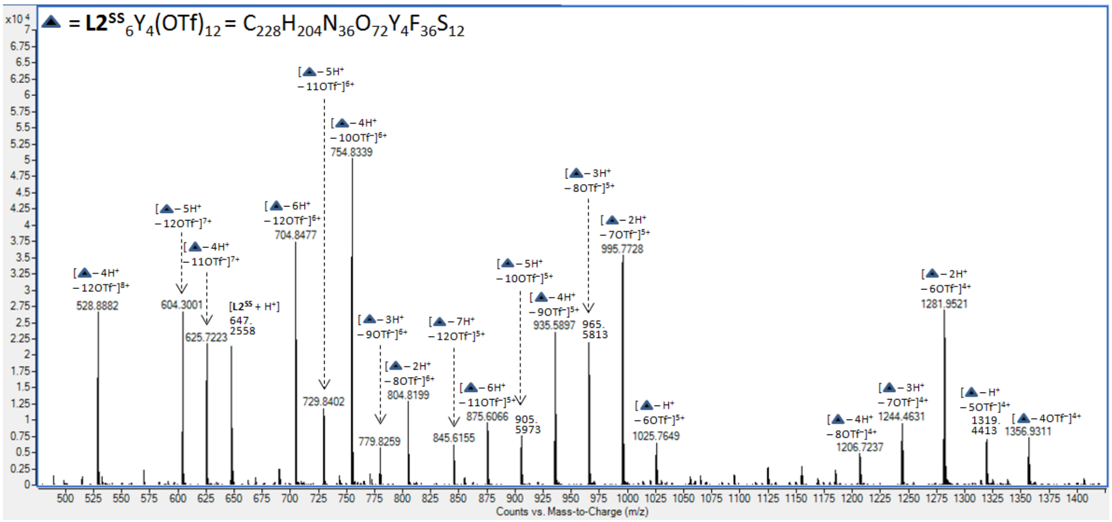

Supplementary Figure 6| ESI-MS of tetrahedral cages of $[Y_4(\mathbf{L2}^{SS})_6](OTf)_{12}$. (**a**) The peak can be assigned to a tetracation of tetrahedral cage. Simulated m/z for [tetrahedron – $2H^+$ – $6OTf^-$] is 1281.9622(100%), Experimental found m/z is 1281.9521(100%). Inset showing the experimental (upper) and calculated (lower) isotopic patterns. (**b**) Expanded region of the mass spectrum to show the possible assignments of the corresponding prominent peaks.

a

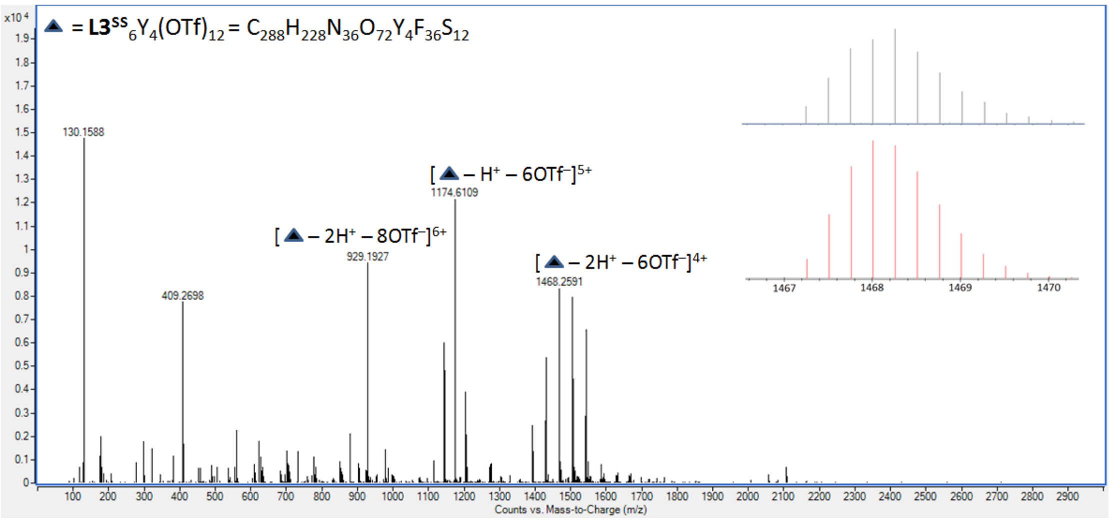

b

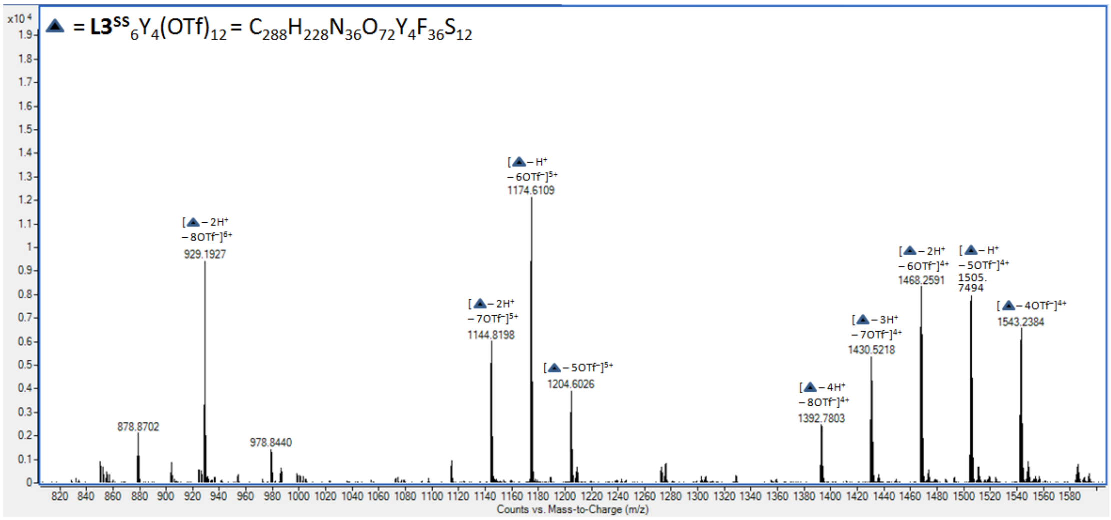

Supplementary Figure 7| ESI-MS of tetrahedral cages of $[Y_4(\mathbf{L3}^{SS})_6](OTf)_{12}$. (**a**) The peak can be assigned to a tetracation of tetrahedral cage. Simulated m/z for [tetrahedron – $2H^+$ – $6OTf^-$] is 1468.2598(96.48%), Experimental found m/z is 1468.2593(100%). Inset showing the experimental (upper) and calculated (lower) isotopic patterns. (**b**) Expanded region of the mass spectrum to show the possible assignments of the corresponding prominent peaks.

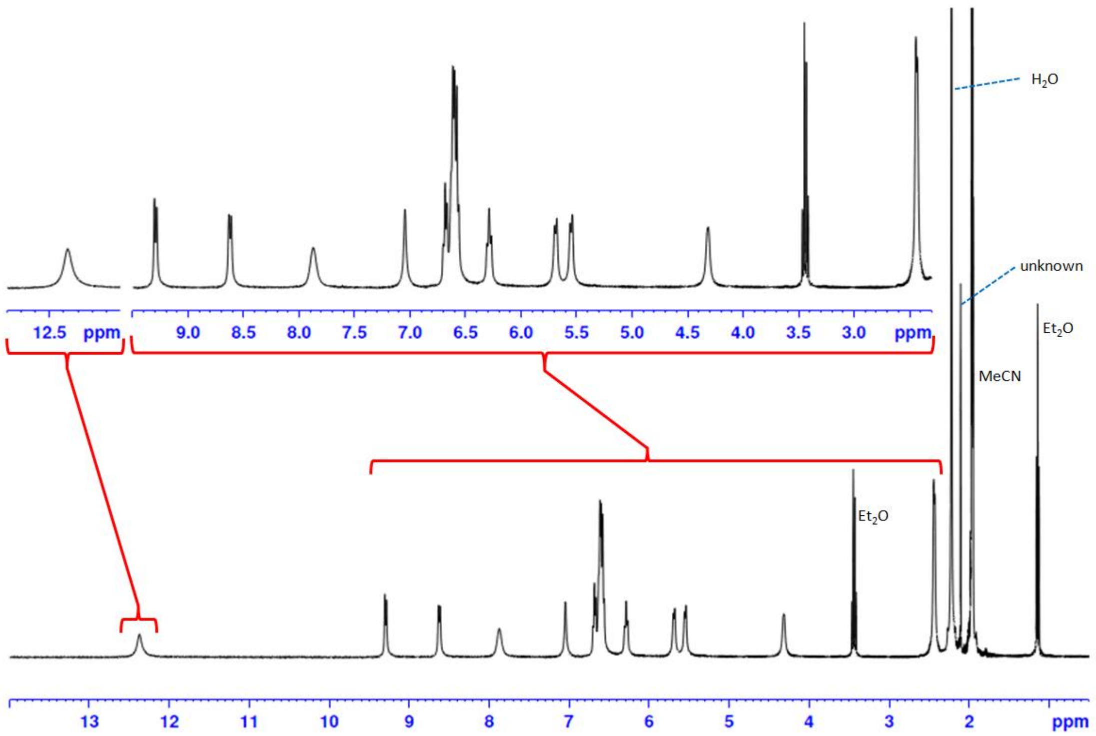

Supplementary Figure 8 | ^{1}H NMR (400 MHz, CD_3CN, 298 K) spectrum of $[Eu_4(L1^{RR})_6](OTf)_{12}$. The insets are the expanded regions as indicated.

a

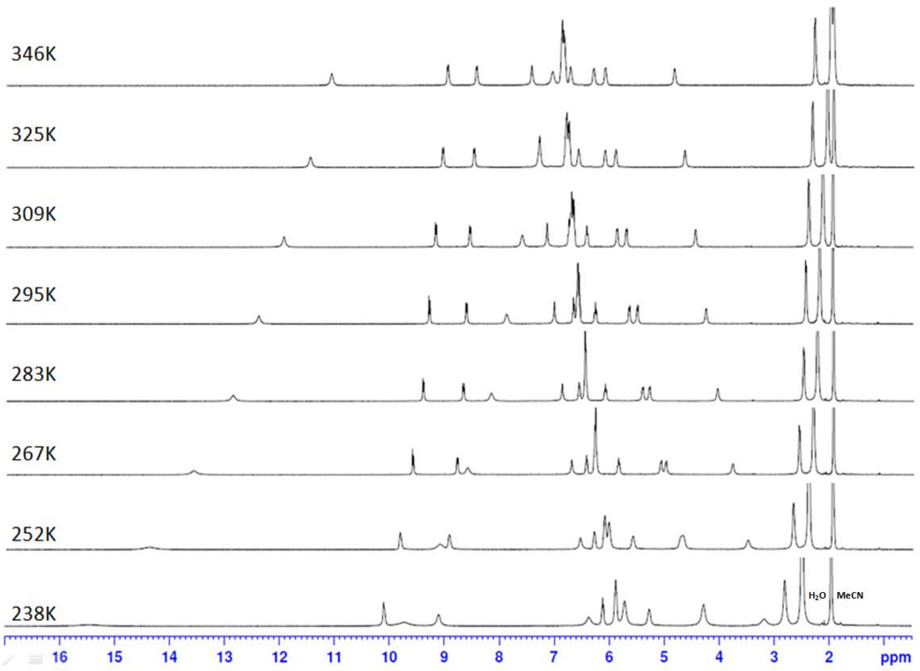

346K
325K
309K
295K
283K
267K
252K
238K
H₂O
MeCN
16
15
14
13
12
11
10
9
8
7
6
5
4
3
2
ppm

b

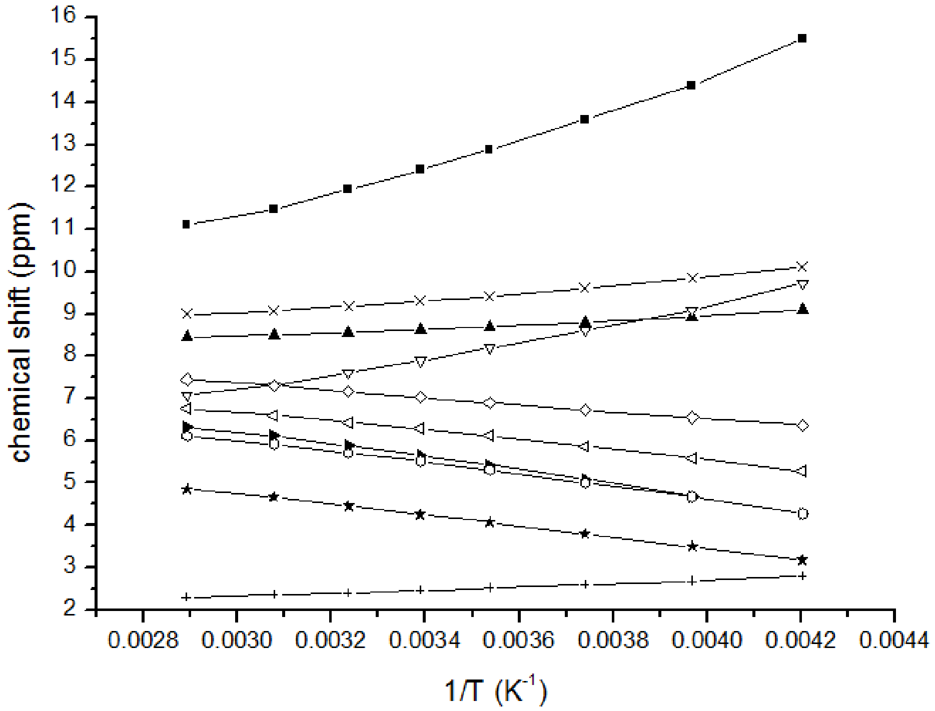

chemical shift (ppm)
1/T (K⁻¹)
16
15
14
13
12
11
10
9
8
7
6
5
4
3
2
0.0028
0.0030
0.0032
0.0034
0.0036
0.0038
0.0040
0.0042
0.0044

Supplementary Figure 9| Temperature profile (varied from 238 K to 346 K) of ^1H NMR spectra of $[Eu_4(L1^{SS})_6](OTf)_{12}$. (**a**) A stack of NMR spectra showing downfield and upfield shifts of resonances. (**b**) A plot of ^1H NMR chemical shifts versus 1/T showing linearity relationship between chemical shifts and inversed temperature.

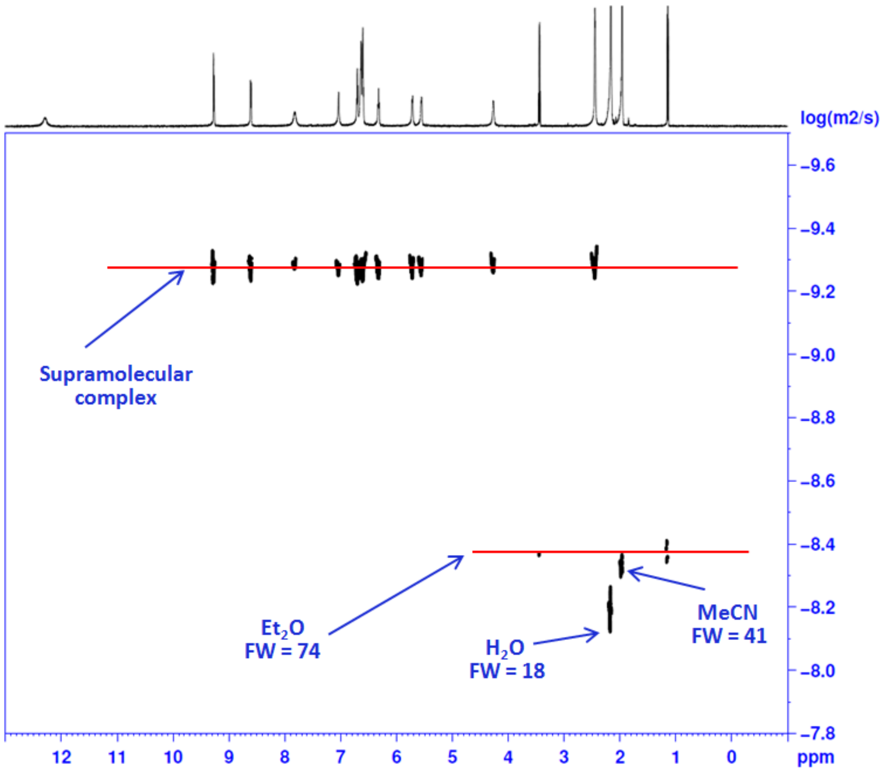

Supplementary Figure 10| DOSY spectrum of $[Eu_4(L1^{RR})_6](OTf)_{12}$ in CD_3CN at 298K. Formation of one supramolecular species is proposed.

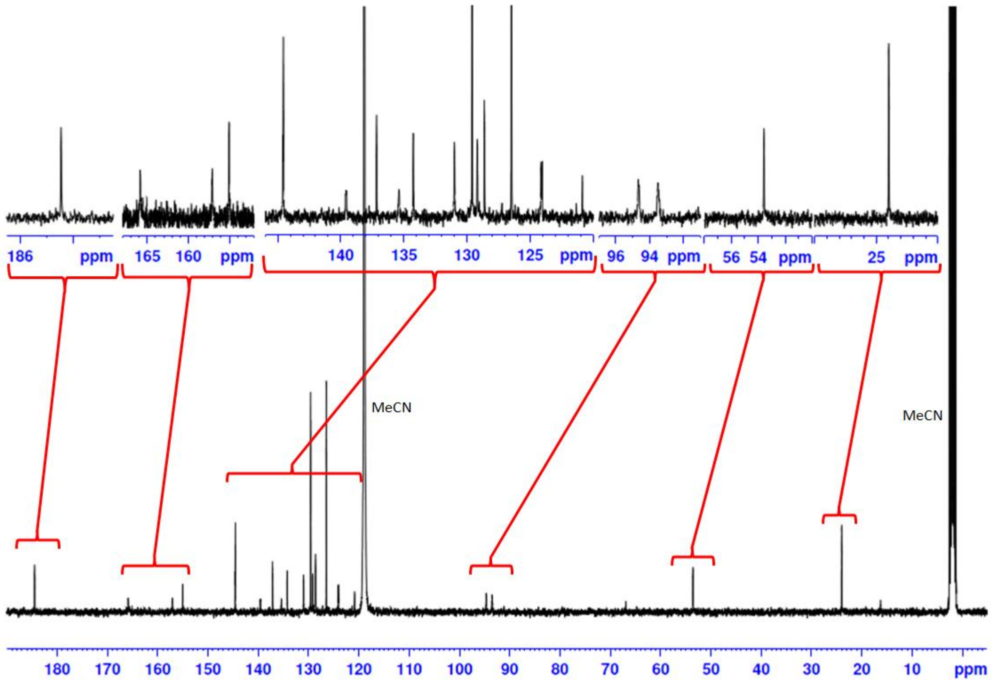

Supplementary Figure 11 | ^{13}C NMR (100.6 MHz, 296 K) spectrum of $[Eu_4(L1^{SS})_6](OTf)_{12}$ (CD_3CN). The insets are the expanded regions as indicated. Majorly one set of signal can be observed. Formation of one supramolecular species is proposed.

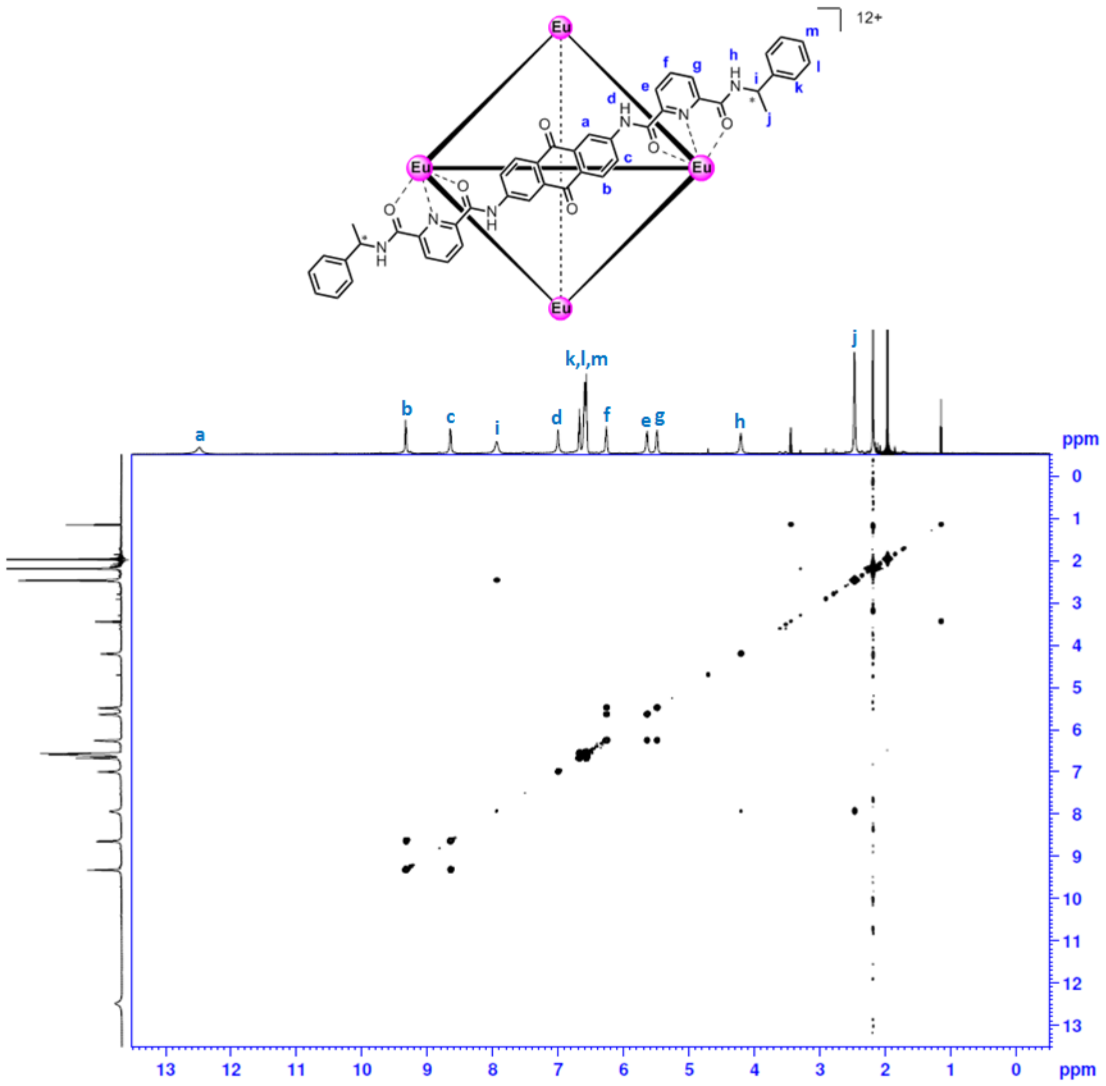

Supplementary Figure 12| ^{1}H–^{1}H COSY NMR (400 MHz, CD_3CN, 296 K) spectrum of $[Eu_4(L1^{RR})_6](OTf)_{12}$.

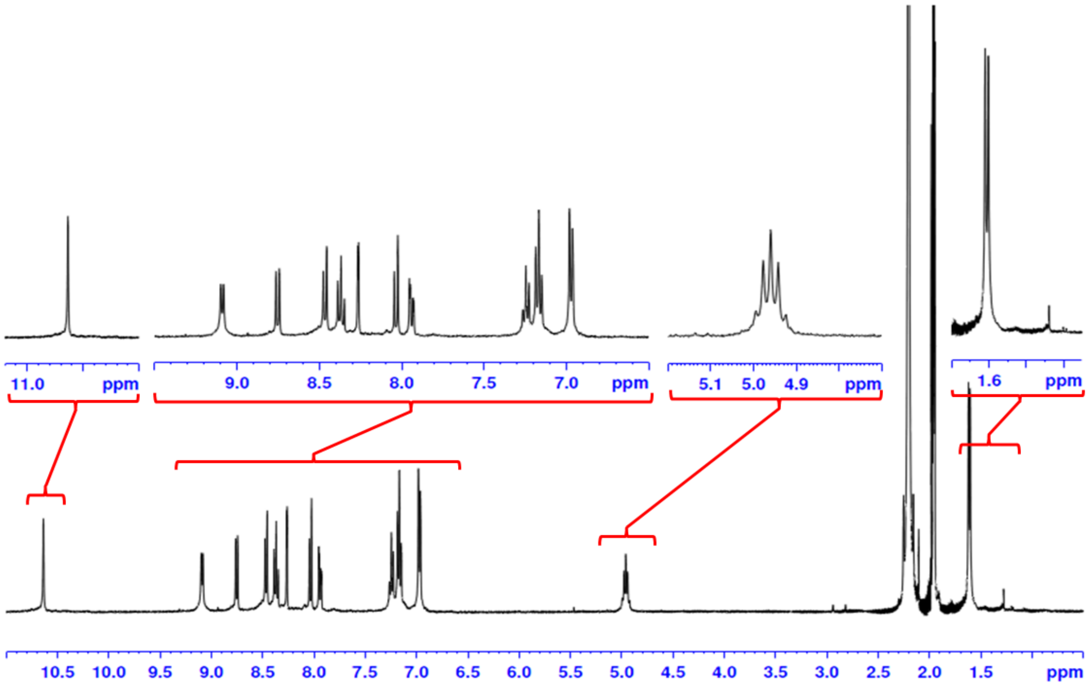

Supplementary Figure 13 | ^{1}H NMR (400 MHz, CD_3CN, 298 K) spectrum of $[Y_4(L1^{SS})_6](OTf)_{12}$. The insets are the expanded regions as indicated. Formation of one supramolecular species is proposed.

a

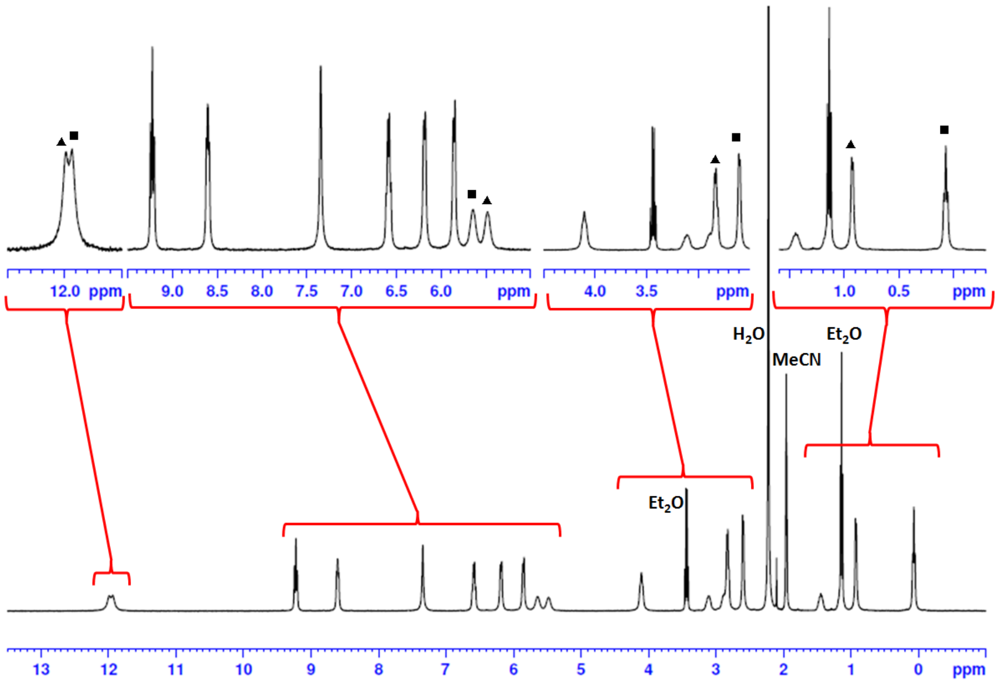

b

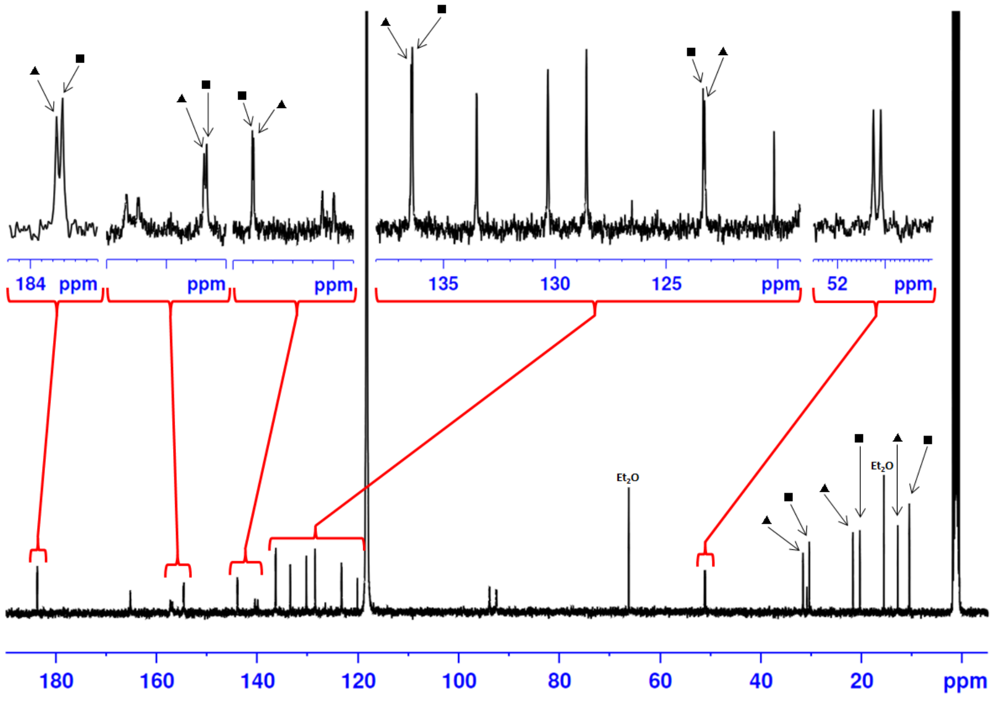

Supplementary Figure 14| ^{1}H NMR and ^{13}C NMR spectra of [$Eu_4(L2^{RR})_6$]$(OTf)_{12}$. (**a**) ^{1}H NMR (400 MHz, CD_3CN, 298 K). The insets are the expanded regions as indicated. ■ and ▲ represent for the major and minor species, respectively. (**b**) ^{13}C NMR (100.6

MHz, CD_3CN, 298 K). ■ and ▲ represent for the major and minor species, respectively.

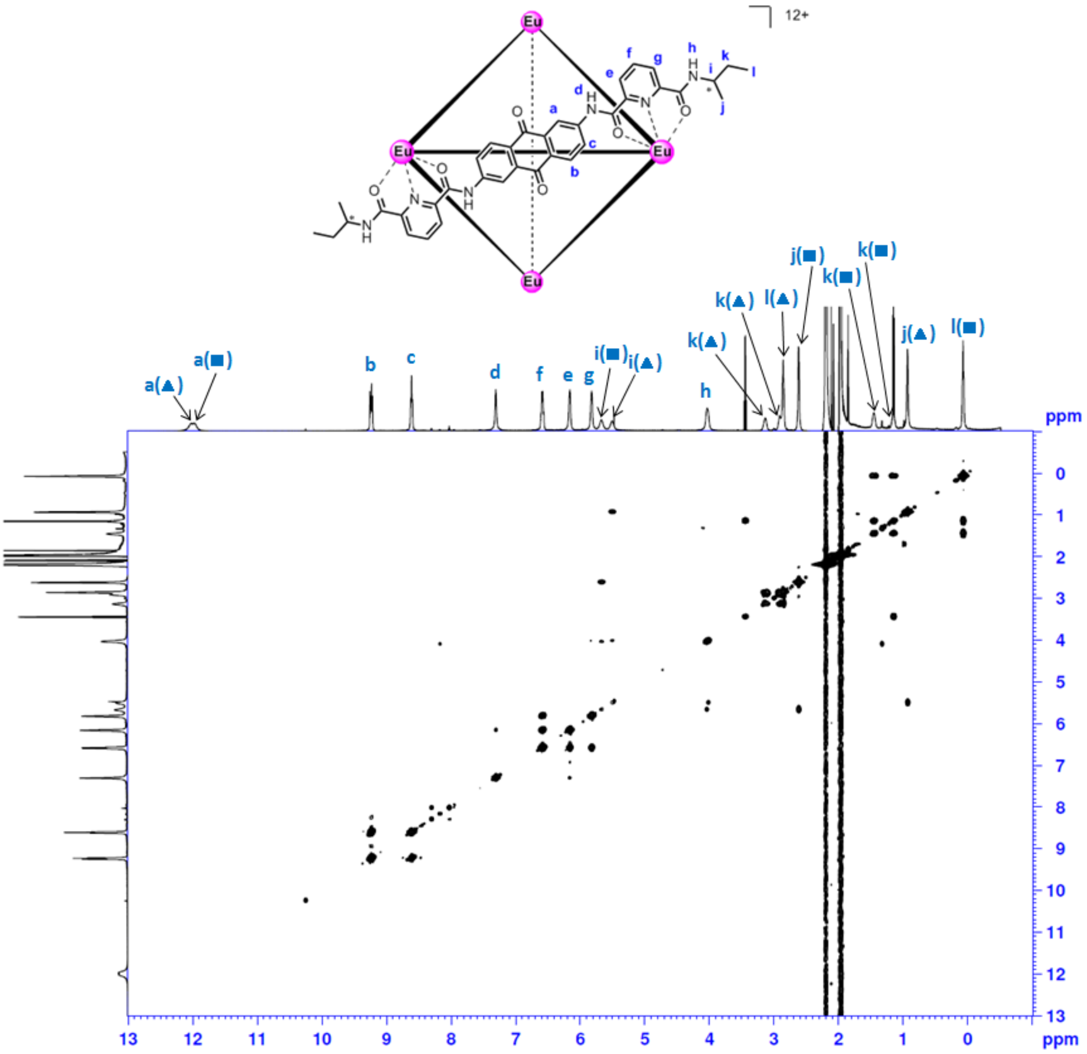

Supplementary Figure 15| ^{1}H–^{1}H **COSY NMR (400 MHz,** CD_3CN**, 296 K) spectrum of** $[Eu_4(L2^{SS})_6](OTf)_{12}$**.** ■ and ▲ represent for the major and minor species, respectively.

a

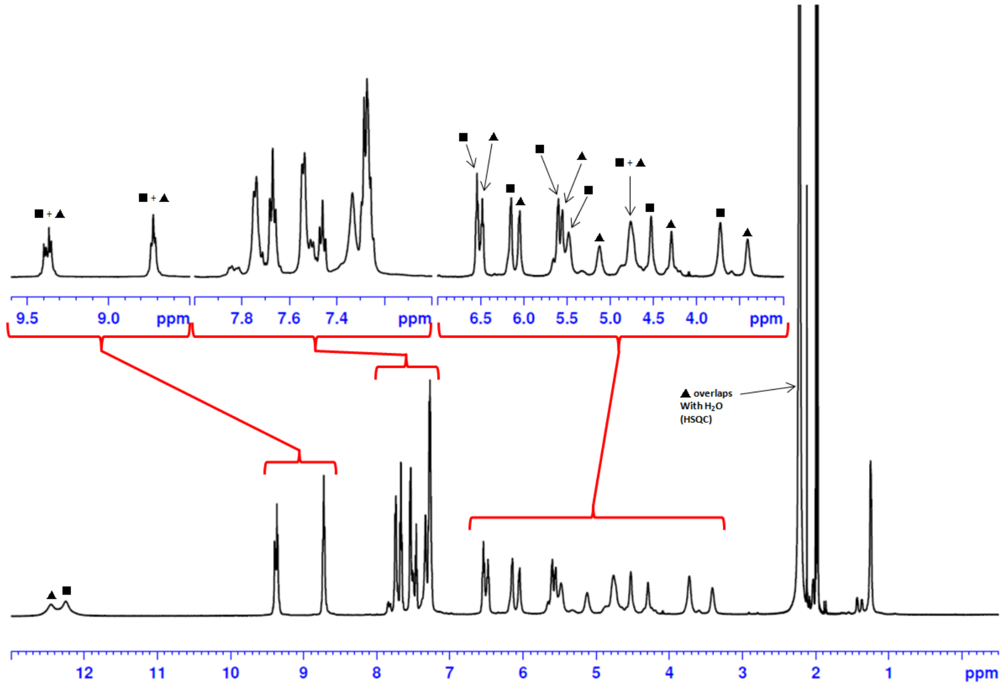

b

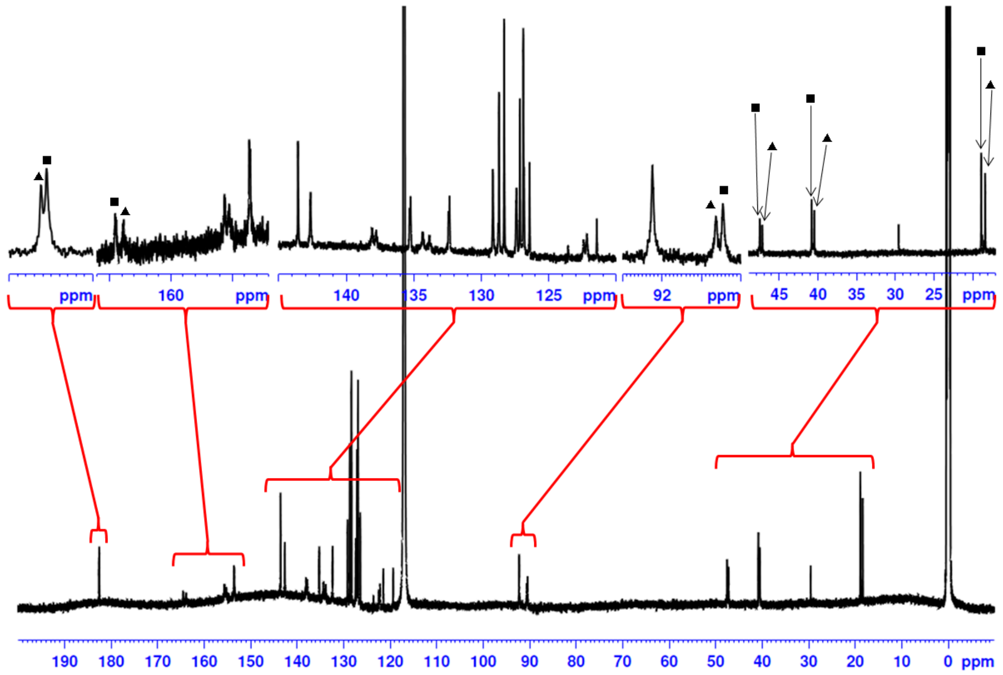

Supplementary Figure 16| ^{1}H NMR and ^{13}C NMR spectra of [$Eu_4(L3^{RR})_6$]$(OTf)_{12}$. (**a**) ^{1}H NMR (400 MHz, CD_3CN, 298 K). The insets are the expanded regions as indicated. ■ and ▲ represent for the major and minor species, respectively. (**b**) ^{13}C NMR (100.6 MHz, CD_3CN, 298 K)**.** ■ and ▲ represent for the major and minor species, respectively.

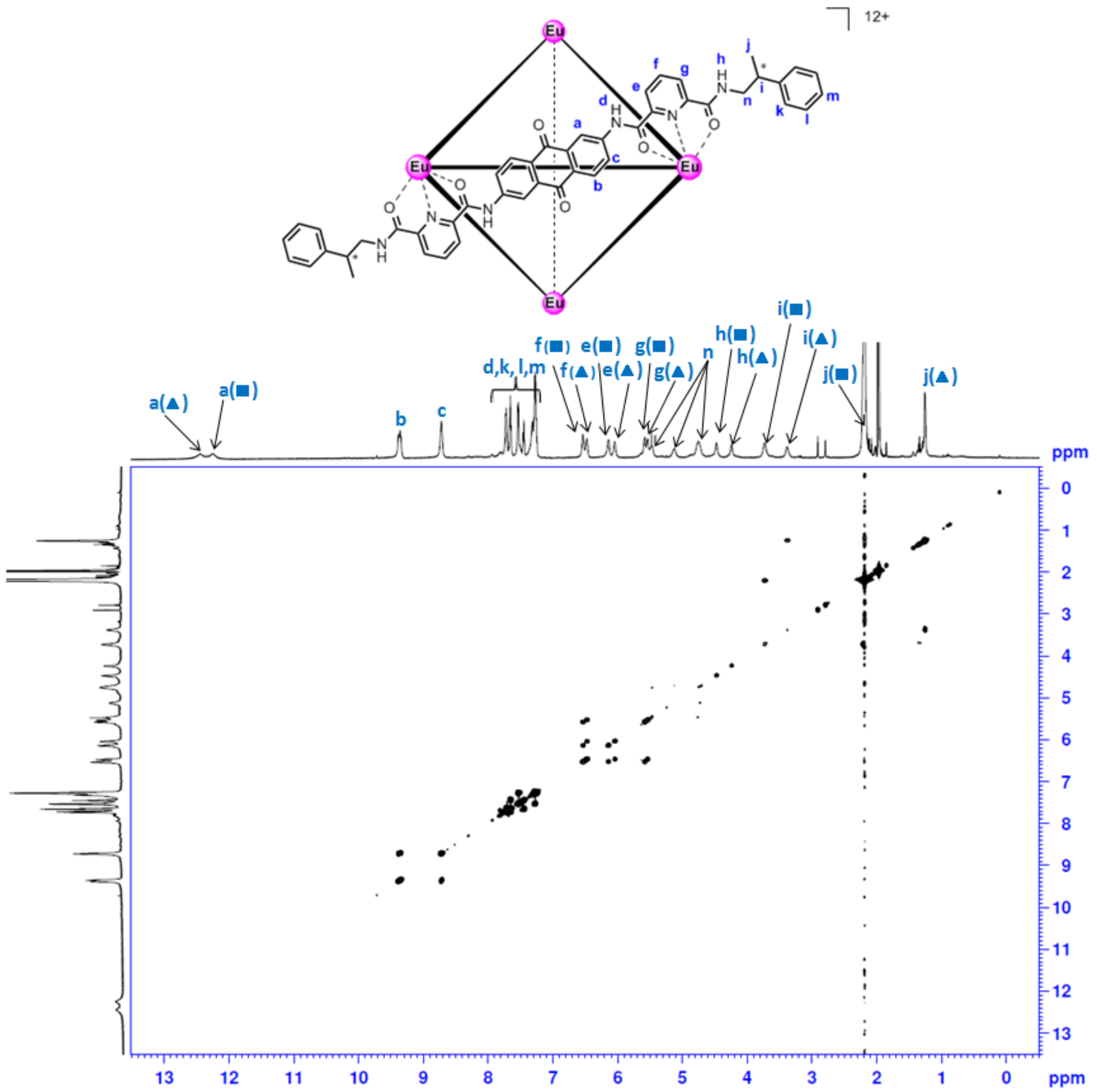

Supplementary Figure 17| ^{1}H–^{1}H COSY NMR (400 MHz, CD_3CN, 296 K) spectrum of [$Eu_4(L3^{SS})_6$]$(OTf)_{12}$. ■ and ▲ represent for the major and minor species,

respectively.

a

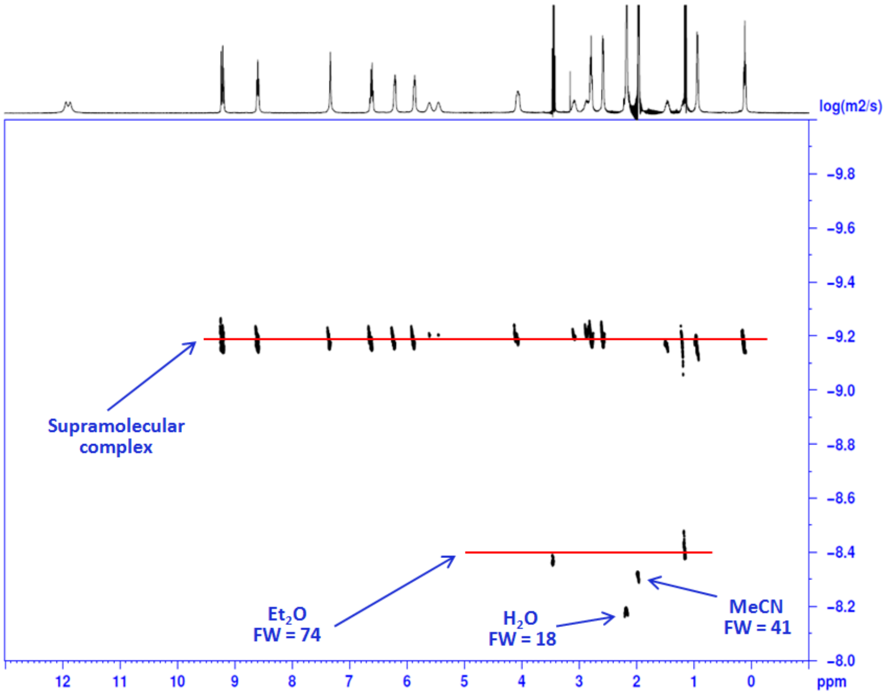

b

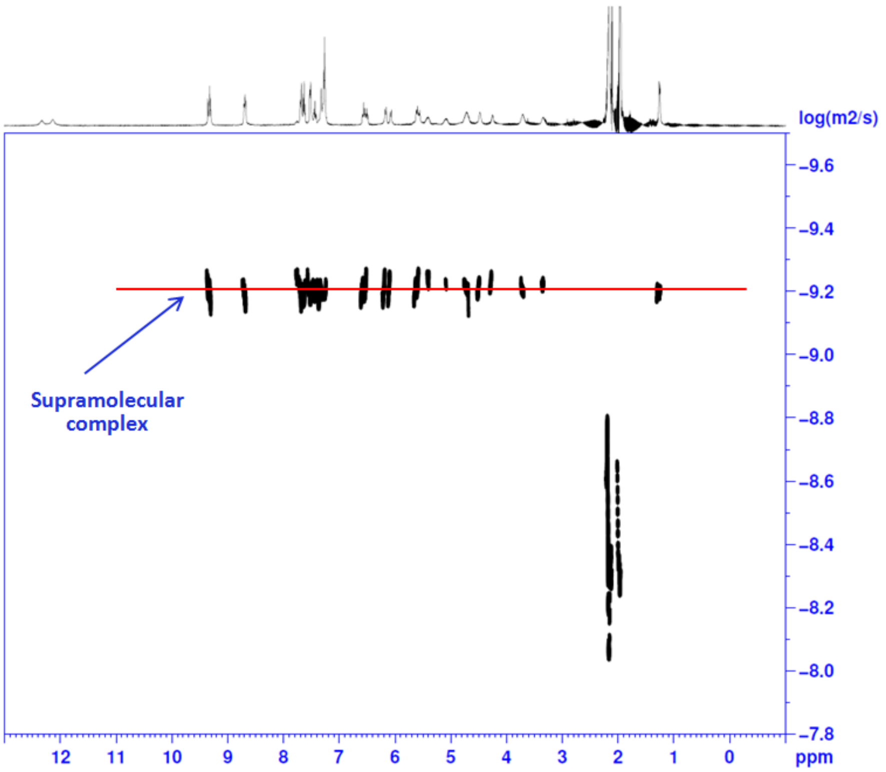

Supplementary Figure 18| DOSY spectra of tetrahedral cages of L2 and L3. (**a**) $[Eu_4(\mathbf{L2}^{SS})_6](OTf)_{12}$ in CD_3CN at 298K. (**b**) $[Eu_4(\mathbf{L3}^{SS})_6](OTf)_{12}$ in CD_3CN at 298K. Majorly one diffusion coefficient can be found in each case.

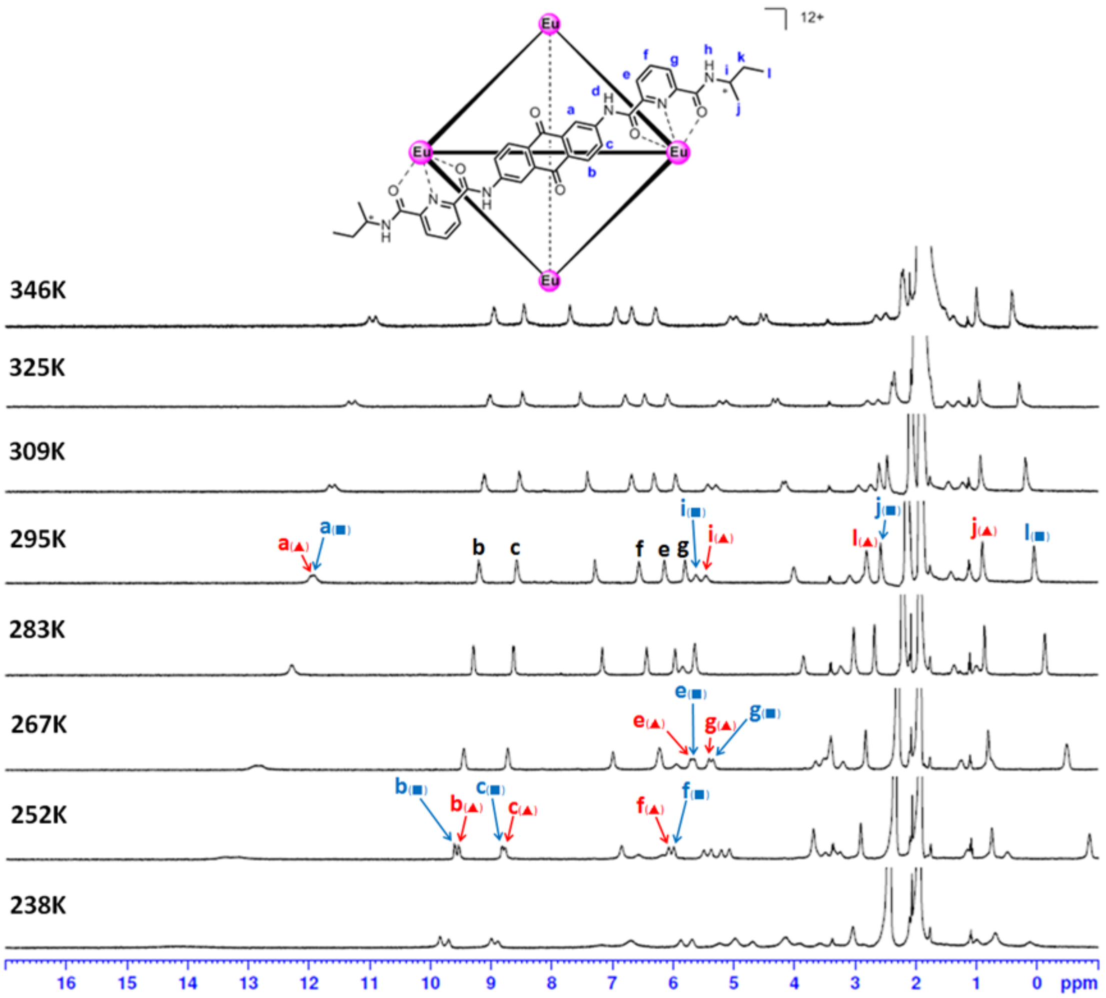

Supplementary Figure 19| Temperature profile (varied from 238 K to 346 K) of ^{1}H NMR spectra of $[Eu_4(L2^{SS})_6](OTf)_{12}$ in CD_3CN. A stack of NMR spectra showing downfield and upfield shifts of resonances. ■ and ▲ represent for the major and minor species, respectively. Labels a–c and e–g are aromatic protons. Labels i, j and l are aliphatic protons.

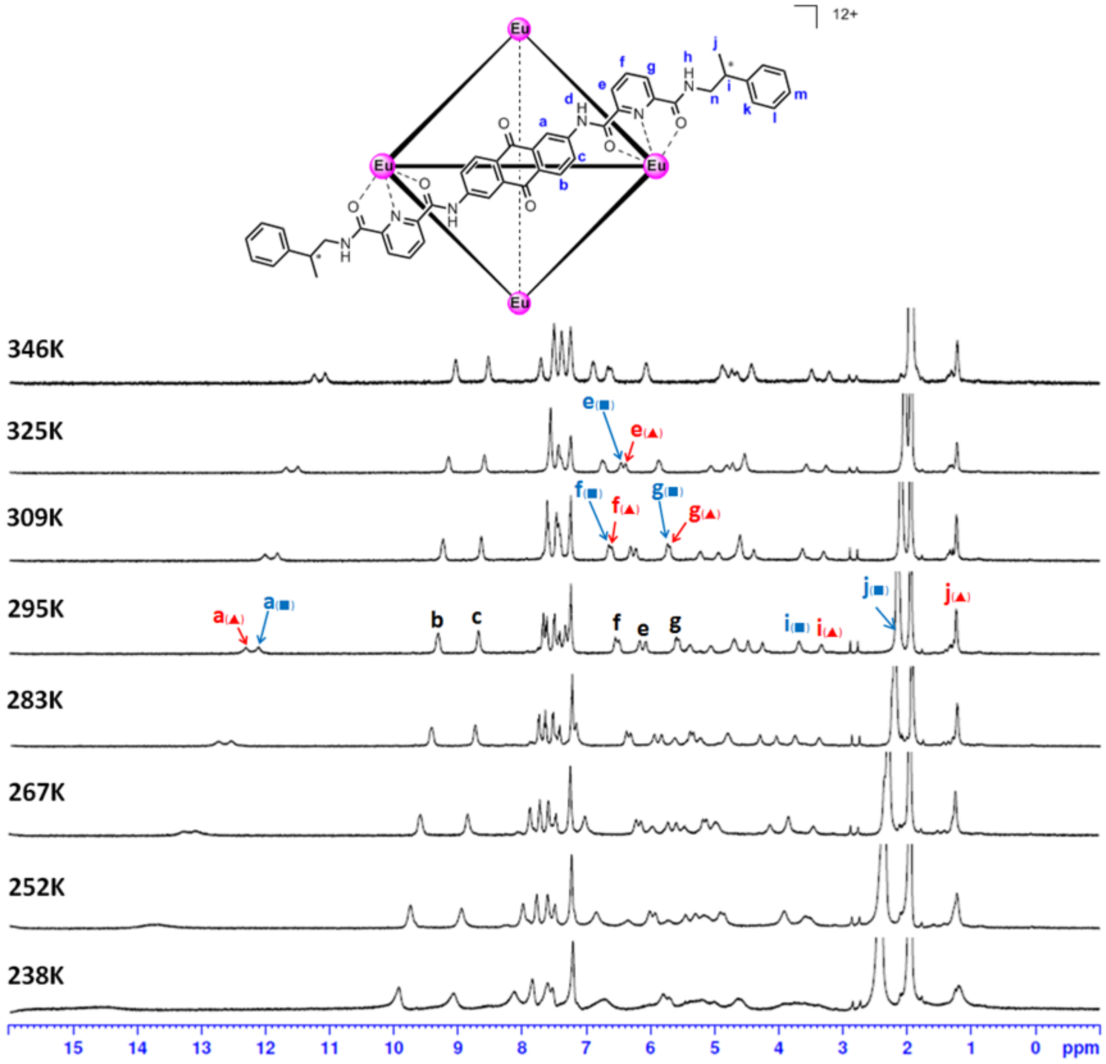

Supplementary Figure 20| Temperature profile (varied from 238 K to 346 K) of ^{1}H NMR spectra of $[Eu_4(L3^{SS})_6](OTf)_{12}$ in CD_3CN. A stack of NMR spectra showing downfield and upfield shifts of resonances. ■ and ▲ represent for the major and minor species, respectively. Labels a–c and e–g are aromatic protons. Labels i and j are aliphatic protons.

a

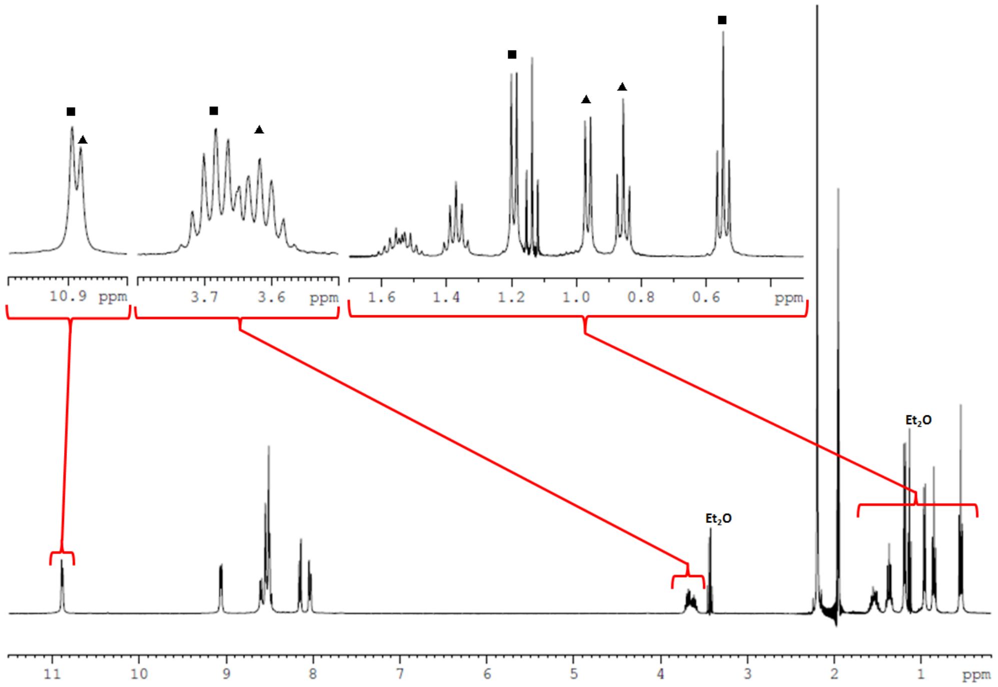

b

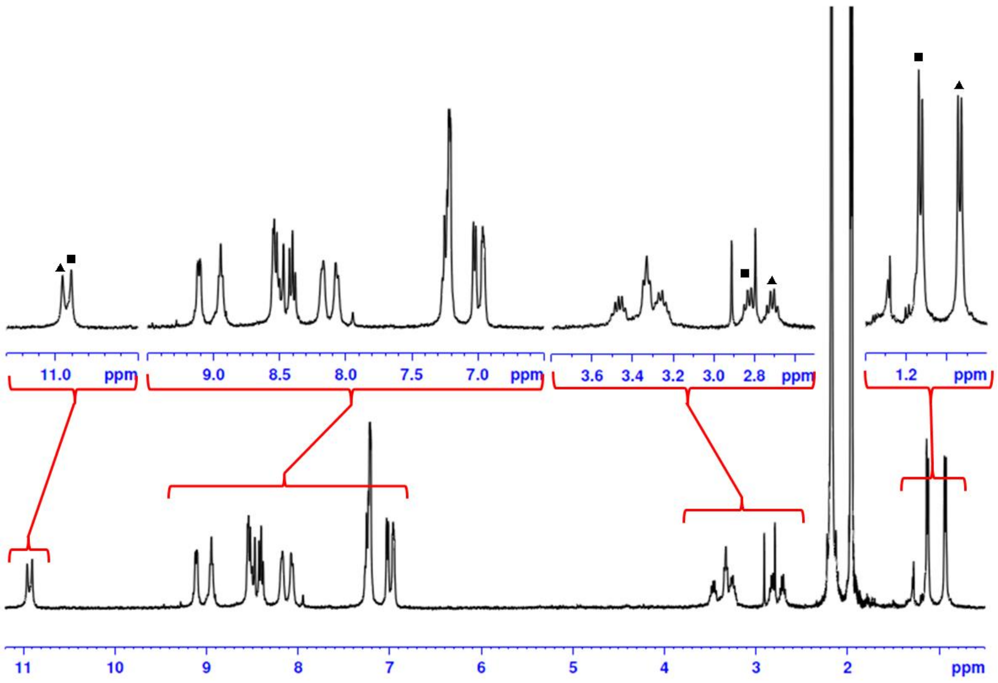

Supplementary Figure 21| ^{1}H NMR spectra of yttrium tetrahedral cages of L2 and L3.

(**a**) $[Y_4(\mathbf{L2}^{\mathbf{RR}})_6](OTf)_{12}$. The insets are the expanded regions as indicated. ■ and ▲

represent for the major and minor species, respectively. (**b**) $[Y_4(\mathbf{L3}^{RR})_6](OTf)_{12}$. The insets are the expanded regions as indicated. ■ and ▲ represent for the major and minor species, respectively.

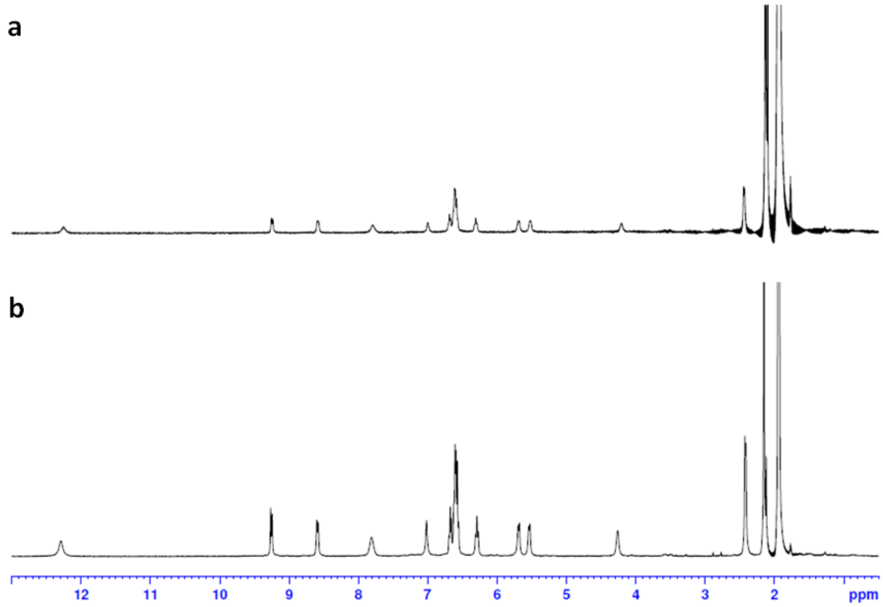

Supplementary Figure 22| ^{1}H NMR spectra of $[Eu_4(L1^{SS})_6](OTf)_{12}$ in d-MeCN in two different concentrations. (a) is a ten-fold dilution of the (b).

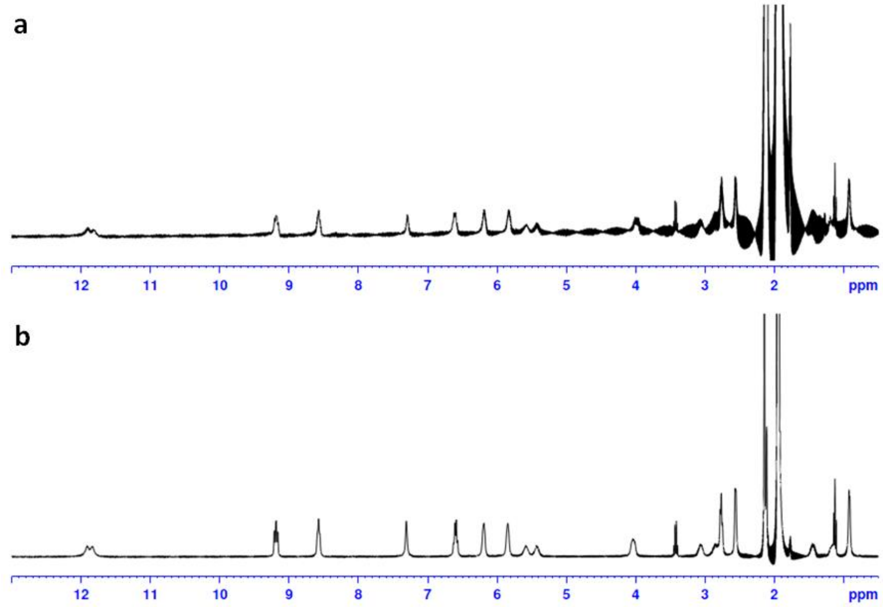

Supplementary Figure 23| ^{1}**H NMR spectra of** $[Eu_4(L2^{SS})_6](OTf)_{12}$ **in d-MeCN in two different concentrations.** (a) is a ten-fold dilution of the (b)

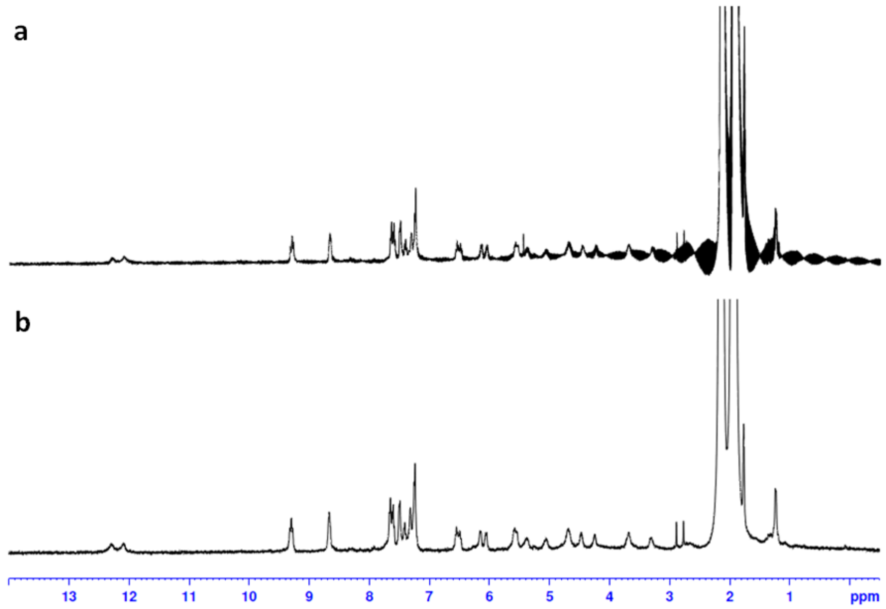

Supplementary Figure 24| ^{1}**H NMR spectra of** $[Eu_4(L3^{RR})_6](OTf)_{12}$ **in d-MeCN in two different concentrations.** (a) is a ten-fold dilution of the (b).

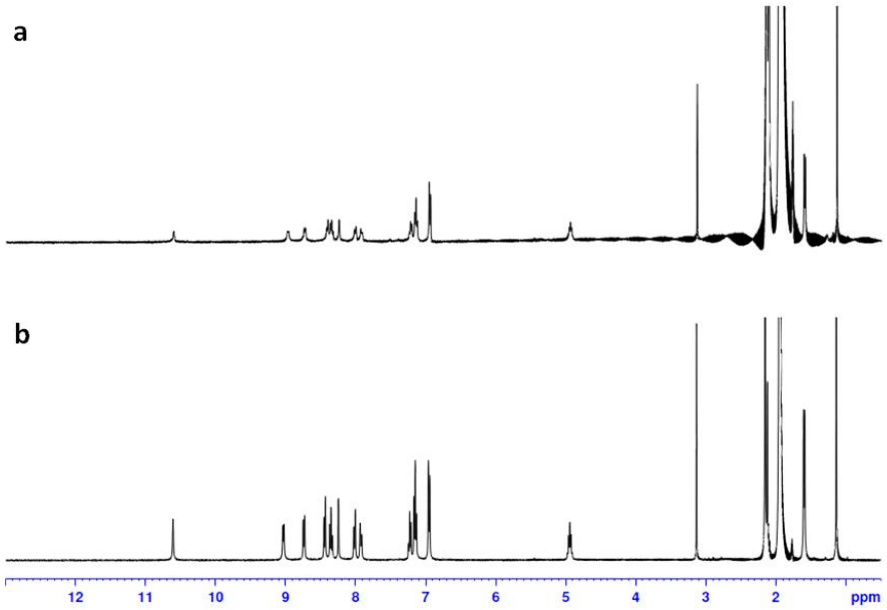

Supplementary Figure 25| ^{1}**H NMR spectra of** $[Y_4(L1^{SS})_6](OTf)_{12}$ **in d-MeCN in two different concentrations.** (a) is a ten-fold dilution of the (b).

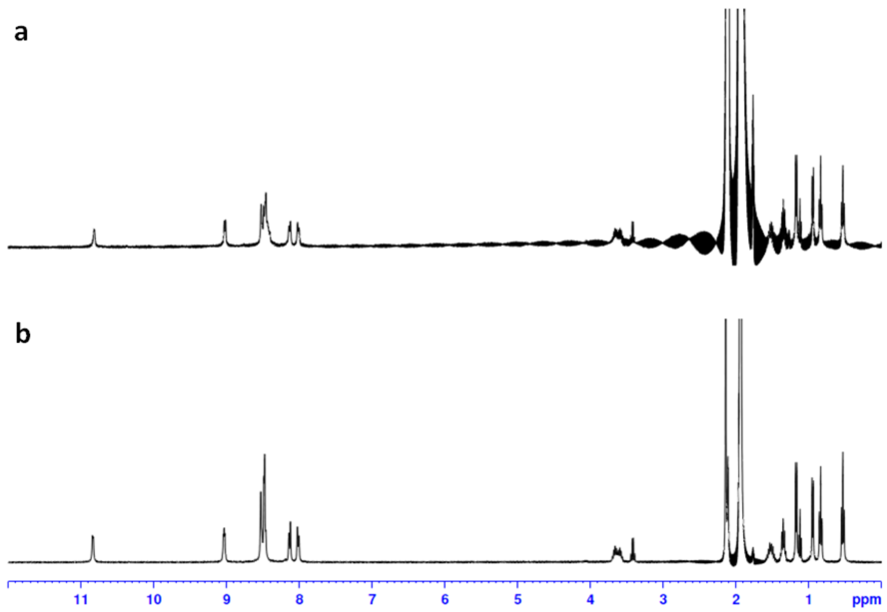

Supplementary Figure 26| ^{1}**H NMR spectra of** $[Y_4(L2^{SS})_6](OTf)_{12}$ **in d-MeCN in two different concentrations.** (a) is a ten-fold dilution of the (b).

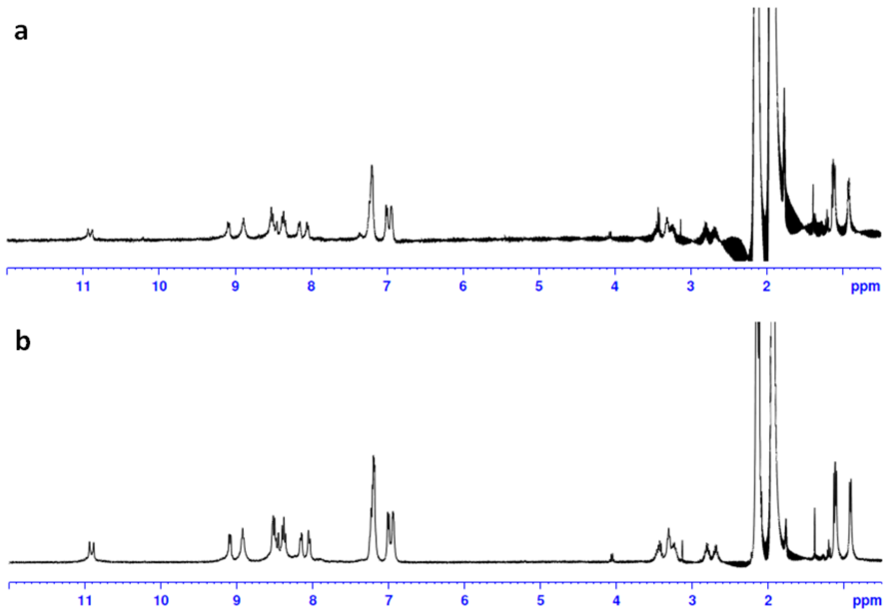

Supplementary Figure 27| ^{1}**H NMR spectra of** $[Y_4(L3^{SS})_6](OTf)_{12}$ **in d-MeCN in two different concentrations.** (a) is a ten-fold dilution of the (b).

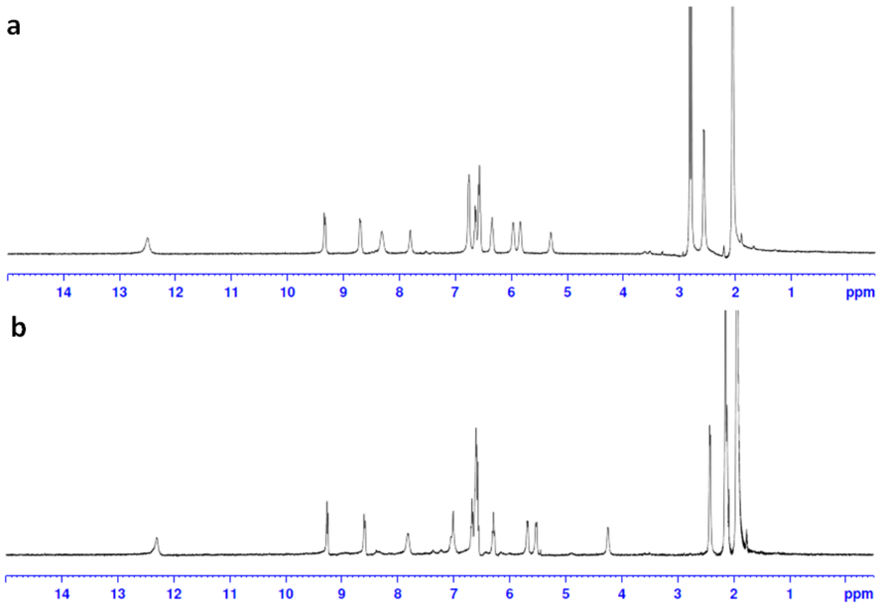

Supplementary Figure 28| ^{1}H NMR spectra of $[Eu_4(L1^{SS})_6](OTf)_{12}$ in two different solvents. (a) is d-acetone. (b) is d-MeCN. This complex is not soluble in d-$CHCl_3$ and d-CH_2Cl_2. And this complex is not stable in d-MeOH and d-DMSO, ligand was recovered.

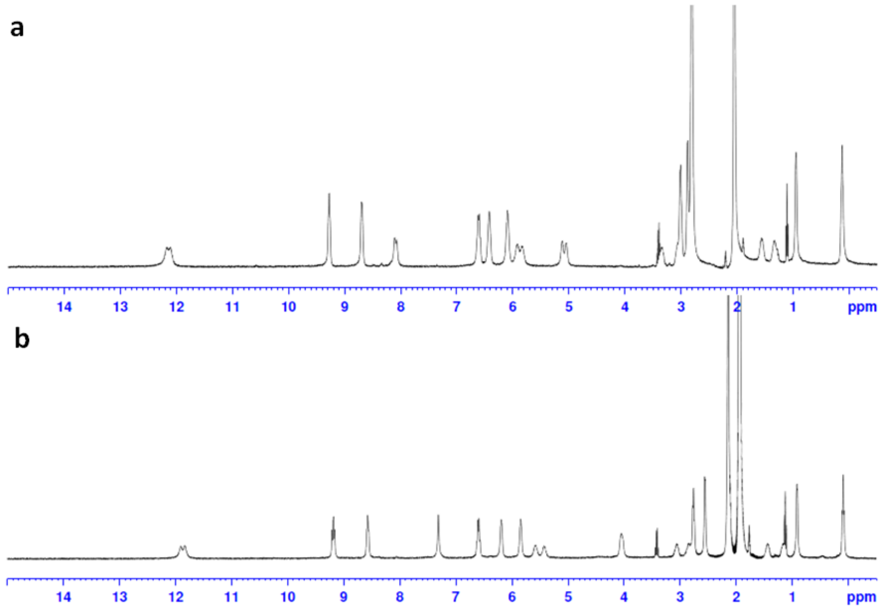

Supplementary Figure 29| ^{1}H NMR spectra of $[Eu_4(L2^{SS})_6](OTf)_{12}$ in two different

solvents. (a) is d-acetone. (b) is d-MeCN. This complex is not soluble in d-$CHCl_3$ and d-CH_2Cl_2. And this complex is not stable in d-MeOH and d-DMSO, ligand was recovered.

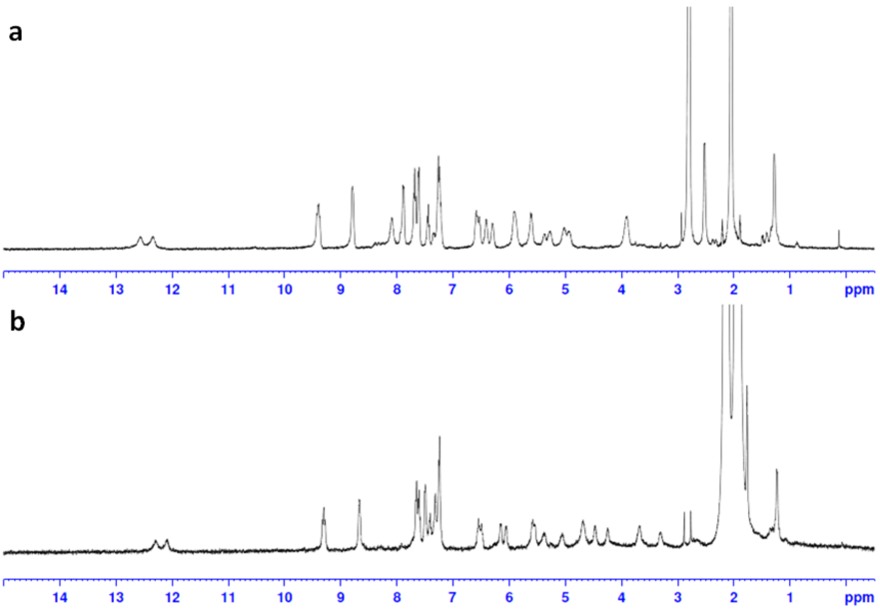

Supplementary Figure 30| ^{1}H NMR spectra of $[Eu_4(L3^{SS})_6](OTf)_{12}$ in two different solvents. (a) is d-acetone. (b) is d-MeCN. This complex is not soluble in d-$CHCl_3$ and d-CH_2Cl_2. And this complex is not stable in d-MeOH and d-DMSO, ligand was recovered.

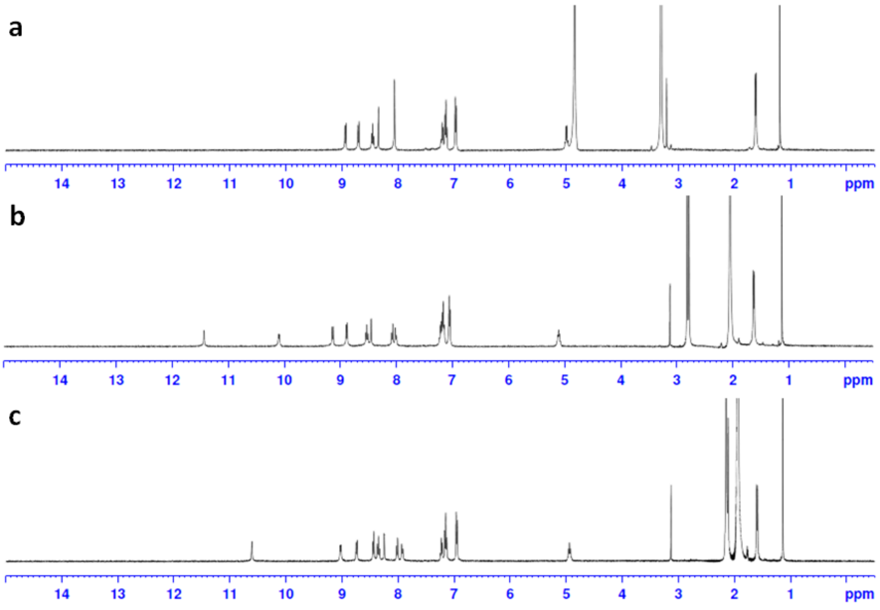

Supplementary Figure 31| ^{1}H NMR spectra of $[Y_4(L1^{SS})_6](OTf)_{12}$ in three different

solvents. (a) is d-MeOH, disappearance of two NHs chemical shifts due to solvent exchange (b) is d-acetone and (c) is d-MeCN. This complex is not soluble in d-$CHCl_3$ and d-CH_2Cl_2. And this complex is not stable in d-DMSO, ligand was recovered.

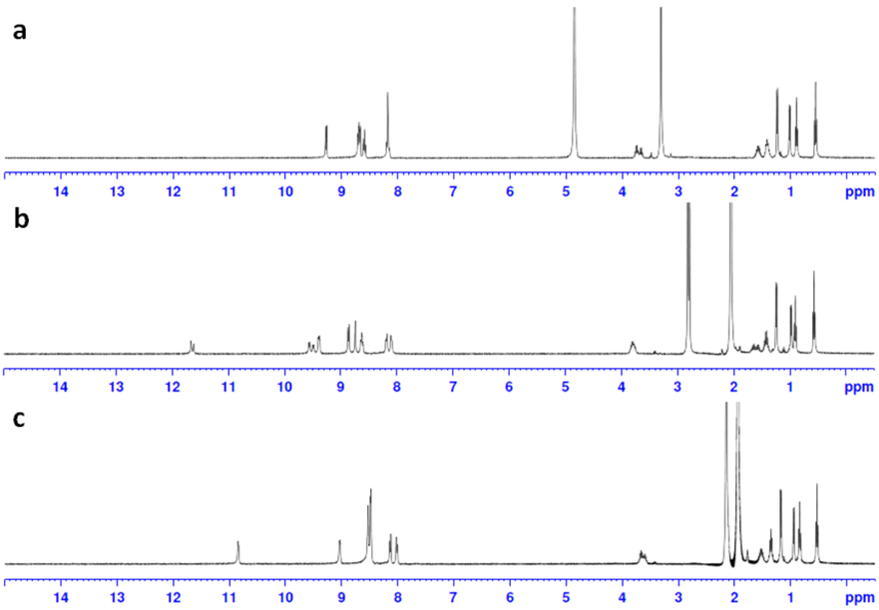

Supplementary Figure 32| ^{1}**H NMR spectra of** $[Y_4(L2^{SS})_6](OTf)_{12}$ **in three different solvents.** (a) is d-MeOH, disappearance of two NHs chemical shifts due to solvent exchange (b) is d-acetone and (c) is d-MeCN. This complex is not soluble in d-$CHCl_3$ and d-CH_2Cl_2. And this complex is not stable in d-DMSO, ligand was recovered.

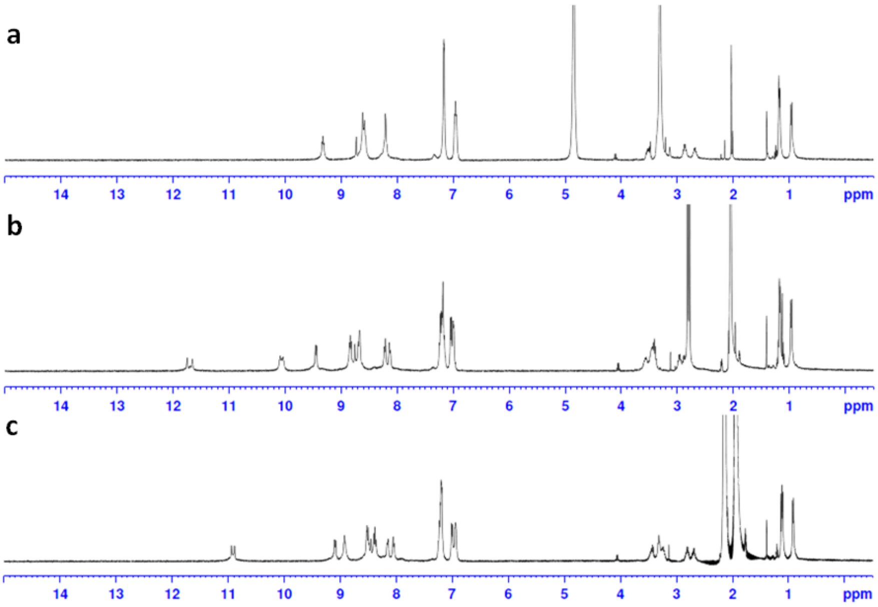

Supplementary Figure 33| ^{1}**H NMR spectra of** $[Y_4(L3^{SS})_6](OTf)_{12}$ **in three different**

solvents. (a) is d-MeOH, disappearance of two NHs chemical shifts due to solvent exchange (b) is d-acetone and (c) is d-MeCN. This complex is not soluble in d-$CHCl_3$ and d-CH_2Cl_2. And this complex is not stable in d-DMSO, ligand was recovered.

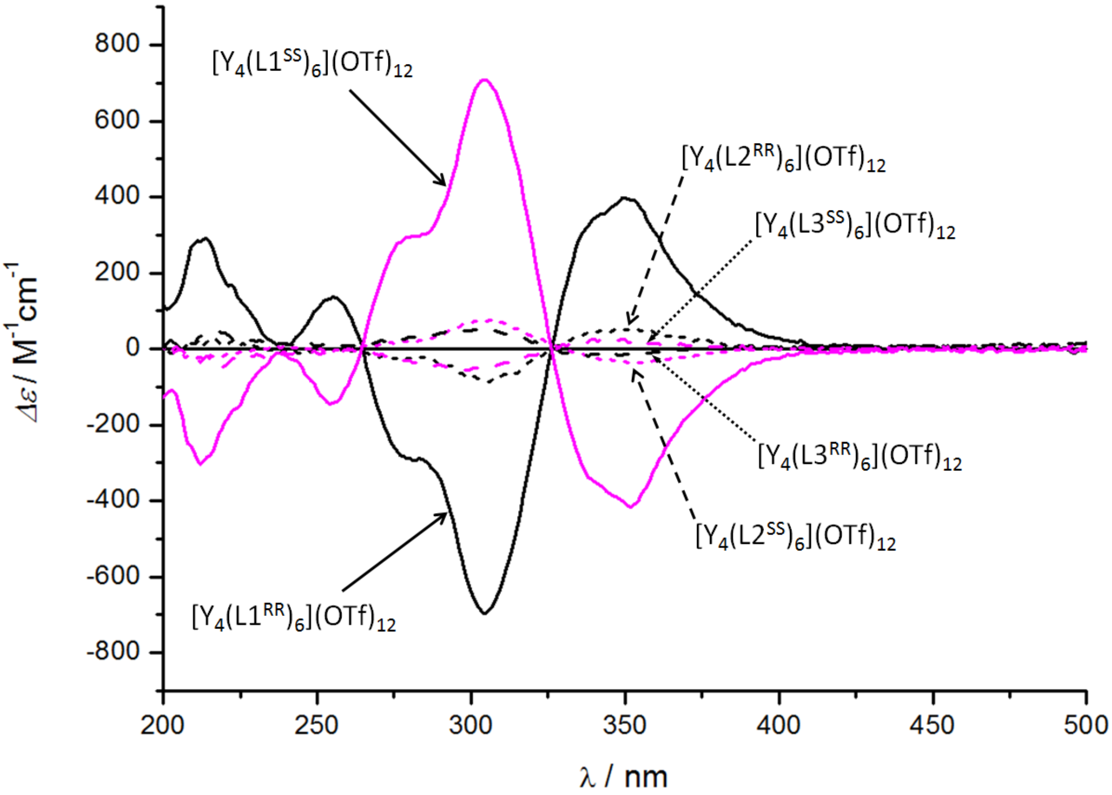

Supplementary Figure 34| CD spectra of yttrium tetrahedral cages from L1–L3. $[Y_4(\mathbf{L1})_6](OTf)_{12}$, $[Y_4(\mathbf{L2})_6](OTf)_{12}$, $[Y_4(\mathbf{L3})_6](OTf)_{12}$ in MeCN. Signals attenuation: $[Y_4(\mathbf{L2})_6](OTf)_{12}$ [89(3)% (353 nm), 89(1)% (305 nm), 92(1)% (279 nm), 99(2)% (255 nm) and 92(3)% (212 nm)]; $[Y_4(\mathbf{L3})_6](OTf)_{12}$ [96(1)% (353 nm), 93(1)% (305 nm), 90(1)% (279 nm), 94(4)% (255 nm) and 88(2)% (212 nm)]. (Note: Cotton effect of $[Y_4(\mathbf{L2})_6](OTf)_{12}$ is slightly stronger than $[Y_4(\mathbf{L3})_6](OTf)_{12}$ whereas the Cotton effect of $[Eu_4(\mathbf{L3})_6](OTf)_{12}$ is stronger than $[Eu_4(\mathbf{L2})_6](OTf)_{12}$. This observation may probably be due to a slightly smaller ionic radius of Y^{3+} compared to Eu^{3+}, hence altering their diastereoselective preferences to ΛΛΛΛ– or ΔΔΔΔ–isomers. Although the orders are inverted, the preferences to achieve either the positive or negative sign of Cotton effect are same for both cages Eu and Y, where in both case they originated from the same chirality of the ligands.)

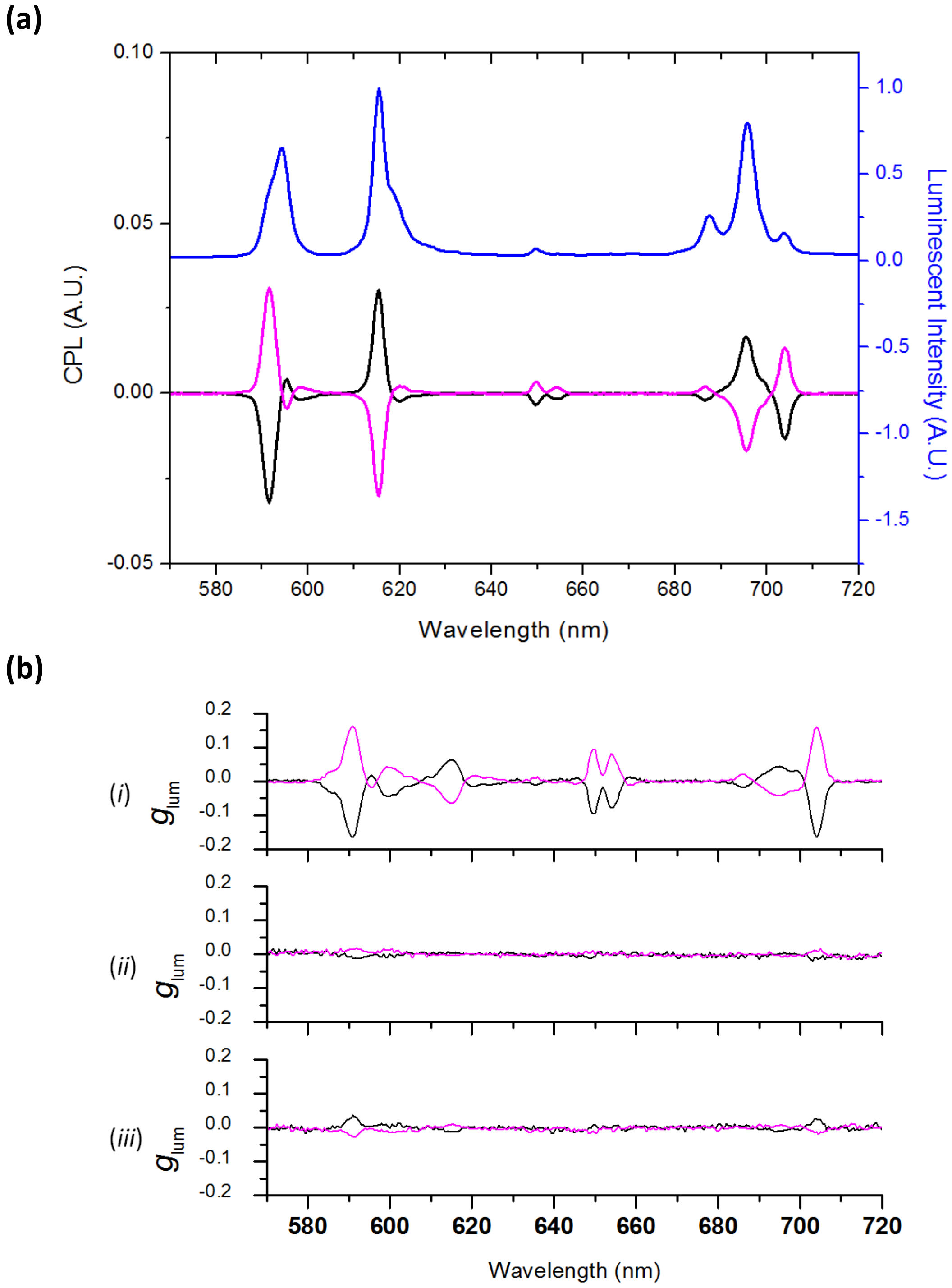

Supplementary Figure 35| CPL of $[Eu_4(L1^{RR})_6](OTf)_{12}$ and a stack of plots of g_{lum}

($2\Delta I/I$) values of tetrahedral cages from L1–L3. (a) The luminescence emission spectrum of the [Eu_4(**L1**^{RR})$_6$](OTf)$_{12}$ (blue) and the circular polarized emission spectra of [Eu_4(**L1**^{RR})$_6$](OTf)$_{12}$ (black) and [Eu_4(**L1**^{SS})$_6$](OTf)$_{12}$ (magenta) in MeCN. **(b)** g_{lum} values of (*i*) [Eu_4(**L1**)$_6$](OTf)$_{12}$ [**L** = **L1**^{RR} (black) or **L1**^{SS} (magenta)], (*ii*) [Eu_4(**L2**)$_6$](OTf)$_{12}$ [**L** = **L2**^{RR} (black) or **L2**^{SS} (magenta)], (*iii*) [Eu_4(**L3**)$_6$](OTf)$_{12}$ [**L** = **L3**^{RR} (black) or **L3**^{SS} (magenta)].

Supplementary Table 2| Summary of CPL results for the four tetrahedral cages.

		g_{lum}*			
Electronic transition	Wavelength (nm)	[Eu_4(**L1**^{RR})$_6$](OTf)$_{12}$	[Eu_4(**L1**^{SS})$_6$](OTf)$_{12}$	[Eu_4(**L2**^{RR})$_6$](OTf)$_{12}$	[Eu_4(**L2**^{SS})$_6$](OTf)$_{12}$
$^5D_0 \rightarrow {}^7F_1$	591.0	–0.16(1)	0.16(1)	0.04(2)	–0.03(2)
	599.5	–0.05(1)	0.04(1)		
$^5D_0 \rightarrow {}^7F_2$	615.0	0.06(1)	–0.07(1)		
$^5D_0 \rightarrow {}^7F_3$	649.5	–0.10(2)	0.10(2)		
	654.0	–0.07(3)	0.08(2)		
$^5D_0 \rightarrow {}^7F_4$	686.0	–0.02(1)	0.02(1)		
	695.0	0.04(1)	–0.04(1)		
	704.0	–0.16(2)	0.16(1)		

*The numbers in the parenthesis are standard deviations

a

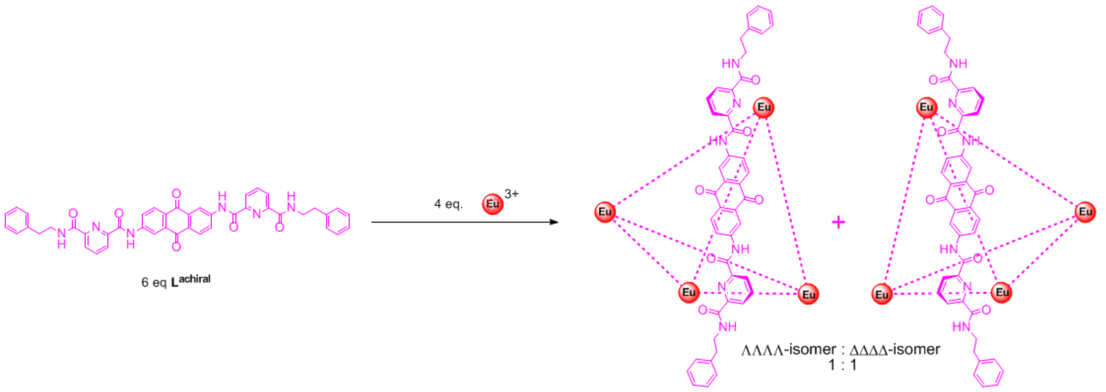

b

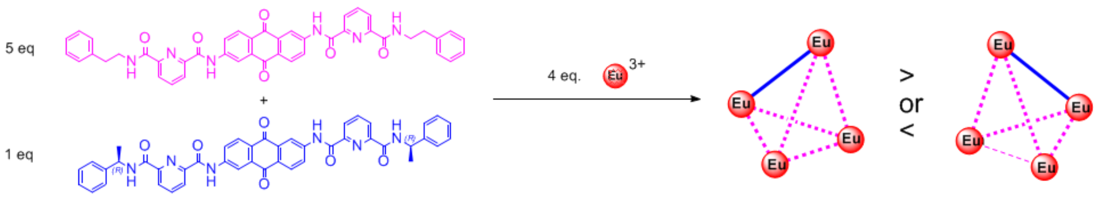

c

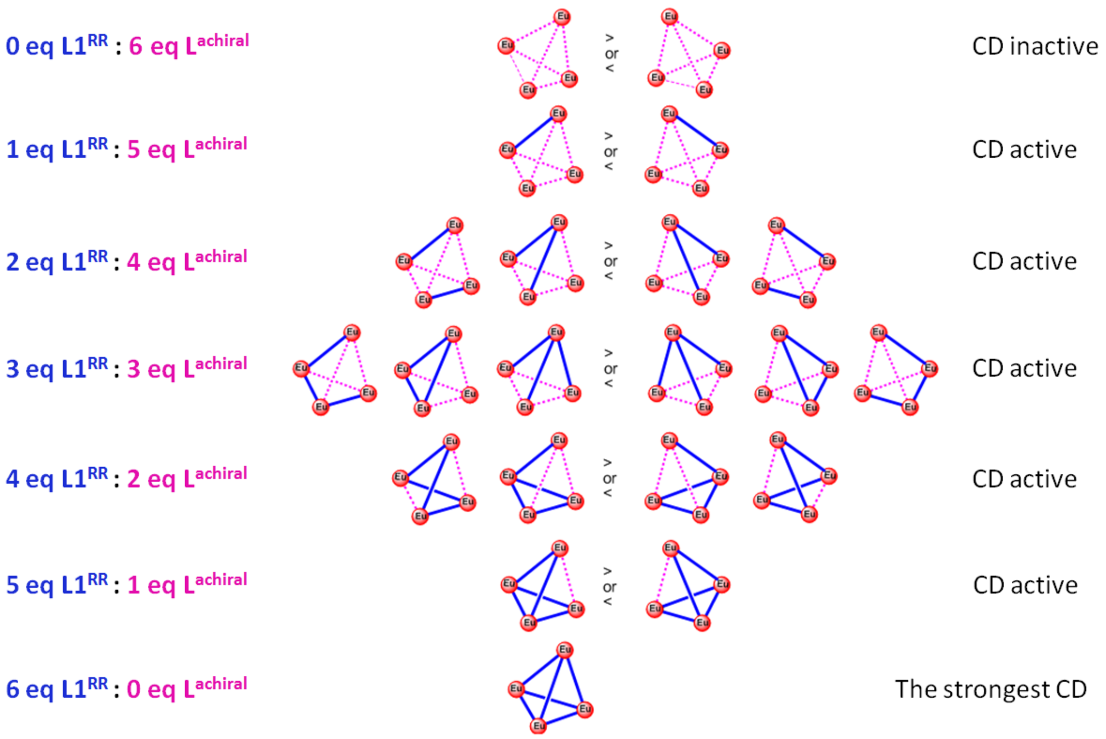

d

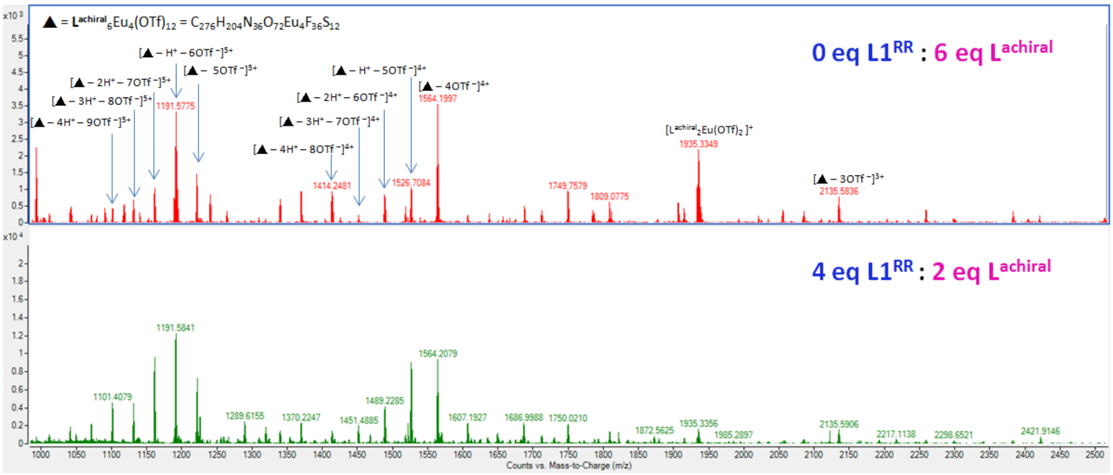

Supplementary Figure 36| Chiral amplification experiment. (**a**) With achiral ligand, L^{achiral}, a racemic mixture of tetrahedral cage, ΛΛΛΛ-[(**L^{achiral}**)$_6$Eu$_4$(OTf)$_{12}$] and ΔΔΔΔ-[(**L^{achiral}**)$_6$Eu$_4$(OTf)$_{12}$], are formed in 1 to 1 ratio. (**b**) By substituting one **L^{achiral}** with one **L1^{RR}**, either ΛΛΛΛ-[(**L1^{RR}**)$_1$(**L^{achiral}**)$_5$Eu$_4$(OTf)$_{12}$] or ΔΔΔΔ-[(**L1^{RR}**)$_1$(**L^{achiral}**)$_5$Eu$_4$(OTf)$_{12}$] should be formed in diastereomeric excess (d.e.)

because of chiral inducing ability of **L1^{RR}.** The resulting complex should give CD signal. (**c**) By continuously substituting **L^{achiral}** (6-n) eq. with **L1^{RR}** (n) eq., more complicated heteromeric assemblies [(**L1^{RR}**)$_n$(**L^{achiral}**)$_{6-n}$Eu$_4$(OTf)$_{12}$] (n = 0–6) should be formed and hence resulted in different extent of CD intensity. According to different combinations of **L1^{RR}** and **L^{achiral}**, possible isomers (homoconfiguratial isomers are shown only) of tetrahedral cages in these series of reactions. The corresponding d.e. values will be lower than d.e. of [(**L1^{RR}**)$_6$(**L^{achiral}**)$_0$Eu$_4$(OTf)$_{12}$] as a result of reduced number of chiral ligand. (**d**) ESI-MS spectra of tetrahedral cage that formed from two different combinations of **L^{achiral}** and **L1^{RR}**, 0 eq of **L1^{RR}** to 6 eq of **L^{achiral}** (top) 4 eq of **L1^{RR}** to 2 eq of **L^{achiral}** (low). This comparison may help us to propose that supramolecular molecules other than tetrahedral cage should not be formed significantly. Noted: Molecular formula of **L1^{RR}** and **L^{achiral}** are the same ($C_{44}H_{34}N_6O_6$).

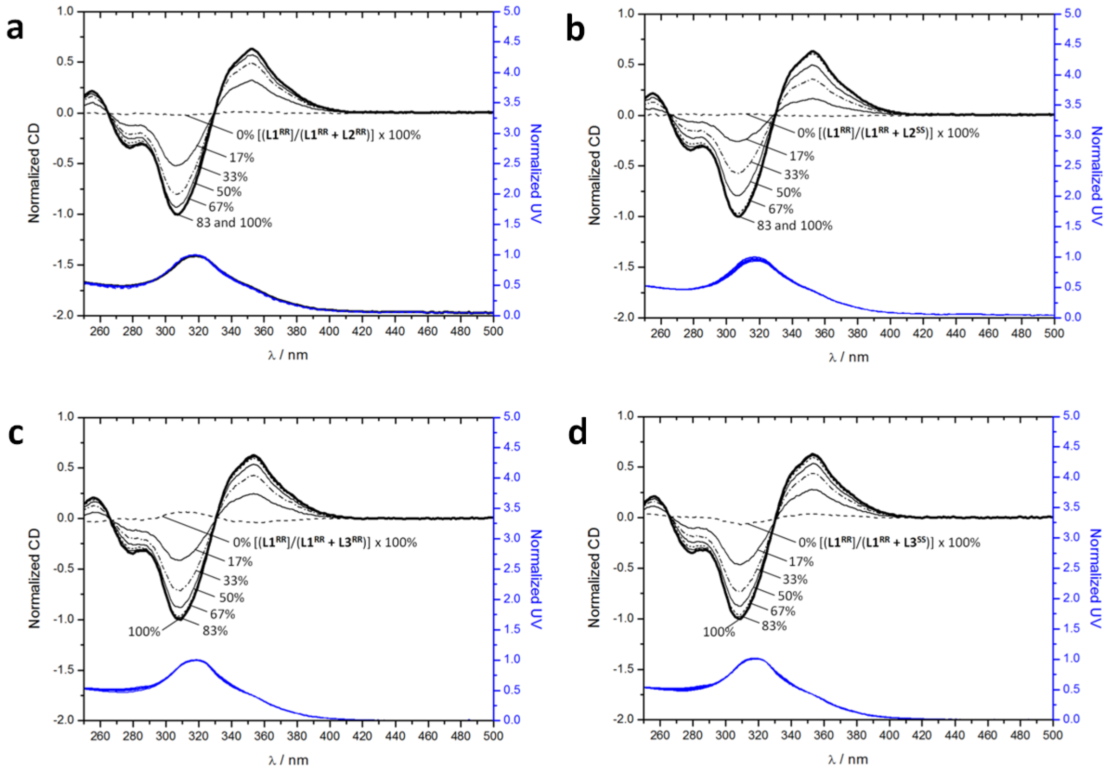

Supplementary Figure 37| Normalized CD and UV-Vis spectra of chiral amplification experiments of tetrahedral cage formation from L2^{RR}, L2^{SS}, L3^{RR} and L3^{SS} with L1^{RR}. (**a**) Normalized CD (top) and normalized UV-Vis absorption spectra (bottom) of supramolecular cage [(**L1**^{RR})$_n$(**L2**^{RR})$_{6-n}$Eu$_4$(OTf)$_{12}$] (n = 0–6) in CH_2Cl_2/MeOH/MeCN (73:3:24, v/v/v) by maintaining 2.05 x 10^{-5} M of [(**L1**^{RR})$_n$(**L2**^{RR})$_{6-n}$Eu$_4$(OTf)$_{12}$] (n = 0–6). (**b**) Same as (**a**) but using **L2**^{SS} instead of **L2**^{RR}. (**c**) Same as (**a**) but using **L3**^{RR} instead of **L2**^{RR}. (**d**) Same as (**a**) but using **L3**^{SS} instead of **L2**^{RR}.

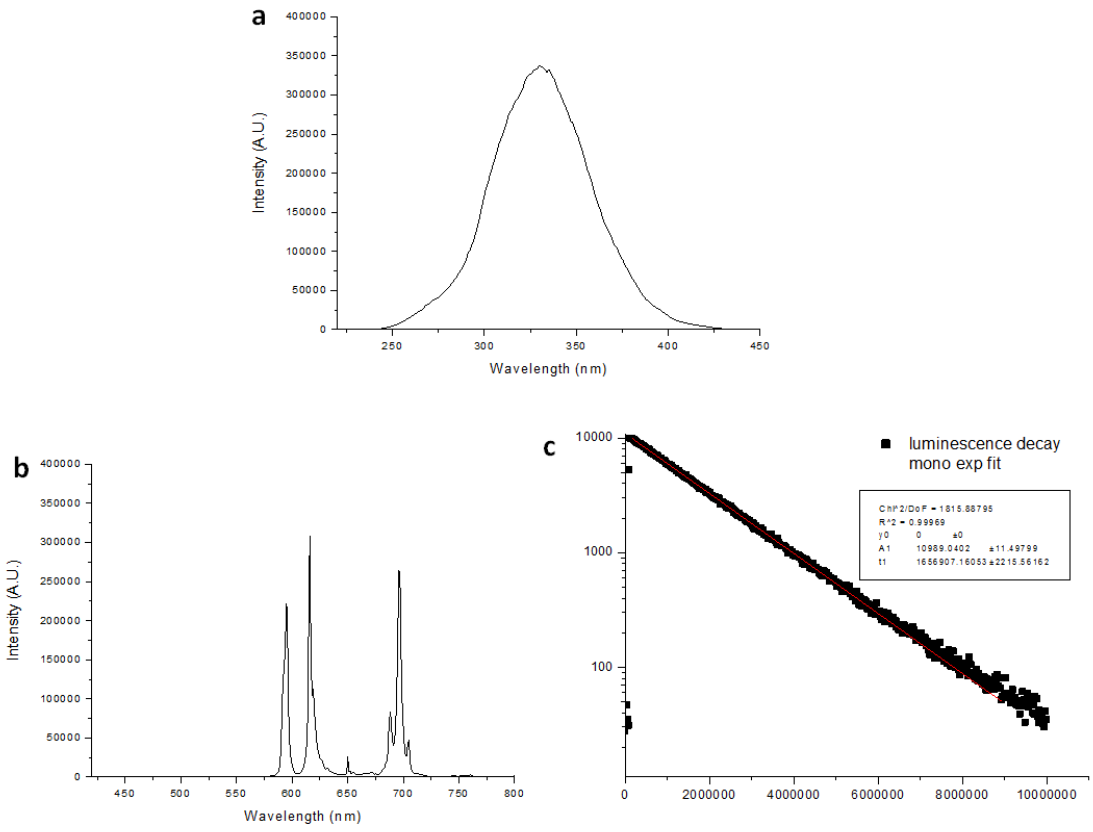

Supplementary Figure 38| Luminescent data of $[Eu_4(L1^{RR})_6](OTf)_{12}$ (1.65 x 10^{-5} M in MeCN). (**a**) Excitation spectrum, λem = 616 nm, slits = 1-0.5, filter 395 nm. (**b**) Emission spectrum, λex = 337 nm, slits = 1-0.5, filter 395 nm. (**c**) Excited state decay curve with mono-exponential fit, λem = 616 nm, slits = 3-2, filter 395 nm.

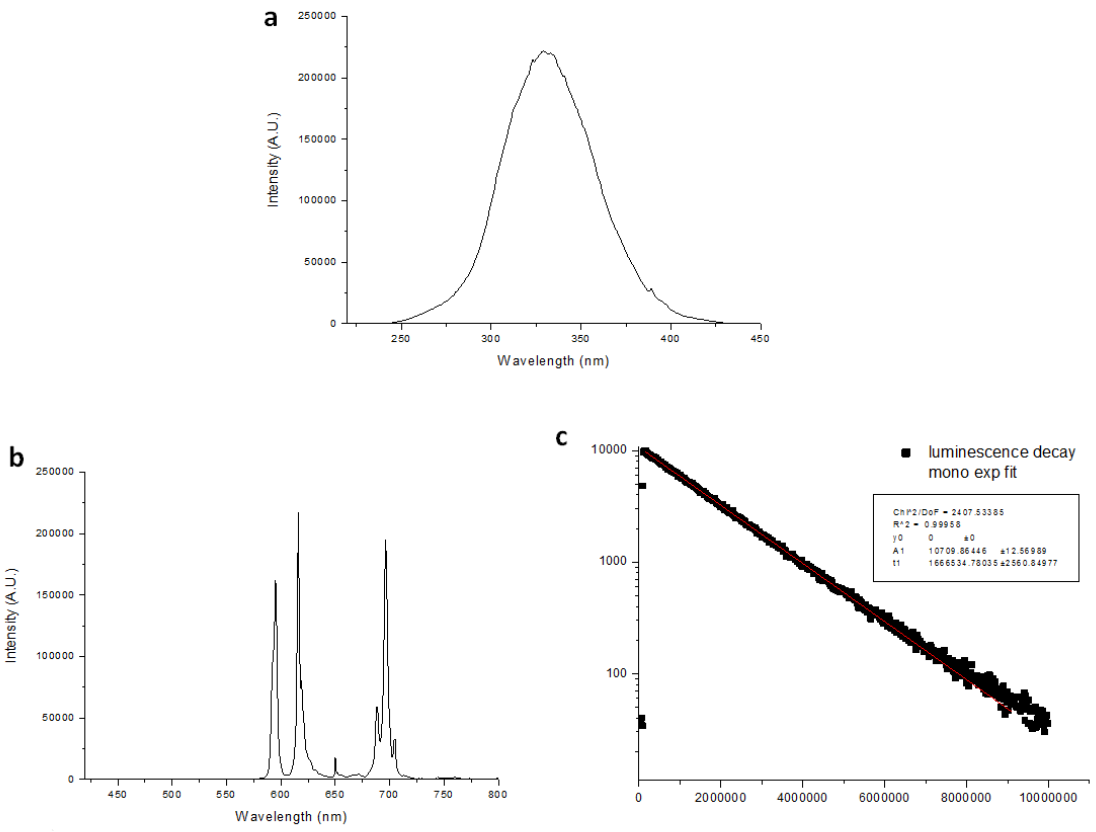

Supplementary Figure 39| Luminescent data of $[Eu_4(L1^{SS})_6](OTf)_{12}$ (1.19 x 10^{-5} M in MeCN). (**a**) Excitation spectrum, λem = 616 nm, slits = 1-0.5, filter 395 nm. (**b**) Emission spectrum, λex = 337 nm, slits = 1-0.5, filter 395 nm. (**c**) Excited state decay curve with mono-exponential fit, λem = 616 nm, slits = 3-2, filter 395 nm.

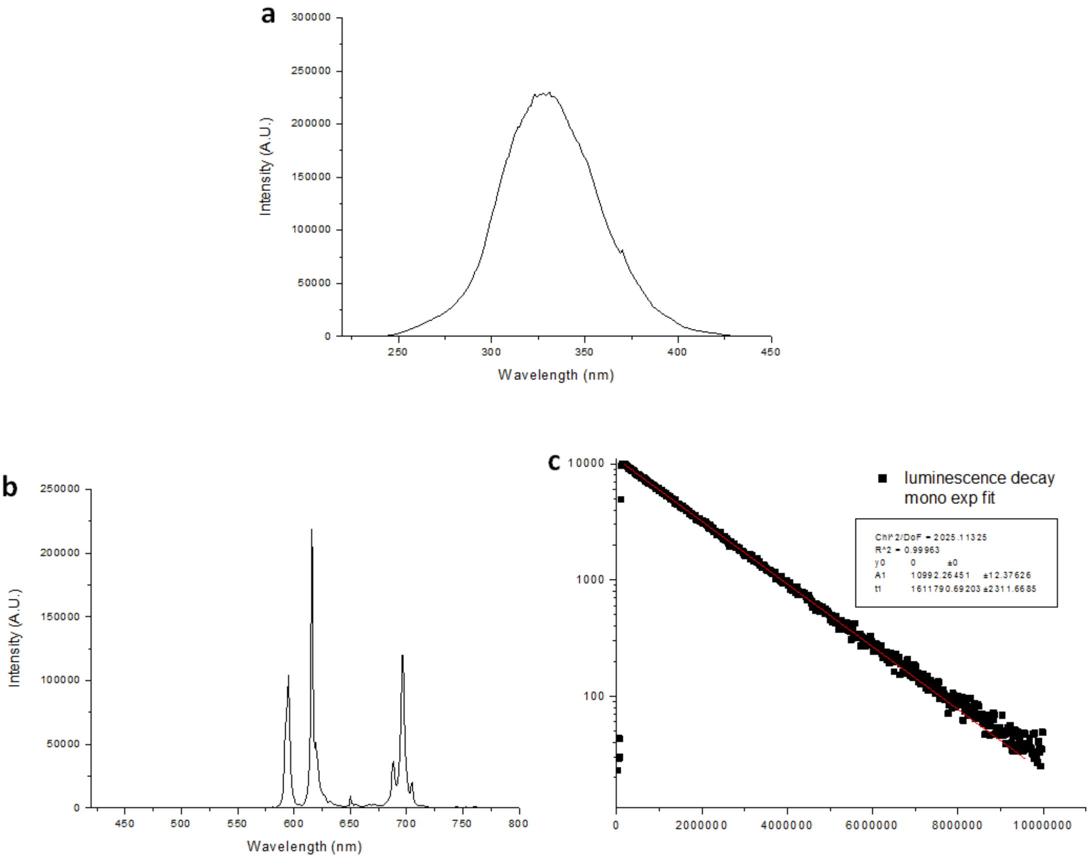

Supplementary Figure 40| Luminescent data of $[Eu_4(L2^{RR})_6](OTf)_{12}$ (1.31 x 10^{-5} M in MeCN). (**a**) Excitation spectrum, λem = 616 nm, slits = 1-0.5, filter 395 nm. (**b**) Emission spectrum, λex = 337 nm, slits = 1-0.5, filter 395 nm. (**c**) Excited state decay curve with mono-exponential fit, λex = 616 nm, slits = 3-2, filter 395 nm.

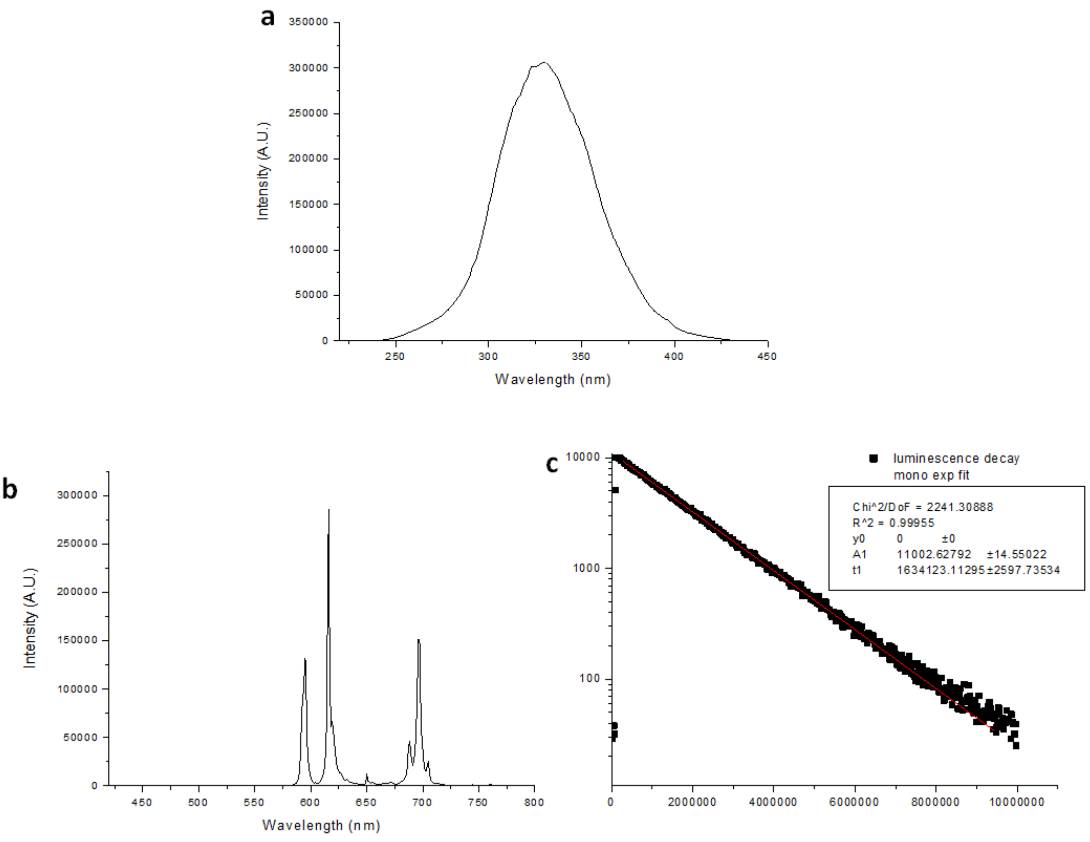

Supplementary Figure 41| Luminescent data of $[Eu_4(L2^{SS})_6](OTf)_{12}$ (1.42 x 10^{-5} M in MeCN). (**a**) Excitation spectrum, λem = 616 nm, slits = 1-0.5, filter 395 nm. (**b**) Emission spectrum, λex = 337 nm, slits = 1-0.5, filter 395 nm. (**c**) Excited state decay curve with mono-exponential fit, λex = 616 nm, slits = 3-2, filter 395 nm.

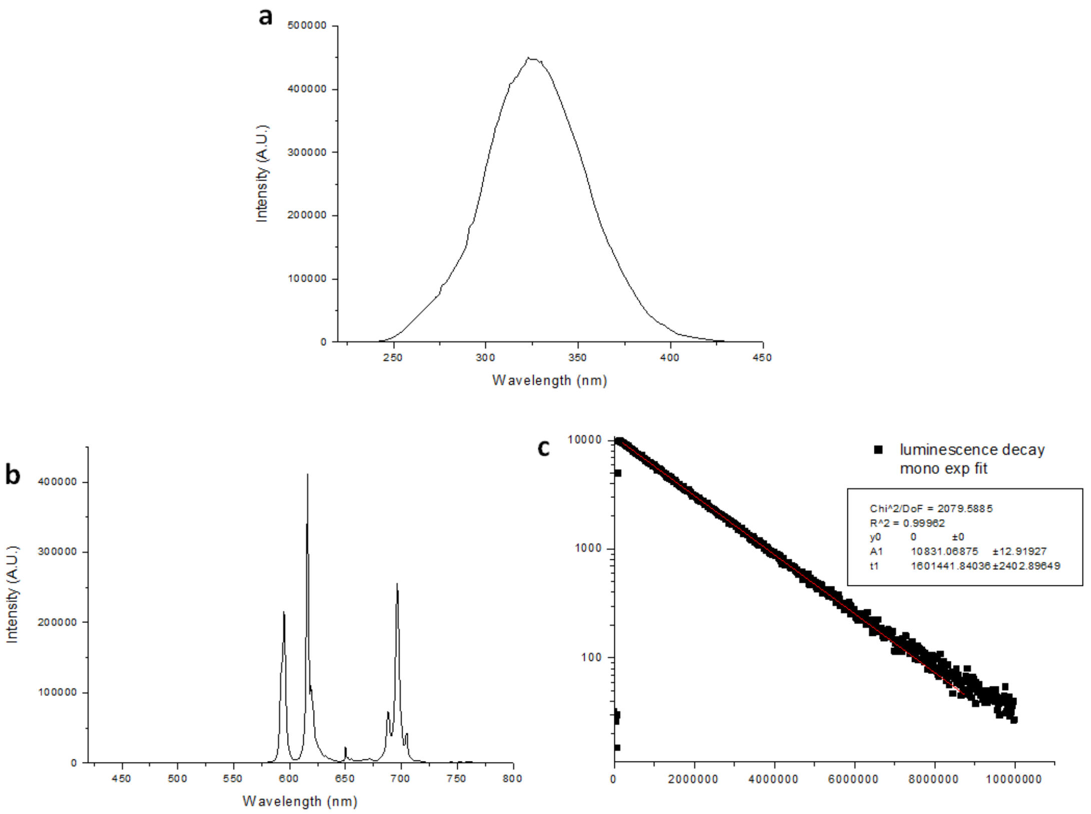

Supplementary Figure 42| Luminescent data of $[Eu_4(L3^{RR})_6](OTf)_{12}$ (1.50 x 10^{-5} M in MeCN). (**a**) Excitation spectrum, λem = 616 nm, slits = 1-0.5, filter 395 nm. (**b**) Emission spectrum, λex = 337 nm, slits = 1-0.5, filter 395 nm. (**c**) Excited state decay curve with mono-exponential fit, λem = 616 nm, slits = 3-2, filter 395 nm.

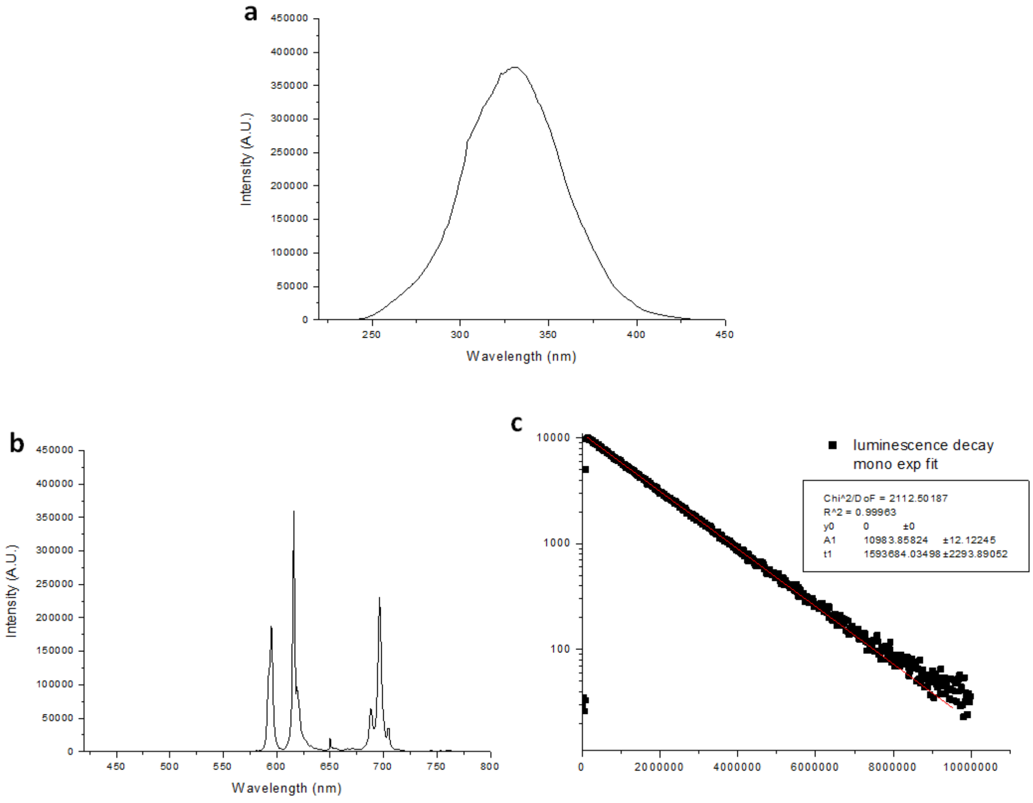

Supplementary Figure 43| Luminescent data of $[Eu_4(L3^{SS})_6](OTf)_{12}$ (1.50 x 10^{-5} M in MeCN). (**a**) Excitation spectrum, λem = 616 nm, slits = 1-0.5, filter 395 nm. (**b**) Emission spectrum, λex = 337 nm, slits = 1-0.5, filter 395 nm. (**c**) Excited state decay curve with mono-exponential fit, λem = 616 nm, slits = 3-2, filter 395 nm.

Supplementary Table 3. A summary of selected photophysical properties, UV-Vis absorption and luminescence data of $[Ln_2(\mathbf{L})_3](CF_3SO_3)_6$ in acetonitrile solution[a].

	λ_{abs}^{max} (nm)	ε^{max} ($L\cdot mol^{-1}\cdot cm^{-1}$)	λ_{em}^{max} (nm)	ϕ_X^{b}	ϕ_X^{c}	τ (ms)
**$[Eu_4(L1^{RR})_6](CF_3SO_3)_6$**	315	269400	616	0.17(1)	0.18(1)	1.63(2)
**$[Eu_4(L1^{SS})_6](CF_3SO_3)_6$**	315	272800	616	0.16(1)	0.15(1)	1.63(3)
**$[Eu_4(L2^{RR})_6](CF_3SO_3)_6$**	315	252900	616	0.18(1)	0.17(2)	1.59(3)
**$[Eu_4(L2^{SS})_6](CF_3SO_3)_6$**	315	263900	616	0.18(1)	0.19(2)	1.58(1)
**$[Eu_4(L3^{RR})_6](CF_3SO_3)_6$**	315	263000	616	0.18(1)	0.16(2)	1.62(1)
**$[Eu_4(L3^{SS})_6](CF_3SO_3)_6$**	315	279300	616	0.18(1)	0.18(1)	1.62(2)
**$[Gd_4(L1^{SS})_6](CF_3SO_3)_6$**	318	-	547[b]	-	-	0.003[d]

[a]Using a 1mm cuvette and filter 395 nm. [b]The relative quantum yields were referenced with quinine sulfate in 0.1 M sulfuric acid (ϕ = 0.577, λex = 350nm) with 10mm cuvette. The numbers in the parenthesis are standard deviations. [c]The relative quantum yields

were referenced with $Cs_3[Eu(dpa)_3]$ in 0.1 M TRIS-HCl (ϕ = 0.240, λex = 279nm) with 10mm cuvette. [d]Measurement performed at 77 K in 1:4 of MeOH/EtOH.

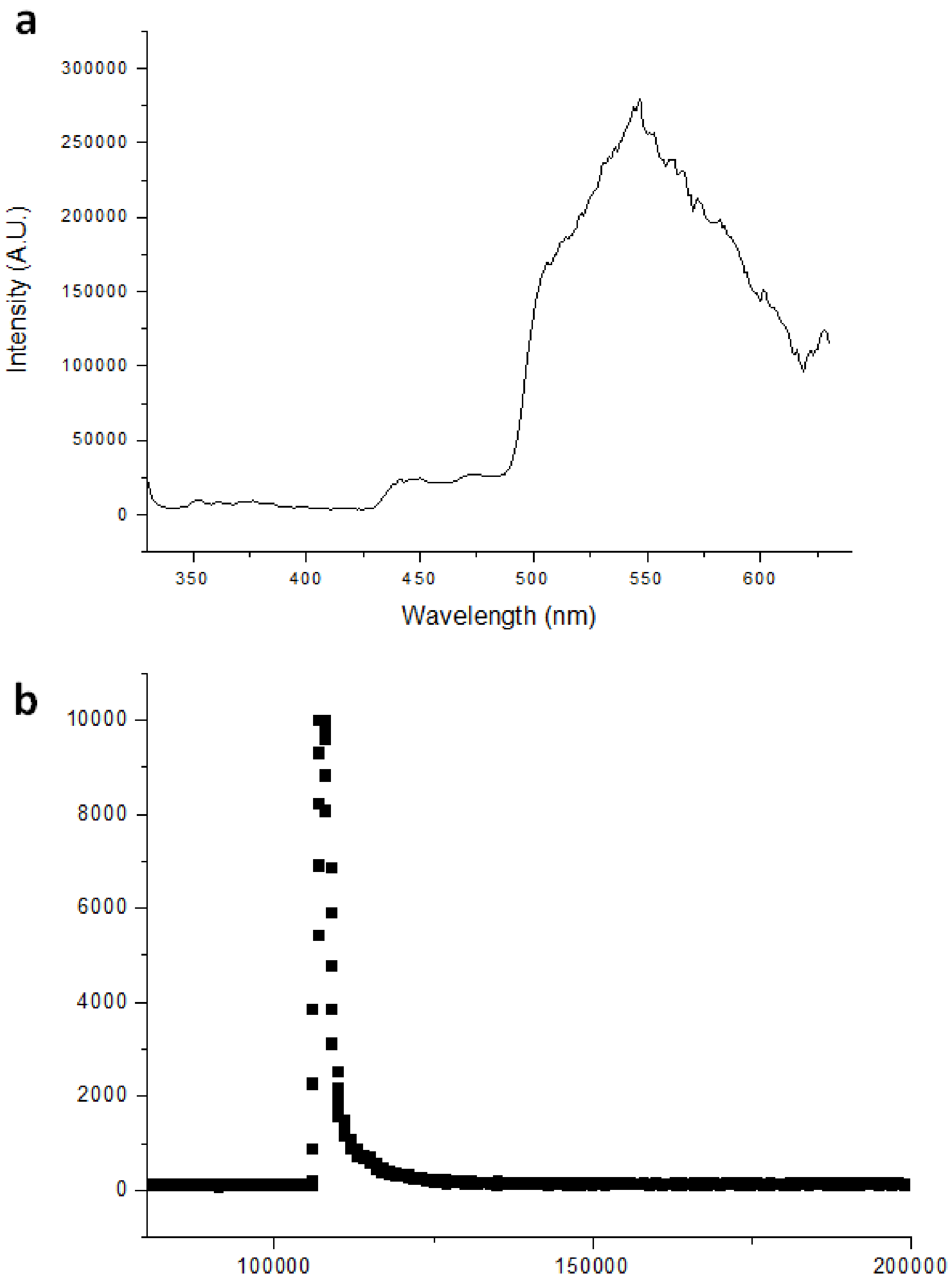

Supplementary Figure 44 | Emission and Excited state decay curve of $[Gd_4(L1^{SS})_6](OTf)_{12}$ (2.55 x 10^{-6} M, 1:4 of MeOH/EtOH, at 77K, under excitation at 320 nm.

a

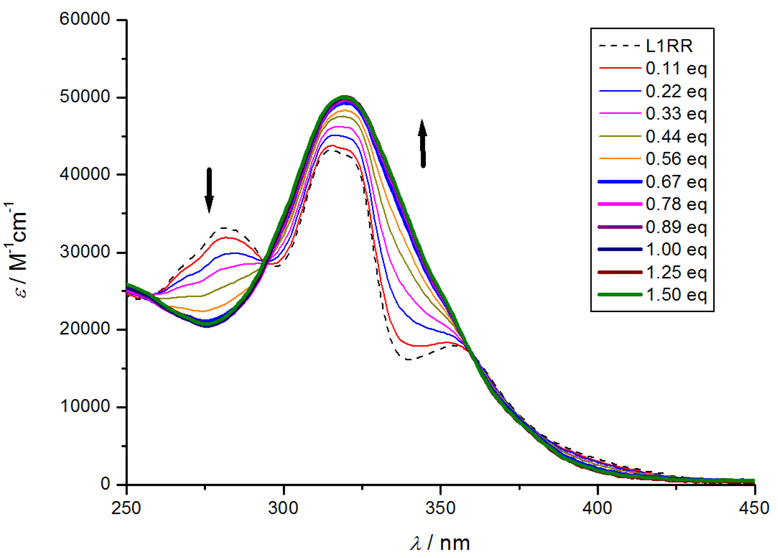

b

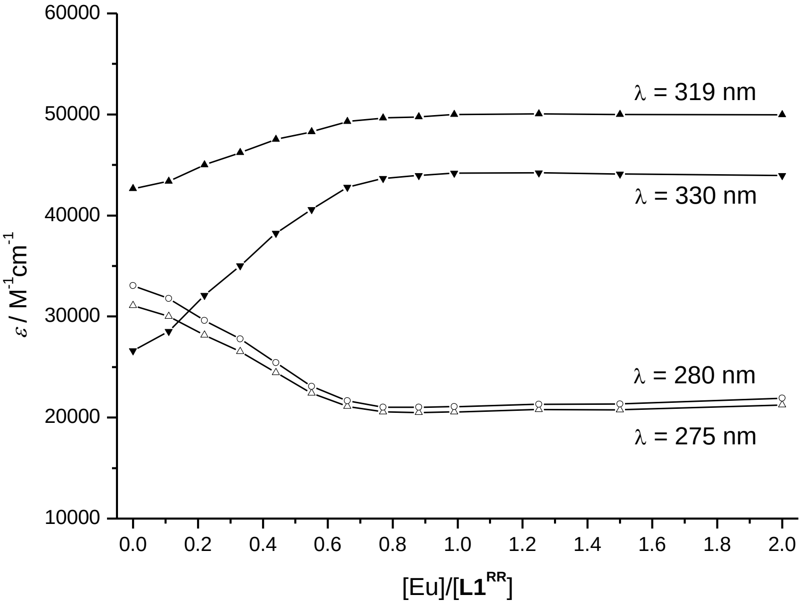

Supplementary Figure 45| UV-Vis titration of L1 with Eu(OTf)$_3$. (**a**) Variation in UV-Vis absorption spectra of titrating **L1^{RR}** (1.23 x 10^{-4}M, in 73:24:3, v/v/v, of $CHCl_3$/MeCN/MeOH) with Eu(OTf)$_3$ (0.027M in MeOH) at 298K (Eu:**L1^{RR}** = 0.0–2.0). (**b**) Variation of molar extinction coefficients at four different wavelengths upon titrating **L1^{RR}** with Eu(OTf)$_3$.

a

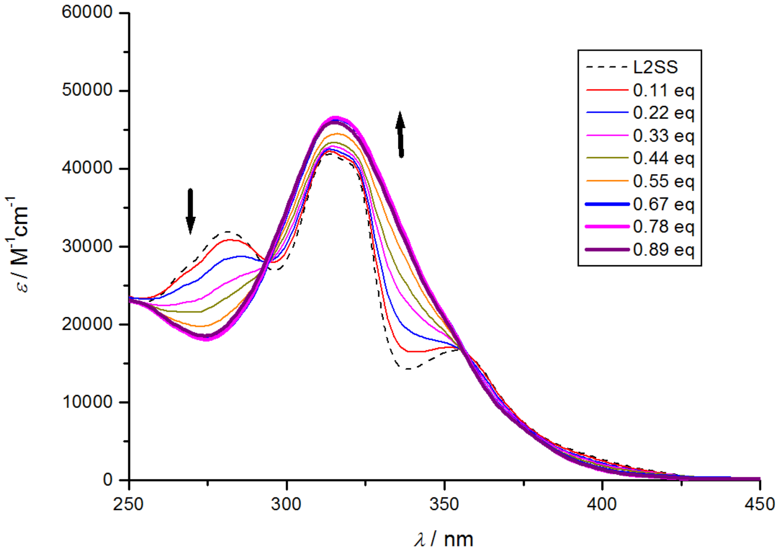

b

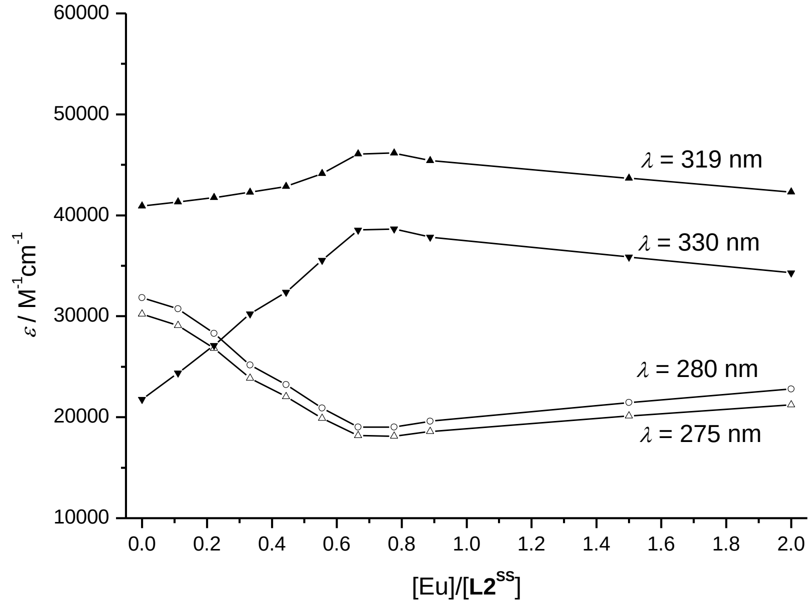

Supplementary Figure 46| UV-Vis titration of L2 with Eu(OTf)$_3$. (**a**) Variation in UV-Vis absorption spectra of titrating **L2^{SS}** (2.46 x 10^{-4}M, in 62:35:3, v/v/v, of $CHCl_3$/MeCN/MeOH) with Eu(OTf)$_3$ (0.027M in MeOH) at 298K (Eu:**L2^{SS}** = 0.0–2.0). (**b**) Variation of molar extinction coefficients at four different wavelengths upon titrating

L2^{SS} with Eu(OTf)$_3$.

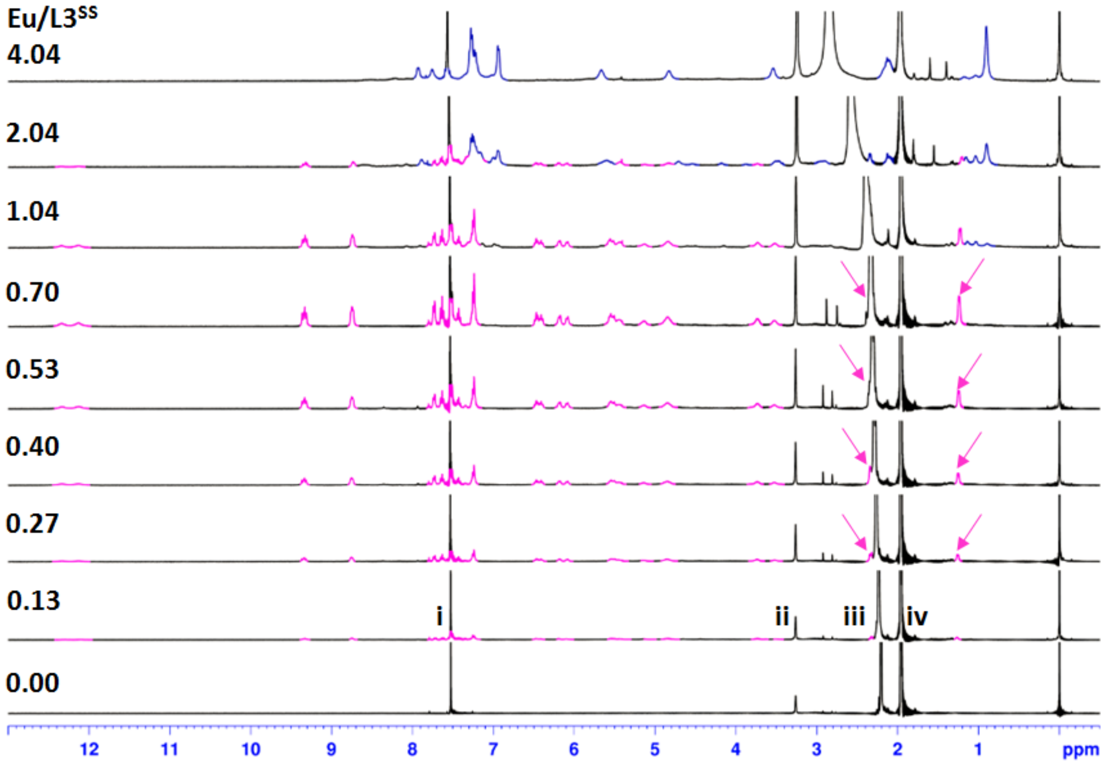

Supplementary Figure 47| ^{1}H NMR titrations of L1 or L2 with Eu(OTf)$_3$ showing the proposed supramolecular tetrahedral cage was formed. Variation in ^{1}H NMR spectra of titrating **L3^{SS}** suspension (1.36 x 10^{-3} M in 39:59:2, v/v/v, of CD_2Cl_2/CD_3CN/CD_3OD) with Eu(OTf)$_3$ (0.068 M in CD_3OD) at 298K. The reaction mixture become clear solution after addition of 0.7 eq. of Eu(OTf)$_3$. (Tetrahedral cage is shown in magenta. New species is shown in blue. Solid arrows indicate CH_3- from the cage; Peaks that are marked as **i**, **ii**, **iii**, **iv** are from the residual solvents of $CHCl_3$, MeOH, H_2O and MeCN, respectively.)

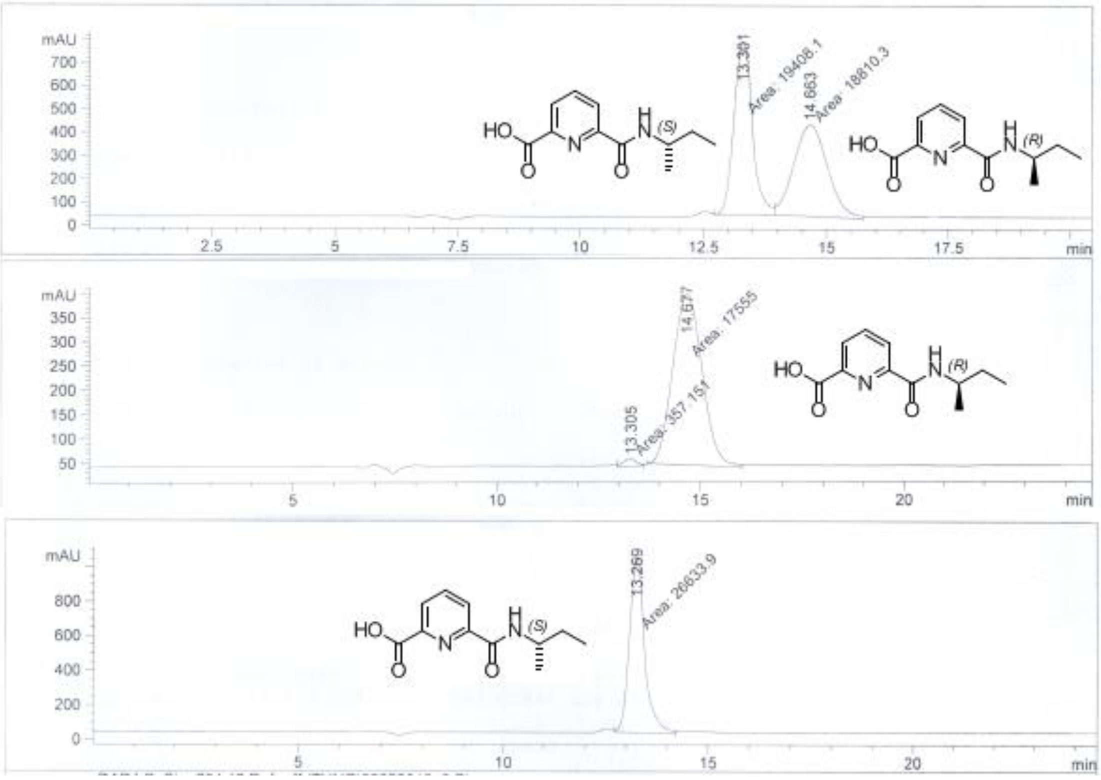

Supplementary Figure 48| HPLC spectra of intermediates 2^R and 2^S.

Supplementary Methods

(*R*)-6-(sec-butylcarbamoyl)picolinic acid 2^R and (*S*)-6-(sec-butylcarbamoyl)picolinic acid 2^S

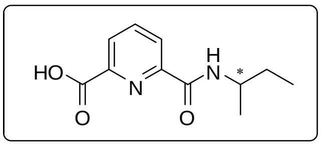

To a stirred solution of 2,6-pyridinedicarboxylic acid (5.00 g, 30.0 mmol, 2.5 equiv.) in anhydrous DMF (68 mL) at room temperature, HATU (3.79 g, 9.97 mmol, 1 equiv.) was added by five portions over 5 min under nitrogen. After allowing it to stir for 20 min, a (*R*)-(–)-sec-butylamine (1.01 mL, 9.97 mmol, 1.0 equiv.) was added dropwisely and the reaction mixture was allowed to stir for 20 min. DIPEA (3.81 mL, 21.9 mmol, 2.2 equiv.) was then added to the reaction mixture over 5 min and the resulting solution was stirred at room temperature for 14 h. The reaction mixture was then diluted with H_2O (100 mL), and extracted with DCM (5 × 30 mL), dried with $MgSO_4$, filtered, and concentrated *in vacuo*. The resulting residue was purified with flash column chromatography (with gradient from pure DCM to DCM/MeOH) to give a white solid. $\mathbf{2^R}$: (0.91 g, 4.09 mmol, 41% yield), ^{1}H NMR (400 MHz, CD_3OD, one COO*H* is missing due to their exchange with d-solvent, δ): 0.92 (t, *J* = 8 Hz, 3H), 1.23 (d, 3H), 1.58–1.63 (m, 2H), 4.03–4.00 (m, 1H), 8.12 (t, *J* = 8 Hz, 1H), 8.29–8.25 (m, 2H), 9.15 (d, *J* = 7 Hz, 1H). ^{13}C NMR (CD_3OD, δ): 12.01, 21.40, 31.31, 49.50, 127.58, 129.28, 141.39, 148.86, 152.40, 165.90, 168.50. The enantiomeric purity was determined with HPLC with AS-H column (Hexane/*i*-propanol: 80/20, 0.1% TFA; flow rate: 0.5 ml/min) and compared with a racemic mixture according to the elution orders with retention times, t_S = 13.30 min and t_R = 14.66 min) to be > 99% ee. $\mathbf{2^S}$ was isolated, following the procedure for $\mathbf{2^R}$ with the use of (*R*)-(–)-sec-butylamine instead, in 38% yield (0.84 g, 3.79 mmol): ^{1}H NMR (400 MHz, CD_3OD, one COO*H* is missing due to their exchange with d-solvent, δ): 0.92 (d, *J* = 8 Hz, 3H), 1.23 (d, *J* = 6.8 Hz, 3H), 1.56–1.65 (m, 2H), 3.98–4.03 (m, 1H), 8.11 (t, *J* = 8 Hz, 1H), 8.25–8.29 (m, 2H), 9.14 (d, *J* = 7 Hz, 1H). ^{13}C NMR (CD_3OD, δ): 11.99, 21.38, 31.29, 49.48, 127.57, 129.26, 141.37, 148.82, 152.38, 165.88, 168.47. The enantiomeric purity was determined to be > 98% ee.

N^2,$N^{2'}$-(9,10-dioxo-9,10-dihydroanthracene-2,6-diyl)bis{N^6-[(*R*)-(1-phenylethyl)pyridine-2,6-dicarboxamide]} ($\mathbf{L1^{RR}}$) and

N^2,$N^{2'}$-(9,10-dioxo-9,10-dihydroanthracene-2,6-diyl)bis{N^6-[(*S*)-(1-phenylethyl)pyridine-2,6-dicarboxamide]} (L1^{SS})

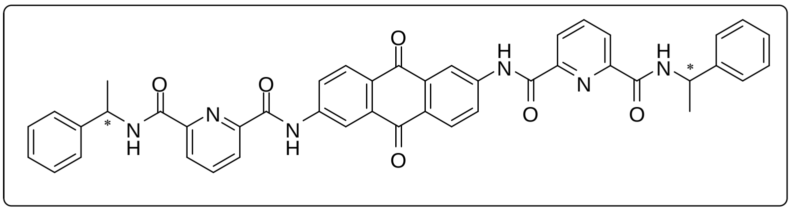

To a stirred solution of **1**^{R} (0.50 g, 1.84 mmol, 2.2 equiv.) in anhydrous DMF (16 mL) at room temperature, HATU (0.95 g, 2.50 mmol, 3.0 equiv.) was added under nitrogen. After allowing it to stir for 20 min, a 2,6-diaminoanthraquinone (0.17 g, 0.83 mmol, 1.0 equiv.) was added and the reaction mixture was allowed to stir for 20 min in dark. DIPEA (0.87 mL, 4.98 mmol, 6.0 equiv.) was then added to the reaction mixture and the resulting solution was stirred at room temperature for 12 h. The reaction mixture was then diluted with H_2O (50 mL) and extracted with DCM (5 × 30 mL). After removing all of the organic volatile under reduced pressure, the residue was diluted with ethyl acetate (50 mL) and then washed with H_2O (5 × 30 mL) to remove the DMF residual. The organic layer was separated and then concentrated directly under reduced pressure. The residue was then diluted with MeCN (20 mL), and fine powder was progressively precipitated out. Then the solid was collected by filtration and the desired compound was isolated. (**L1**^{RR}): (0.46 g, 0.61 mmol, 74% yield), ^{1}H NMR (400 MHz, $CDCl_3$/CD_3OD-10:1, δ): 1.75 (d, *J* = 8 Hz, 6H), 5.46 (q, *J* = 8 Hz, 2H), 7.30 (t, *J* = 8 Hz, 2H), 7.40 (t, *J* = 8 Hz, 6H), 7.51 (d, *J* = 8 Hz, 4H), 8.12 (t, *J* = 8 Hz, 2H), 8.28–8.40 (m, 4H), 8.42 (d, *J* = 8 Hz, 2H), 8.46 (d, *J* = 8 Hz, 2H), 8.65 (d, *J* = 8 Hz, 2H), 9.47 (d, *J* = 8 Hz, 2H). ^{13}C NMR ($CDCl_3$/CD_3OD-10:1, δ): 20.80, 46.94, 117.75, 125.19, 125.43, 125.90, 126.03, 127.12, 128.42, 129.06, 129.21, 134.44, 139.00, 143.01, 143.71, 148.29, 149.30, 162.74, 163.17, 182.42. (**L1**^{SS}) was synthesized, following the procedure for **(L1**^{RR}**)** with the use of **1**^{S} instead, in 79% yield (0.49 g, 0.66 mmol): ^{1}H NMR (400 MHz, $CDCl_3$/CD_3OD-10:1, δ): 1.76 (d, *J* = 8 Hz, 6H), 5.47 (q, *J* = 8 Hz, 2H), 7.30 (t, *J* = 8 Hz, 2H), 7.41 (t, *J* = 8 Hz, 6H), 7.51 (d, *J* = 8 Hz, 4H), 8.14 (t, *J* = 8 Hz, 2H), 8.30–8.40 (m, 4H), 8.42 (d, *J* = 8 Hz, 2H), 8.47 (d, *J* = 8 Hz, 2H), 8.63 (d, *J* = 8 Hz, 2H), 9.54 (d, *J* = 8 Hz, 2H). ^{13}C NMR ($CDCl_3$/CD_3OD-10:1, δ): 20.72, 47.40, 117.80, 125.19, 125.38, 125.82, 125.96, 127.03, 128.35, 128.95, 129.20, 134.41, 138.96, 142.97, 143.66, 148.28, 149.24, 162.75, 163.20, 182.37.

Hexa{μ-[N^2,$N^{2'}$-(9,10-dioxo-9,10-dihydroanthracene-2,6-diyl)bis{N^6-[(*R*)-(1-phenylethyl)pyridine-2,6-dicarboxamide]}]}tetraeuropium(III) dodecatriflate,

[Eu$_4$(L1^{RR})$_6$](CF$_3$SO$_3$)$_{12}$ and

Hexa{μ-[*N*^{2},*N*$^{2'}$-(9,10-dioxo-9,10-dihydroanthracene-2,6-diyl)bis{*N*^{6}-[(*S*)-(1-phenylethyl)pyridine-2,6-dicarboxamide]}]}tetraeuropium(III) dodecatriflate, [Eu$_4$(L1^{SS})$_6$](CF$_3$SO$_3$)$_{12}$

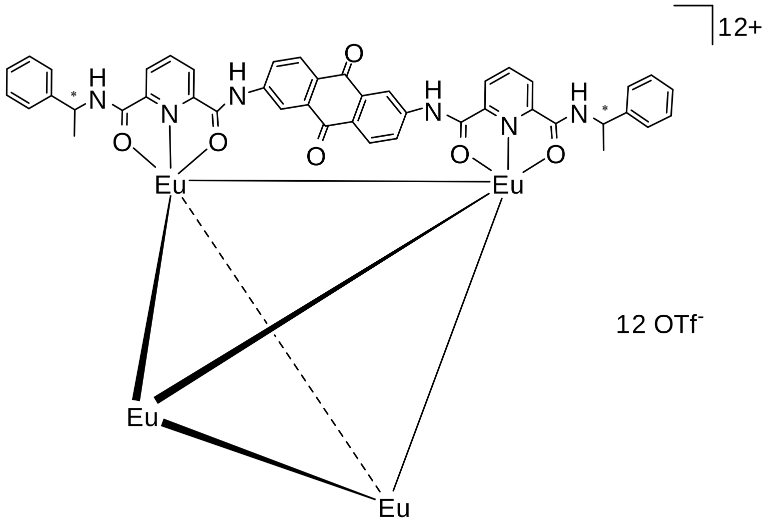

To a white suspension of (**L1^{RR}**) (0.100 g, 0.135 mmol, 1.5 equiv.) in a mixture of 12 mL DCM/MeOH (12:1, v/v), a solution of Eu(CF$_3$SO$_3$)$_3$ (0.054 g, 0.090 mmol, 1 equiv.) in 16 mL MeCN was added. The suspension was changed to yellow turbidity immediately. The reaction mixture was then refluxed for 1 h and the solid progressively dissolved to give a resulting homogeneous yellow solution. The solvent was removed under reduced pressure. Then crude product was re-dissolved in MeCN and then recrystallized by slow diffusion of diethyl ether to give the desired product. **[Eu$_4$(L1^{RR})$_6$](CF$_3$SO$_3$)$_{12}$**: (0.136 g, 0.020 mmol, 88% yield). ^{1}H NMR (400 MHz, CD$_3$CN, δ): 2.44 (d, br., *J* = 4 Hz, 6 × 6H, C*H$_3$*), 4.28 (s, br., 6 × 2H, N*H*), 5.54 (d, br., *J* = 8 Hz, 6 × 2H), 5.67 (d, br., *J* = 8 Hz, 6 × 2H), 6.29 (t, br., *J* = 8 Hz, 6 × 2H), 6.58–6.68 (m, br., 6 × 10H, phenyl-*H*), 7.03 (s, br., 6 × 2H, N*H*), 7.86 (s, br. 6 × 2H, CH$_3$C*H*), 8.62 (d, br., *J* = 8 Hz, 6 × 2H), 9.29 (d, br., *J* = 8 Hz, 6 × 2H), 12.36 (s, br., 6 × 2H). ^{13}C NMR (CD$_3$CN, δ): 23.98 (*C*H$_3$), 53.57 (CH$_3$*C*H), 93.52 (*C*H), 94.67 (*C*H), 124.04, 124.12 (*C*H), 126.47 (*C*H, phenyl-*C*), 128.62 (*C*H, phenyl-*C*), 129.18 (*C*H), 129.59 (*C*H, phenyl-*C*), 131.00 (*C*H), 134.26, 135.38, 137.16, 139.58, 144.54, 155.03 (*C*H), 157.07, 165.85, 184.46. HRMS (ESI) calcd. for C$_{272}$H$_{204}$Eu$_4$F$_{24}$N$_{36}$O$_{60}$S$_8$ [M − 4OTf$^-$]: 1564.2071, found 1564.2142. Calculated for C$_{276}$H$_{204}$N$_{36}$O$_{72}$Eu$_4$F$_{36}$S$_{12}$·19H$_2$O: C, 46.07; H, 3.39; N, 7.01 %; Found: C, 46.61; H, 3.39; N, 7.01%. mp: 341–345 °C (decomposition); **[Eu$_4$(L1^{SS})$_6$](CF$_3$SO$_3$)$_{12}$** was synthesized, following the procedure for **[Eu$_4$(L1^{RR})$_6$](CF$_3$SO$_3$)$_{12}$** with the use of (**L1^{SS}**) instead, in 90% yield (0.139 g, 0.020 mmol): ^{1}H NMR (400 MHz, CD$_3$CN, δ): 2.44 (d, br., *J* = 4 Hz, 6 × 6H, **C*H$_3$***), 4.31 (s, br., 6 × 2H, **N*H***), 5.55 (d, br., *J* = 8 Hz, 6 × 2H), 5.69 (d, br., *J* = 8 Hz, 6 × 2H), 6.29 (t, br., *J* = 8 Hz, 6 × 2H), 6.56–6.70 (m, br., 6 × 10H,

phenyl-*H*), 7.05 (s, br., 6 × 2H, **N*H***), 7.87 (s, br., 2H, **CH_3C*H***), 8.62 (d, br., *J* = 8 Hz, 6 × 2H), 9.29 (d, br., J = 8 Hz, 6 × 2H), 12.37 (s, br., 6 × 2H). ^{13}C NMR (CD_3CN, δ): 23.30 (***C*H₃**), 52.86 (**CH_3*C*H**), 92.65 (*C*H), 93.81 (*C*H), 123.33, 123.48 (*C*H), 125.75 (*C*H, **phenyl-*C***), 127.91 (*C*H, **phenyl-*C***), 128.51 (*C*H), 128.88 (*C*H, **phenyl-*C***), 130.32 (*C*H), 133.58, 134.52, 136.49, 138.75, 143.91, 154.36 (*C*H), 156.32, 165.11, 183.80. HRMS (ESI) calcd. for $C_{272}H_{204}Eu_4F_{24}N_{36}O_{60}S_8$ [M – 4OTf$^-$]: 1564.2071, found 1564.2089. Calculated for $C_{276}H_{204}N_{36}O_{72}Eu_4F_{36}S_{12}\cdot19H_2O$: C, 46.07; H, 3.39; N, 7.01 %; Found: C, 46.74; H, 3.37; N, 6.96 %. mp: 341–348 °C (decomposition).

Hexa{μ-[*N*^{2},*N*$^{2'}$-(9,10-dioxo-9,10-dihydroanthracene-2,6-diyl)bis{*N*^{6}-[(*R*)-(1-phenylethyl)pyridine-2,6-dicarboxamide]}]}tetrayttrium(III) dodecatriflate, [$Y_4(L1^{RR})_6](CF_3SO_3)_{12}$ and
Hexa{μ-[*N*^{2},*N*$^{2'}$-(9,10-dioxo-9,10-dihydroanthracene-2,6-diyl)bis{*N*^{6}-[(*S*)-(1-phenylethyl)pyridine-2,6-dicarboxamide]}]}tetrayttrium(III) dodecatriflate, [$Y_4(L1^{SS})_6](CF_3SO_3)_{12}$

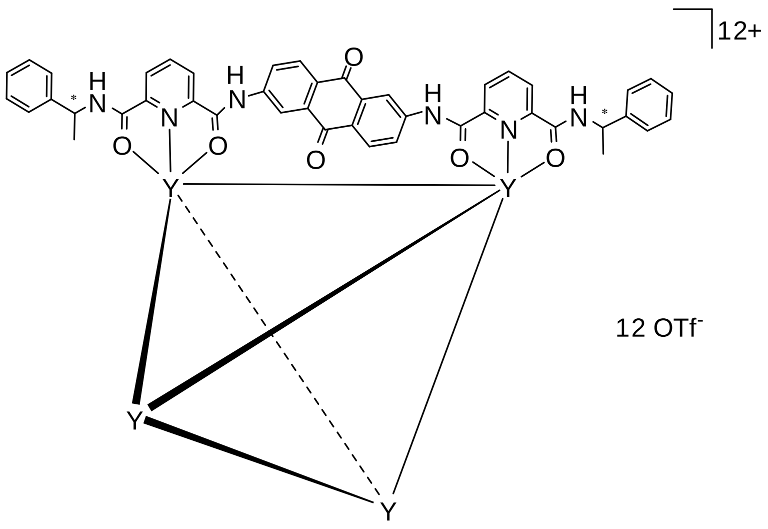

To a white suspension of (**L1^{RR}**) (0.030 g, 0.040 mmol, 1.5 equiv.) in a mixture of 6 mL DCM/MeOH (12:1, v/v), a solution of $Y(CF_3SO_3)_3$ (0.014 g, 0.027 mmol, 1 equiv.) in 12 mL MeCN was added. The suspension was changed to yellow turbidity gradually. The reaction mixture was then refluxed for 1 h and the solid progressively dissolved to give a resulting homogeneous yellow solution. The solvent was removed under reduced pressure. Then crude product was re-dissolved in MeCN and then recrystallized by slow diffusion of diethyl ether to give the desired product. **[$Y_4(L1^{RR})_6](CF_3SO_3)_{12}$**: (0.038 g, 0.0057 mmol, 85% yield). ^{1}H NMR (400 MHz, CD_3CN, δ): 1.62 (d, *J* = 8 Hz, 6 × 6H, *CH₃*), 4.90–5.02 (m, 6 × 2H, CH_3C*H*), 6.97 (d, *J* = 8 Hz, 6 × 4H, phenyl-*H*), 7.17 (t, *J* = 8 Hz, 6 × 4H, phenyl-*H*), 7.25 (t, *J* = 8 Hz, 6 × 2H, phenyl-*H*),

7.94 (d, *J* = 8 Hz, 6 × 2H), 8.03 (d, *J* = 8 Hz, 6 × 2H), 8.27 (d, *J* = 4 Hz, 6 × 2H), 8.37 (t, *J* = 8 Hz, 6 × 2H), 8.46 (d, *J* = 8 Hz, 6 × 2H), 8.75 (d, *J* = 8 Hz, 6 × 2H), 9.07 (d, *J* = 8 Hz, 6 × 2H, N*H*), 10.62 (s, 6 × 2H, N*H*). ^{13}C NMR (CD_3CN, δ): 22.22 (CH_3), 53.61 (CH_3CH), 118.99 (*C*H, it is buried underneath the residual acetonitrile peak), 126.83 (*C*H, phenyl-*C*), 127.63 (*C*H), 128.05 (*C*H), 128.48 (*C*H), 129.06 (*C*H, phenyl-*C*), 129.92 (*C*H), 130.02 (*C*H, phenyl-*C*), 131.79, 135.05, 142.97, 143.54, 144.29 (*C*H), 147.75, 148.63, 168.08, 168.37, 182.41. HRMS (ESI) calcd. for $C_{272}H_{204}Y_4F_{24}N_{36}O_{60}S_8$ [M − 4OTf$^-$]: 1500.1903, found 1500.1854. Calculated for $C_{276}H_{204}N_{36}O_{72}Y_4F_{36}S_{12}\cdot 19H_2O$: C, 47.74; H, 3.51; N, 7.26 %; Found: C, 48.74; H, 3.57; N, 7.19 %. mp: 331–338 ^{o}C (decomposition); **$[Y_4(L1^{SS})_6](CF_3SO_3)_{12}$** was synthesized, following the procedure for **$[Y_4(L1^{RR})_6](CF_3SO_3)_{12}$** with the use of (**L1^{SS}**) instead: (0.040 g, 0.0061 mmol, 91% yield) ^{1}H NMR (400 MHz, CD_3CN, δ): 1.62 (d, *J* = 8 Hz, 6 × 6H, CH_3), 4.90–5.02 (m, 6 × 2H, CH_3CH), 6.97 (d, *J* = 8 Hz, 6 × 4H, phenyl-*H*), 7.17 (t, *J* = 8 Hz, 6 × 4H, phenyl-*H*), 7.25 (t, *J* = 8 Hz, 6 × 2H, phenyl-*H*), 7.94 (d, *J* = 8 Hz, 6 × 2H), 8.03 (d, *J* = 8 Hz, 6 × 2H), 8.27 (d, *J* = 4 Hz, 6 × 2H), 8.37 (t, *J* = 8 Hz, 6 × 2H), 8.47 (d, *J* = 8 Hz, 6 × 2H), 8.75 (d, *J* = 8 Hz, 6 × 2H), 9.09 (d, *J* = 8 Hz, 6 × 2H, N*H*), 10.64 (s, 6 × 2H, N*H*). ^{13}C NMR (CD_3CN, δ): 22.24 (CH_3), 53.63 (CH_3CH), 119.01 (*C*H, it is buried underneath the residual acetonitrile peak), 126.87 (*C*H, phenyl-*C*), 127.66 (*C*H), 128.07 (*C*H), 128.48 (*C*H), 129.10 (*C*H, phenyl-*C*), 129.95 (*C*H), 130.06 (*C*H, phenyl-*C*), 131.82, 135.07, 143.01, 143.53, 144.32 (*C*H), 147.79, 148.67, 168.10, 168.43, 182.47. HRMS (ESI) calcd. for $C_{272}H_{204}Y_4F_{24}N_{36}O_{60}S_8$ [M − 4OTf$^-$]: 1500.1903, found 1500.1832. Calculated for $C_{276}H_{204}N_{36}O_{72}Y_4F_{36}S_{12}\cdot 21H_2O$: C, 47.49; H, 3.55; N, 7.22 %; Found: C, 48.62; H, 3.63; N, 7.15 %. mp: 333–339 ^{o}C (decomposition)

N^2,$N^{2'}$-(9,10-dioxo-9,10-dihydroanthracene-2,6-diyl)bis{N^6-[(*R*)-(*sec*-butyl)pyridine-2,6-dicarboxamide]} (L2^{RR}) and N^2,$N^{2'}$-(9,10-dioxo-9,10-dihydroanthracene-2,6-diyl)bis{N^6-[(*S*)-(*sec*-butyl)pyridine-2,6-dicarboxamide]} (L2^{SS})

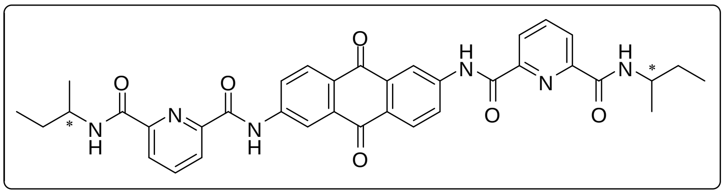

To a stirred solution of **2^R** (0.15 g, 0.68 mmol, 2.2 equiv.) in anhydrous DMF (3 mL) at room temperature, HATU (0.37 g, 0.97 mmol, 3.0 equiv.) was added under nitrogen. After allowing it to stir for 20 min, a 2,6-diaminoanthraquinone (0.06 g,

0.31 mmol, 1.0 equiv.) was added and the reaction mixture was allowed to stir for 20 min in dark. DIPEA (0.33 mL, 1.86 mmol, 6.0 equiv.) was then added to the reaction mixture and the resulting solution was stirred at room temperature for 12 h. The reaction mixture was then diluted with H_2O (20 mL) and extracted with DCM (5 × 20 mL). After removing all of the organic volatile under reduced pressure, the residue was diluted with ethyl acetate (30 mL) and then washed with H_2O (5 × 20 mL) to remove the DMF residual. The organic layer was separated and then concentrated directly under reduced pressure. The residue was then diluted with MeCN (15 mL), and fine powder was progressively precipitated out. Then the solid was collected by filtration and the desired compound was isolated. (**L2^{RR}**): (0.13 g, 0.20 mmol, 65% yield), ^{1}H NMR (400 MHz, $CDCl_3$/CD_3OD-10:1, two N*Hs* are missing due to solvent exchange, δ): 1.05 (t, *J* = 7 Hz, 6H), 1.38 (d, *J* = 6 Hz, 6H), 1.67–1.80 (m, 4H), 4.20–4.25 (m, 2H), 8.13 (t, *J* = 8 Hz, 2H), 8.31 (d, *J* = 2 Hz, 2H), 8.40 (d, *J* = 9 Hz, 2H), 8.44 (d, *J* = 8 Hz, 2H), 8.47 (d, *J* = 8 Hz, 2H), 8.76 (dd, *J* = 9, 2 Hz, 2H), 8.93 (d, *J* = 9 Hz, 2H). ^{13}C NMR ($CDCl_3$/CD_3OD-10:1, δ): 10.85, 20.20, 29.60, 47.32, 117.79, 125.32, 125.55, 126.16, 129.42, 129.55, 134.73, 139.24, 144.02, 148.40, 149.86, 162.87, 163.29, 182.74. (**L2^{SS}**) was synthesized, following the procedure for (**L2^{RR}**) with the use of **2^S** instead, in 61% yield (0.12 g, 0.19 mmol): ^{1}H NMR (400 MHz, $CDCl_3$/CD_3OD-10:1, two N*Hs* are missing due to solvent exchange, δ): 1.05 (d, *J* = 7 Hz, 6H), 1.38 (d, *J* = 6 Hz, 6H), 1.67–1.80 (m, 4H), 4.17–4.27 (m, 2H), 8.13 (t, *J* = 8 Hz, 2H), 8.31 (d, *J* = 2 Hz, 2H), 8.40 (d, *J* = 9 Hz, 2H), 8.45 (d, *J* = 8 Hz, 2H), 8.48 (d, *J* = 8 Hz, 2H), 8.78 (dd, *J* = 9, 2 Hz, 2H), 8.88 (d, *J* = 9 Hz, 2H). ^{13}C NMR ($CDCl_3$/CD_3OD-10:1, δ): 10.66, 19.98, 29.39, 47.27, 117.71, 125.21, 125.35, 125.92, 129.23, 129.28, 134.52, 139.03, 143.87, 148.24, 149.66, 162.79, 163.29, 182.58.

Hexa{μ-[*N*^{2},*N*$^{2'}$-(9,10-dioxo-9,10-dihydroanthracene-2,6-diyl) bis{*N*^{6}-[(*R*)-(*sec*-butyl)pyridine-2,6-dicarboxamide]}]}tetraeuropium(III) dodecatriflate, [Eu_4(L2^{RR})$_6$](CF_3SO_3)$_{12}$ and Hexa{μ-[*N*^{2},*N*$^{2'}$-(9,10-dioxo-9,10-dihydroanthracene-2,6-diyl) bis{*N*^{6}-[(*R*)-(*sec*-butyl)pyridine-2,6-dicarboxamide]}]}tetraeuropium(III) dodecatriflate, [Eu_4(L2^{SS})$_6$](CF_3SO_3)$_{12}$

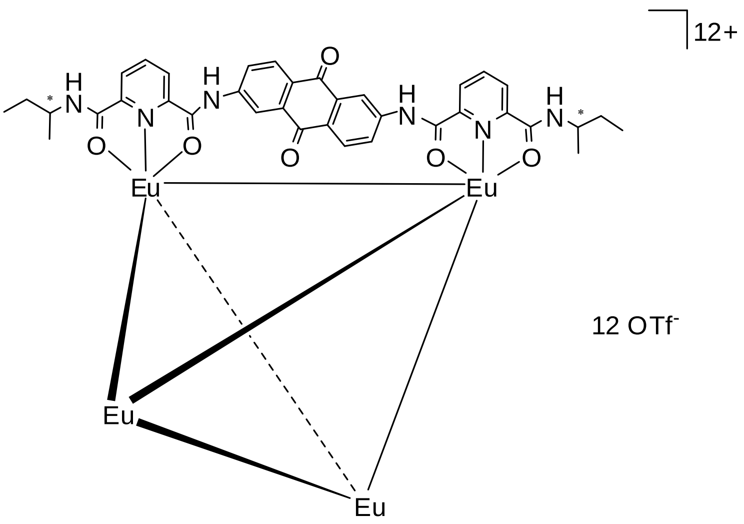

To a white suspension of (**L2^{RR}**) (0.04 g, 0.062 mmol, 1.5 equiv.) in a mixture of 10 mL DCM/MeOH (12:1, v/v), a solution of $Eu(CF_3SO_3)_3$ (0.025 g, 0.041 mmol, 1 equiv.) in 12 mL MeCN was added. The suspension was changed to yellow turbidity immediately. The reaction mixture was then refluxed for 1 h and the solid progressively dissolved to give a resulting homogeneous yellow solution. The solvent was removed under reduced pressure. Then crude product was re-dissolved in MeCN and then recrystallized by slow diffusion of diethyl ether to give the desired product. **[Eu$_4$(L2^{RR})$_6$](CF$_3$SO$_3$)$_{12}$**: (0.045 g, 0.007 mmol, 70% yield). ^{1}H NMR (400 MHz, CD_3CN, some of the peaks show two sets of peaks, **A** and **B**, respectively in ~1.06:1 ratio, δ): 0.07 (t, br., *J* = 8 Hz, **6 × 6H, CH$_2$C*H$_3$*, A**), 0.93 (d, br., *J* = 4 Hz, **6 × 6H, CHC*H$_3$*, B**), 1.08–1.22 (m, br., **6 × 2H, CH*H*, A**), 1.38–1.53 (m, br., **6 × 2H, CH*H*, A**), 2.60 (d, br., *J* = 4 Hz, **6 × 6H, CHC*H$_3$*, A**), 2.83 (t, br., *J* = 8 Hz, **6 × 6H, CH$_2$C*H$_3$*, B**), 2.83–2.94 (m, br., **6 × 2H, C*H*H, B**), 3.05–3.18 (m, br., **6 × 2H, C*H*H, B**), 4.04–4.,19 (m, br., **6 × 2H, N*H*, A** and **6 × 2H, N*H*, B**), 5.48 (s, br., **6 × 2H, C*H*CH$_3$, B**), 5.64 (s, br., **6 × 2H, C*H*CH$_3$, A**), 5.85 (d, br., *J* = 8 Hz, **6 × 2H, A** and **6 × 2H, B**), 6.19 (d, br., *J* = 4 Hz, **6 × 2H, A** and **6 × 2H, B**), 6.59 (q, br., *J* = 8 Hz, **6 × 2H, A** and **6 × 2H, B**), 7.34 (s, br., **6 × 2H, N*H*, A** and **6 × 2H, N*H*, B**), 8.53–8.69 (m, br., **6 × 2H, A** and **6 × 2H, B**), 9.22 (t, br., *J* = 8 Hz, **6 × 2H, A** and **6 × 2H, B**), 11.94 (s, br., **6 × 2H, A**), 11.97 (s, br., **6 × 2H, B**). ^{13}C NMR (CD_3CN, some of the peaks are shown into two sets of peaks, δ): 10.57 (**CH$_2$*C*H$_3$, A**), 12.87 (**CH$_2$*C*H$_3$, B**), 20.39 (**CH*C*H$_3$, B**), 21.77 (**CH*C*H$_3$, A**), 30.45 (***C*H$_2$, A** and **B**), 31.69 (***C*H$_2$, A** and **B**), 51.09 (***C*HCH$_3$, B**), 51.25 (***C*HCH$_3$, A**), 92.62, 93.92, 123.29, 123.36, 128.58, 130.31, 133.51, 136.38, 136.43, 139.97, 140.53, 143.96, 144.02, 154.65, 154.74, 156.96, 157.34, 165.28, 183.72, 183.77. HRMS (ESI) calcd. for $C_{224}H_{204}Eu_4F_{24}N_{36}O_{60}S_8$ [M − 4OTf$^-$]: 1419.9566, found 1419.9639. Calculated for $C_{228}H_{204}N_{36}O_{72}Eu_4F_{36}S_{12}\cdot 23H_2O$: C, 40.93; H, 3.77; N, 7.54 %; Found: C, 41.09; H, 3.80; N, 7.51 %. mp: 363–371 °C (decomposition); **[Eu$_4$(L2^{SS})$_6$](CF$_3$SO$_3$)$_{12}$** was synthesized, following the procedure for

[Eu$_4$(L2^{RR})$_6$](CF$_3$SO$_3$)$_{12}$ with the use of (**L2^{SS}**) instead, in 76% yield (0.049 g, 0.008 mmol): ^{1}H NMR (400 MHz, CD$_3$CN, some of the peaks show two sets of peaks, **A** and **B**, respectively in ~1.06:1 ratio, δ): 0.08 (t, br., *J* = 8 Hz, **6 × 6H, CH$_2$C*H$_3$*, A**), 0.93 (d, br., *J* = 4 Hz, **6 × 6H, CHC*H$_3$*, B**), 1.08–1.22 (m, br., **6 × 2H, CH*H*, A**), 1.38–1.53 (m, br., **6 × 2H, CH*H*, A**), 2.60 (d, br., *J* = 4 Hz, **6 × 6H, CHC*H$_3$*, A**), 2.83 (t, br., *J* = 8 Hz, **6 × 6H, CH$_2$C*H$_3$*, B**), 2.83–2.94 (m, br., **6 × 2H, C*H*H, B**), 3.05–3.17 (m, br., **6 × 2H, C*H*H, B**), 4.04–4.,18 (m, br., **6 × 2H, N*H*, A** and **6 × 2H, N*H*, B**), 5.48 (s, br., **6 × 2H, C*H*CH$_3$, B**), 5.64 (s, br., **6 × 2H, C*H*CH$_3$, A**), 5.85 (d, br., *J* = 8 Hz, **6 × 2H, A** and **6 × 2H, B**), 6.19 (d, br., *J* = 4 Hz, **6 × 2H, A** and **6 × 2H, B**), 6.59 (q, br., *J* = 8 Hz, **6 × 2H, A** and **6 × 2H, B**), 7.35 (s, br., **6 × 2H, N*H*, A** and **6 × 2H, N*H*, B**), 8.53–8.69 (m, br., **6 × 2H, A** and **6 × 2H, B**), 9.22 (t, br., *J* = 8 Hz, **6 × 2H, A** and **6 × 2H, B**), 11.93 (s, br., **6 × 2H, A**), 11.98 (s, br., **6 × 2H, B**). ^{13}C NMR (CD$_3$CN, some of the peaks are shown into two sets of peaks, δ): 10.53 (**CH$_2$*C*H$_3$, A**), 12.84 (**CH$_2$*C*H$_3$, B**), 20.35 (**CH*C*H$_3$, B**), 21.73 (**CH*C*H$_3$, A**), 30.41 (***C*H$_2$, A** and **B**), 31.66 (***C*H$_2$, A** and **B**), 51.06 (***C*HCH$_3$, B**), 51.21 (***C*HCH$_3$, A**), 92.52, 93.90, 123.25, 123.33, 128.55, 130.27, 133.46, 136.35, 136.41, 140.02, 140.52, 143.92, 143.98, 154.62, 154.70, 156.91, 157.24, 165.25, 183.68, 183.72. HRMS (ESI) calcd. for $C_{224}H_{204}Eu_4F_{24}N_{36}O_{60}S_8$ [M − 4OTf$^-$]: 1419.9566, found 1419.9640. Calculated for $C_{228}H_{204}N_{36}O_{72}Eu_4F_{36}S_{12}\cdot23H_2O$: C, 40.93; H, 3.77 N, 7.54 %; Found: C, 41.67; H, 3.85; N, 7.51 %. mp: 362–372 °C (decomposition)

Hexa{μ-[*N*^{2},*N*$^{2'}$-(9,10-dioxo-9,10-dihydroanthracene-2,6-diyl) bis{*N*^{6}-[(*R*)-(*sec*-butyl)pyridine-2,6-dicarboxamide]}]}tetrayttrium(III) dodecatriflate, [Y$_4$(L2^{RR})$_6$](CF$_3$SO$_3$)$_{12}$ and

Hexa{μ-[*N*^{2},*N*$^{2'}$-(9,10-dioxo-9,10-dihydroanthracene-2,6-diyl) bis{*N*^{6}-[(*R*)-(*sec*-butyl)pyridine-2,6-dicarboxamide]}]}tetrayttrium(III) dodecatriflate, [Y$_4$(L2^{SS})$_6$](CF$_3$SO$_3$)$_{12}$

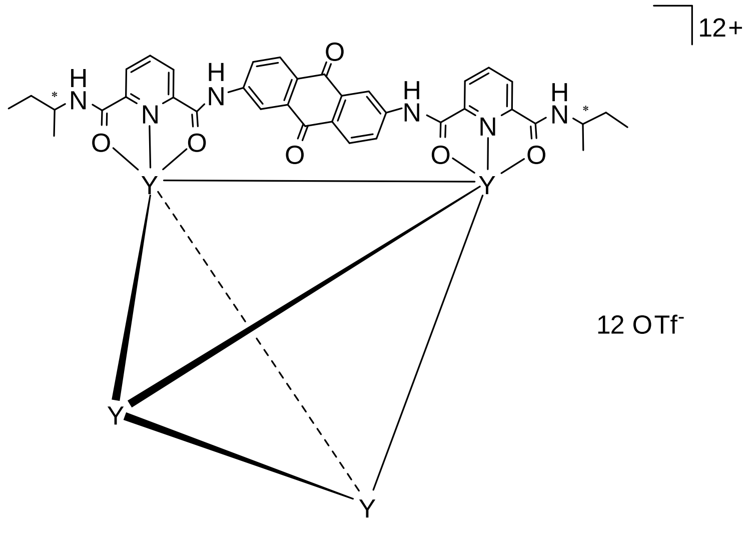

To a white suspension of (**L2^{RR}**) (0.03 g, 0.046 mmol, 1.5 equiv.) in a mixture of 8 mL DCM/MeOH (12:1, v/v), a solution of $Y(CF_3SO_3)_3$ (0.017 g, 0.031 mmol, 1 equiv.) in 12 mL MeCN was added. The suspension was changed to yellow turbidity. The reaction mixture was then refluxed for 1 h and the solid gradually dissolved to give a resulting homogeneous yellow solution. The solvent was removed under reduced pressure. Then crude product was re-dissolved in MeCN and then recrystallized by slow diffusion of diethyl ether to give the desired product. **$[Y_4(L2^{RR})_6](CF_3SO_3)_{12}$**: (0.036 g, 0.006 mmol, 77% yield). ^{1}H NMR (400 MHz, CD_3CN, some of the peaks show two sets of peaks, **A** and **B**, respectively in ~ 1.14:1 ratio, δ): 0.55 (t, *J* = 8 Hz, 6 × 6H, **CH_2CH_3, A**), 0.86 (t, *J* = 4 Hz, 6 × 6H, **CH_2CH_3, B**), 0.97 (d, *J* = 4 Hz, 6 × 6H, **$CHCH_3$, B**), 1.20 (d, *J* = 8 Hz, 6 × 6H, **$CHCH_3$, A**), 1.33–1.41 (quin, *J* = 8 Hz, 6 × 4H, **CH_2, A**), 1.48–1.59 (m, 6 × 4H, **CH_2, B**), 3.58–3.65 (m, 6 × 2H, **$CHCH_3$, B**), 3.65–3.70 (m, 6 × 2H, **$CHCH_3$, A**), 8.03 (d, *J* = 8 Hz, 6 × 2H, **A** and 6 × 2H, **B**), 8.16 (d, *J* = 8 Hz, 6 × 2H, **A** and 6 × 2H, **B**), 8.48–8.56 (m, 6 × 6H, **A** and 6 × 8H, **B**), 8.60 (d, *J* = 8 Hz, 6 × 2H, **N*H*, A**), 9.03–9.07 (m, 6 × 2H, **A** and 6 × 2H, **B**), 10.88–10.89 (d, *J* = 6 Hz, 6 × 2H, **N*H*, A** and 6 × 2H, **N*H*, B**). ^{13}C NMR (CD_3CN, some of the peaks are shown into two sets of peaks, δ): 11.32 (**CH_2CH_3, A**), 11.53 (**CH_2CH_3, B**), 19.61 (**$CHCH_3$, B**), 19.96 (**$CHCH_3$, A**), 29.62 (**CH_2, A**), 29.72 (**CH_2, B**), 51.73 (**$CHCH_3$, A**), 51.85 (**$CHCH_3$, B**), 119.22 (*C*H, it is buried underneath the residual acetonitrile peak), 119.25 (*C*H, it is buried underneath the residual acetonitrile peak), 127.76 (*C*H), 127.85 (*C*H), 127.88 (*C*H), 127.93 (*C*H), 128.35 (*C*H), 128.37 (*C*H), 130.10 (*C*H), 131.98, 135.30, 135.33, 143.24, 143.27, 144.25 (*C*H), 148.50, 148.63, 149.44, 149.56, 168.17, 168.20, 168.35, 168.37, 168.41, 168.43, 182.65. HRMS (ESI) calcd. for $C_{224}H_{204}Y_4F_{24}N_{36}O_{60}S_8$ [M – 4OTf$^-$]: 1356.1903, found 1356.1839. Calculated for $C_{228}H_{204}N_{36}O_{72}Y_4F_{36}S_{12}\cdot20H_2O$: C, 42.89; H, 3.85; N, 7.90 %; Found: C, 42.80; H, 3.83; N, 7.84 %. mp: 372–379 ^{o}C (decomposition); **$[Y_4(L2^{SS})_6](CF_3SO_3)_{12}$** was synthesized, following the procedure for **$[Y_4(L2^{RR})_6](CF_3SO_3)_{12}$** with the use of (**L2^{SS}**) instead, in 71% yield (0.033 g, 0.005 mmol): ^{1}H NMR (400 MHz, CD_3CN, some of the signals are shown in two sets of peaks, **A** and **B**, respectively in ~ 1.16:1 ratio, δ): 0.55 (t, *J* = 8 Hz, 6 × 6H, **CH_2CH_3, A**), 0.87 (t, *J* = 4 Hz, 6 × 6H, **CH_2CH_3, B**), 0.97 (d, *J* = 5 Hz, 6 × 6H, **$CHCH_3$, B**), 1.19 (d, *J* = 6 Hz, 6 × 6H, **$CHCH_3$, A**), 1.34–1.41 (quin, *J* = 8 Hz, 6 × 4H, **CH_2, A**), 1.48–1.61 (m, 6 × 4H, **CH_2, B**), 3.58–3.65 (m, 6 × 2H, **$CHCH_3$, B**), 3.65–3.70 (m, 6 × 2H, **$CHCH_3$, A**), 8.03 (d, *J* = 8 Hz, 6 × 2H, **A** and 6 × 2H, **B**), 8.15 (d, *J* = 8 Hz, 6 × 2H, **A** and 6 × 2H, **B**), 8.48–8.55 (m, 6 × 6H, **A** and 6 × 8H, **B**), 8.60 (d, *J* = 8 Hz, 6 × 2H, **N*H*, A**), 9.05–9.07 (m, 6 × 2H, **A** and 6 × 2H, **B**), 10.88–10.89 (d, *J* = 6 Hz, 6 × 2H, **A** and 6 × 2H, **B**). ^{13}C NMR (CD_3CN, some of the peaks are shown into two sets of peaks, δ): 11.33 (**CH_2CH_3, A**), 11.55 (**CH_2CH_3, B**), 19.62 (**$CHCH_3$, B**), 19.97 (**$CHCH_3$, A**), 29.61 (**CH_2, A**), 29.74 (**CH_2, B**), 51.74 (**$CHCH_3$,**

A), 51.86 (***C*HCH₃, B**), 119.23 (*C*H, it is buried underneath the residual acetonitrile peak), 119.26 (*C*H, it is buried underneath the residual acetonitrile peak), 127.77 (*C*H), 127.85 (*C*H), 127.89 (*C*H), 127.94 (*C*H), 128.37 (*C*H), 128.39 (*C*H), 130.11 (*C*H), 131.99, 135.31, 135.34, 143.26, 143.29, 144.25 (*C*H), 148.52, 148.65, 149.45, 149.57, 168.17, 168.21, 168.36, 168.38, 168.42, 168.44, 182.66. HRMS (ESI) calcd. for $C_{224}H_{204}Y_4F_{24}N_{36}O_{60}S_8$ [M – 4OTf$^-$]: 1356.1903, found 1356.1845. Calculated for $C_{228}H_{204}N_{36}O_{72}Y_4F_{36}S_{12}\cdot 19H_2O$: C, 43.13; H, 3.81; N, 7.94 %; Found: C, 43.09; H, 3.79; N, 7.93 %. mp: 371–377 ^{o}C (decomposition).

***N*^{2},*N*$^{2'}$-(9,10-dioxo-9,10-dihydroanthracene-2,6-diyl)bis{*N*^{6}-[(*R*)-(2-phenylpropyl)pyridine-2,6-dicarboxamide]} (L3^{RR}) and *N*^{2},*N*$^{2'}$-(9,10-dioxo-9,10-dihydroanthracene-2,6-diyl)bis{*N*^{6}-[(*S*)-(2-phenylpropyl)pyridine-2,6-dicarboxamide]} (L3^{SS})**

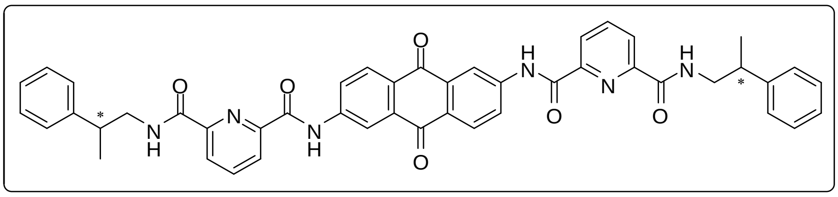

To a stirred solution of **3^R** (0.52 g, 1.84 mmol, 2.2 equiv.) in anhydrous DMF (16 mL) at room temperature, HATU (0.95 g, 2.50 mmol, 3.0 equiv.) was added under nitrogen. After allowing it to stir for 20 min, a 2,6-diaminoanthraquinone (0.20 g, 0.83 mmol, 1.0 equiv.) was added and the reaction mixture was allowed to stir for 20 min in dark. DIPEA (0.87 mL, 4.98 mmol, 6.0 equiv.) was then added to the reaction mixture and the resulting solution was stirred at room temperature for 12 h. The reaction mixture was then diluted with H_2O (50 mL) and extracted with DCM (5 × 30 mL). After removing all of the organic volatile under reduced pressure, the residue was diluted with ethyl acetate (50 mL) and then washed with H_2O (5 × 30 mL) to remove the DMF residual. The organic layer was separated and then concentrated directly under reduced pressure. The residue was then diluted with MeCN (20 mL), and fine powder was progressively precipitated out. Then the solid was collected by filtration and the desired compound was isolated. (**L3^{RR}**): (0.47 g, 0.61 mmol, 74% yield), ^{1}H NMR (400 MHz, d-DMF, δ), 1.48 (d, *J* = 8 Hz, 6H), 3.34 (q, *J* = 8 Hz, 2H), 3.77 (t, *J* = 8Hz, 4H), 7.35–7.39 (m, 2H), 7.48–7.54 (m, 8H), 8.48–8.55 (m, 6H), 8.58–8.62 (m, 4H), 8.92 (d, *J* = 4Hz, 2H), 9.62 (t, *J* = 4 Hz, 2H), 11.38 (s, 2H). ^{13}C NMR (d-DMF, δ): 19.58, 40.40, 47.00, 118.35, 125.70, 125.77, 126.04, 127.03, 127.91, 128.80, 129.98, 135.36, 140.50, 144.73, 145.65, 149.14, 150.41, 163.07, 163.73, 182.30. (**L3^{SS}**) was

synthesized, following the procedure for (**L3**^{RR}) with the use of **3**^{S} instead, in 95% yield (0.61 g, 0.78 mmol): ^{1}H NMR (400 MHz, d-DMF, δ): 1.48 (d, *J* = 8 Hz, 6H), 3.34 (q, *J* = 8 Hz, 2H), 3.76 (t, *J* = 8Hz, 4H), 7.36–7.39 (m, 2H), 7.48–7.54 (m, 8H), 8.48–8.55 (m, 6H), 8.58–8.63 (m, 4H), 8.92 (d, *J* = 4Hz, 2H), 9.63 (t, *J* = 4 Hz, 2H), 11.39 (s, 2H). ^{13}C NMR (d-DMF, δ): 19.50, 40.30, 46.86, 118.22, 125.62, 125.68, 125.86, 126.94, 127.81, 128.89, 129.00, 129.88, 135.25, 140.43, 144.57, 145.55, 149.03, 150.29, 163.24, 163.89, 182.17.

Hexa{μ-[*N*^{2},*N*$^{2'}$-(9,10-dioxo-9,10-dihydroanthracene-2,6-diyl)bis{*N*^{6}-[(*R*)-(2-phenylpropyl)pyridine-2,6-dicarboxamide]}]}tetraeuropium(III) dodecatriflate, [Eu$_4$(L3^{RR})$_6$](CF$_3$SO$_3$)$_{12}$ and Hexa{μ-[*N*^{2},*N*$^{2'}$-(9,10-dioxo-9,10-dihydroanthracene-2,6-diyl)bis{*N*^{6}-[(*S*)-(2-phenylpropyl)pyridine-2,6-dicarboxamide]}]}tetraeuropium(III) dodecatriflate, [Eu$_4$(L3^{SS})$_6$](CF$_3$SO$_3$)$_{12}$

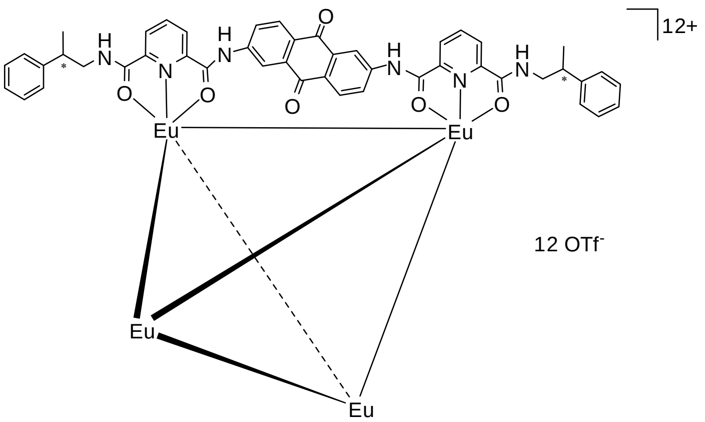

To a white suspension of (**L3**^{RR}) (0.050 g, 0.065 mmol, 1.5 equiv.) in a mixture of 14 mL DCM/MeOH (12:1, v/v), a solution of Eu(CF$_3$SO$_3$)$_3$ (0.026 g, 0.043 mmol, 1 equiv.) in 15 mL MeCN was added. The suspension was changed to yellow turbidity gradually. The reaction mixture was then refluxed for 1 h and the solid slowly dissolved to give a resulting homogeneous yellow solution. The solvent was removed under reduced pressure. Then crude product was re-dissolved in MeCN and then recrystallized by slow diffusion of diethyl ether to give the desired product. **[Eu$_4$(L3^{RR})$_6$](CF$_3$SO$_3$)$_{12}$**: (0.067 g, 0.009 mmol, 88% yield). ^{1}H NMR (400 MHz, CD$_3$CN, some of the peaks are shown two sets of peaks, **A** and **B**, respectively in ~1.21:1 ratio, δ): 1.23 (d, br., *J* = 4 Hz, **6 × 6H, CH$_2$C*H$_3$*, B**), 2.23 (overlap with H$_2$O, **6 × 6H, CHC*H$_3$*, A**), 3.38 (s, br., **6 × 2H, C*H*CH$_3$, B)** 3.70 (s, br., **6 × 2H, C*H*CH$_3$, A**), 4.32 (s, br., **6 × 2H, N*H*, B**), 4.55 (s, br., **6 × 2H, N*H*, A**), 4.72 (s, br., **6 × 2H, C*H*H, A** and **6 × 2H, C*H*H, B**), 5.06 (s, br., **6 × 2H, CH*H*, B**), 5.40 (s, br., **6 × 2H, CH*H*, A**), 5.59 (d, br., *J* = 8 Hz, **6 × 2H, B**), 5.64

(d, br., *J* = 8 Hz, **6 × 2H, A**), 6.09 (d, br., *J* = 8 Hz, **6 × 2H, B**), 6.19 (d, br., *J* = 8 Hz, **6 × 2H, A**), 6.51 (t, br., *J* = 8 Hz, **6 x 2H, B**), 6.56 (t, br., *J* = 8 Hz, **6 x 2H, A**), 7.26–7.29 (m, br., **6 x 4H, A** and **6 x 4H, B**), 7.36 (s, br., **6 x 2H, N*H*, A** and **6 x 2H, B**), 7.42–7.72 (m, br., **6 x 6H, A** and **6 x 6H, B**), 8.68–8.71 (m, br., **6 x 2H, A** and **6 x 2H, B**), 9.31–9.36 (m, br., **6 x 2H, A** and **6 x 2H, B**), 12.13 (s, br., **6 x 2H, A**), 12.33 (s, br., **6 x 2H, B**). ^{13}C NMR (CD_3CN, some of the peaks are shown two sets of peaks, δ): 20.18 (***C*H$_3$, B**), 20.69 (***C*H$_3$, A**), 42.29 (***C*HCH$_3$, B**), 42.62 (***C*HCH$_3$, A**), 49.01 (***C*H$_2$, B**), 49.32 (***C*H$_2$, A**), 92.25 (*C*H), 92.43 (*C*H), 94.05 (*C*H), 118.79 (*C*H), 123.97 (*C*H), 124.22 (*C*H), 125.37, 128.23 (*C*H), 128.63 (*C*H), 128.69 (*C*H, **phenyl-*C*, A**), 128.94 (*C*H, **phenyl-*C*, B**), 129.19 (*C*H), 129.23 (*C*H), 130.11 (*C*H, **phenyl-*C*, A**, 130.49 (*C*H, **phenyl-*C*, B**), 130.55 (*C*H), 130.94 (*C*H), 134.15, 134.23, 135.64, 136.14, 137.04, 137.12, 139.58, 139.90, 144.44, 144.48, 145.38, 155.31 (*C*H), 155.40 (*C*H), 157.03, 157.41, 165.62, 166.33, 184.35, 184.42. HRMS (ESI) calcd. for $C_{284}H_{228}Eu_4F_{24}N_{36}O_{60}S_8$ [M – 4OTf$^-$]: 1606.2527, found 1606.2539. Calculated for $C_{288}H_{228}N_{36}O_{72}Eu_4F_{36}S_{12}\cdot 19H_2O$: C, 46.97; H, 3.64; N, 6.85%; Found: C, 46.29; H, 3.72; N, 6.69 %. mp: 316–321 ^{o}C (decomposition); **[Eu$_4$(L3^{SS})$_6$](CF$_3$SO$_3$)$_{12}$** was synthesized, following the procedure for **[Eu$_4$(L3^{RR})$_6$](CF$_3$SO$_3$)$_{12}$** with the use of (**L3^{SS}**) instead, in 81 % yield (0.061 g, 0.009 mmol): ^{1}H NMR (400 MHz, CD_3CN, some of the signals are shown in two sets of peaks, **A** and **B**, respectively in ~ 1.22:1 ratio, δ): 1.23 (d, br., *J* = 4 Hz, **6 × 6H, CH$_2$C*H$_3$*, B**), 2.23 (overlap with H_2O, **6 × 6H, CHC*H$_3$*, A**), 3.40 (s, br., **6 × 2H, C*H*CH$_3$, B)**, 3.69 (s, br., **6 × 2H, C*H*CH$_3$, A**), 4.38 (s, br., **6 × 2H, N*H*, B**), 4.60 (s, br., **6 × 2H, N*H*, A**), 4.71 (s, br., **6 × 2H, C*H*H, A** and **6 × 2H, C*H*H, B**), 5.05 (s, br., **6 × 2H, CH*H*, B**), 5.39 (s, br., **6 × 2H, CH*H*, A**), 5.61 (s, br., **6 × 2H, B**), 5.66 (s, br., **6 × 2H, A**), 6.11 (s, br., **6 × 2H, B**), 6.20 (s, br., **6 × 2H, A**), 6.51 (s, br., **6 x 2H, B**), 6.57 (s, br., **6 x 2H, A**), 7.26–7.29 (m, br., **6 x 4H, A** and **6 x 4H, B**), 7.38 (s, br., **6 x 2H, N*H*, A** and **6 x 2H, B**), 7.44–7.71 (m, br., **6 x 6H, A** and **6 x 6H, B**), 8.67–8.71 (m, br., **6 x 2H, A** and **6 x 2H, B**), 9.31–9.36 (m, br., **6 x 2H, A** and **6 x 2H, B**), 12.11 (s, br., **6 x 2H, A**), 12.31 (s, br., **6 x 2H, B**). ^{13}C NMR (CD_3CN, some of the peaks are shown into two sets of peaks, δ): 20.19 (***C*H$_3$, B**), 20.71 (***C*H$_3$, A**), 42.27 (***C*HCH$_3$, B**), 42.59 (***C*HCH$_3$, A**), 49.06 (***C*H$_2$, B**), 49.36 (***C*H$_2$, A**), 92.83 (*C*H), 93.01 (*C*H), 94.64 (*C*H), 119.38 (*C*H), 123.87 (*C*H), 124.11 (*C*H), 125.41, 128.26 (*C*H), 128.65 (*C*H), 128.71 (*C*H, **phenyl-*C*, A**), 128.95 (*C*H, **phenyl-*C*, B**), 129.18 (*C*H), 129.21 (*C*H), 130.14 (*C*H, **phenyl-*C*, A**), 130.52 (*C*H, **phenyl-*C*, B**), 130.56 (*C*H), 130.95 (*C*H), 134.13, 134.21, 136.07, 136.54, 137.02, 137.11, 139.98, 140.29, 144.45, 144.48, 145.40, 155.24 (*C*H), 155.31 (*C*H), 157.36, 157.70, 165.84, 166.54, 184.33, 184.40. HRMS (ESI) calcd. for $C_{284}H_{228}Eu_4F_{24}N_{36}O_{60}S_8$ [M – 4OTf$^-$]: 1606.2527, found 1606.2540. Calculated for $C_{288}H_{228}N_{36}O_{72}Eu_4F_{36}S_{12}\cdot 20H_2O$: C, 46.86; H, 3.66; N, 6.83%; Found: C, 47.21; H, 3.73; N, 6.86 %. mp: 314–322 ^{o}C (decomposition).

Hexa{μ-[N^2,$N^{2'}$-(9,10-dioxo-9,10-dihydroanthracene-2,6-diyl)bis{N^6-[(*R*)-(2-phenylpropyl)pyridine-2,6-dicarboxamide]}]}tetrayttrium(III) dodecatriflate, $[Y_4(L3^{RR})_6](CF_3SO_3)_{12}$ and
Hexa{μ-[N^2,$N^{2'}$-(9,10-dioxo-9,10-dihydroanthracene-2,6-diyl)bis{N^6-[(*S*)-(2-phenylpropyl)pyridine-2,6-dicarboxamide]}]}tetrayttrium(III) dodecatriflate, $[Y_4(L3^{SS})_6](CF_3SO_3)_{12}$

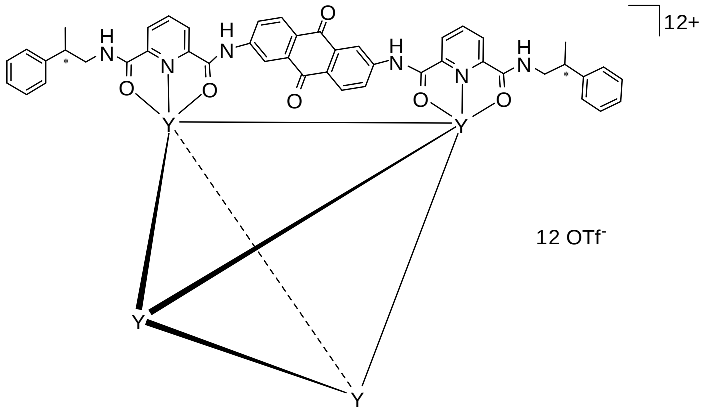

To a white suspension of (**L3^RR^**) (0.041 g, 0.053 mmol, 1.5 equiv.) in a mixture of 10 mL DCM/MeOH (12:1, v/v), a solution of $Y(CF_3SO_3)_3$ (0.019 g, 0.035 mmol, 1 equiv.) in 14 mL MeCN was added. The suspension was changed to yellow turbidity. The reaction mixture was then refluxed for 1 h and the solid slowly dissolved to give a resulting homogeneous yellow solution. The solvent was removed under reduced pressure. Then crude product was re-dissolved in MeCN and then recrystallized by slow diffusion of diethyl ether to give the desired product. **$[Y_4(L3^{RR})_6](CF_3SO_3)_{12}$**: (0.048 g, 0.007 mmol, 81% yield). ^{1}H NMR (400 MHz, CD_3CN, some of the signals are shown in two sets of peaks, **A** and **B**, respectively in ~ 1.02:1 ratio, δ): 0.93 (d, *J* = 7 Hz, 6 × 6H, **CHC*H*₃, B**), 1.13 (d, *J* = 7 Hz, 6 × 6H, **CHC*H*₃, A**), 2.69–2.77 (m, 6 × 2H, **C*H*CH₃, B**), 2.79–2.88 (m, 6 × 2H, **C*H*CH₃, A**), 3.21–3.31 (m, 6 × 2H, **C*H*H**, **A** or **B**), 3.30–3.37 (m, 6 × 2H, **CH*H***, **A** and 6 × 2H, **CH*H***, **B**), 3.42–3.54 (m, 6 × 2H, **C*H*H**, **A** or **B**), 6.94–6.99 (m, 6 × 4H, **phenyl-*H***, **A** or **B**), 7.03 (d, *J* = 7 Hz, 6 × 4H, **phenyl-*H***, **A** or **B**), 7.15–7.29 (m, 6 × 12H, **phenyl-*H***, **A** or **B**), 8.07 (d, *J* = 8 Hz, 6 × 4H, **A** or **B**), 8.15–8.21 (m, 6 × 4H, **A** or **B**), 8.40–8.58 (m, 6 × 6H, **A** and 6 × 6H, **B**), 8.98–9.08 (m, 6 × 2H, **N*H***, **A** and 6 × 2H, **B**), 9.08–9.16 (m, 6 × 4H, **A** or **B**), 10.93 (s, 6 × 2H, **N*H* , A**), 10.99 (s, 6 × 2H, **N*H*, B**). ^{13}C NMR (CD_3CN, some of the peaks are shown into two sets of peaks, δ): 19.73 (***C*H₃, A**), 19.85 (***C*H₃, B**), 40.37 (**CHCH₃, B**), 40.41 (***C*HCH₃, A**), 49.30 (*C*H₂, **A** and **B**), 119.14 (*C*H, it is buried underneath the residual acetonitrile peak), 119.34 (*C*H, it is buried underneath the residual acetonitrile peak), 127.65 (*C*H), 127.71 (*C*H), 127.91 (*C*H), 128.22 (*C*H, **phenyl-*C***) 128.32 (*C*H, **phenyl-*C***), 128.37 (*C*H, **phenyl-*C***), 128.77 (*C*H),

128.80 (*C*H), 130.06 (*C*H, **phenyl-*C***), 130.09 (*C*H, **phenyl-*C***), 130.16 (*C*H), 130.20 (*C*H), 132.04, 135.33, 143.15, 143.17, 144.63 (*C*H), 144.67 (*C*H), 144.84, 144.87, 148.68, 149.12, 149.19, 168.29, 168.38, 168.99, 169.12, 182.66. HRMS (ESI) calcd. for $C_{284}H_{228}Y_4F_{24}N_{36}O_{60}S_8$ [M – 4OTf$^-$]: 1542.2373, found 1542.2356. Calculated for $C_{288}H_{228}N_{36}O_{72}Y_4F_{36}S_{12}\cdot 21H_2O$: C, 48.39; H, 3.81; N, 7.05%; Found: C, 49.13; H, 3.80; N, 7.05 %. mp: 320–329 °C (decomposition); **[Y$_4$(L3^{SS})$_6$](CF$_3$SO$_3$)$_{12}$** was synthesized, following the procedure for **[Y$_4$(L3^{RR})$_6$](CF$_3$SO$_3$)$_{12}$** with the use of (**L3^{SS}**) instead, in 82% yield (0.049 g, 0.007 mmol): ^{1}H NMR (400 MHz, CD_3CN, some of the signals are shown in two sets of peaks, **A** and **B**, respectively in ~ 1.02:1 ratio, δ): 0.94 (d, *J* = 7 Hz, 6 × 6H, **CHC*H$_3$***, **B**), 1.14 (d, *J* = 7 Hz, 6 × 6H, **CHC*H$_3$***, **A**), 2.68–2.76 (m, 6 × 2H, **C*H*CH$_3$**, **B**), 2.76–2.89 (m, 6 × 2H, **C*H*CH$_3$**, **A**), 3.20–3.31 (m, 6 × 2H, **C*H*H**, **A** or **B**), 3.31–3.39 (m, 6 × 2H, **CH*H***, **A** and 6 × 2H, **CH*H***, **B**), 3.42–3.54 (m, 6 × 2H, **C*H*H**, **A** or **B**), 6.92–7.00 (m, 6 × 4H, **phenyl-*H***, **A** or **B**), 7.03 (d, *J* = 8 Hz, 6 × 4H, **phenyl-*H***, **A** or **B**), 7.14–7.31 (m, 6 × 12H, **phenyl-*H***, **A** or **B**), 8.07 (d, *J* = 8 Hz, 6 × 4H, **A** or **B**), 8.14–8.22 (m, 6 × 4H, **A** or **B**), 8.40–8.60 (m, 6 × 6H, **A** and 6 × 6H, **B**), 9.01–9.09 (m, 6 × 2H, **N*H***, **A** and 6 × 2H, **B**), 9.09–9.18 (m, 6 × 4H, **A** or **B**), 10.93 (s, 6 × 2H, **N*H*, A**), 10.99 (s, 6 × 2H, **N*H*, B**). ^{13}C NMR (CD_3CN, some of the peaks are shown into two sets of peaks, δ): 19.73 (***C*H$_3$, A**), 19.85 (***C*H$_3$, B**), 40.37 (***C*HCH$_3$, B**), 40.41 (***C*HCH$_3$, A**), 49.30 (*C*H$_2$, **A** and **B**), 119.14 (*C*H, it is buried underneath the residual acetonitrile peak), 119.35 (*C*H, it is buried underneath the residual acetonitrile peak), 127.66 (*C*H), 127.72 (*C*H), 127.90 (*C*H), 128.21 (*C*H, **phenyl-*C***), 128.32 (*C*H, **phenyl-*C***), 128.37 (*C*H, **phenyl-*C***), 128.77 (*C*H), 128.80 (*C*H), 130.06 (*C*H, **phenyl-*C***), 130.09 (*C*H, **phenyl-*C***), 130.17 (*C*H), 130.21 (*C*H), 132.04, 135.33, 143.15, 143.17, 144.64 (*C*H), 144.67 (*C*H), 144.86, 144.88, 148.68, 149.12, 149.20, 168.30, 168.39, 168.99, 169.12, 182.66. HRMS (ESI) calcd. for $C_{284}H_{228}Y_4F_{24}N_{36}O_{60}S_8$ [M – 4OTf$^-$]: 1542.2373, found 1542.2397. Calculated for $C_{288}H_{228}N_{36}O_{72}Y_4F_{36}S_{12}\cdot 19H_2O$: C, 48.64; H, 3.77; N, 7.09 %; Found: C, 48.88; H, 3.73; N, 7.03 %. mp: 322–331 °C (decomposition). *(not all the expected 13C resonances for the complex could be resolved due to poor solubility and significant signal overlap)*
